# Semiconducting Polymers for Neural Applications

**DOI:** 10.1021/acs.chemrev.1c00685

**Published:** 2022-01-28

**Authors:** Ivan B. Dimov, Maximilian Moser, George G. Malliaras, Iain McCulloch

**Affiliations:** † Electrical Engineering Division, Department of Engineering, University of Cambridge, 9 JJ Thomson Avenue, Cambridge CB3 0FA, U.K.; ⊥ University of Oxford, Department of Chemistry, Oxford OX1 3TA, United Kingdom; § King Abdullah University of Science and Technology (KAUST), KAUST Solar Center, Thuwal 23955-6900, Saudi Arabia

## Abstract

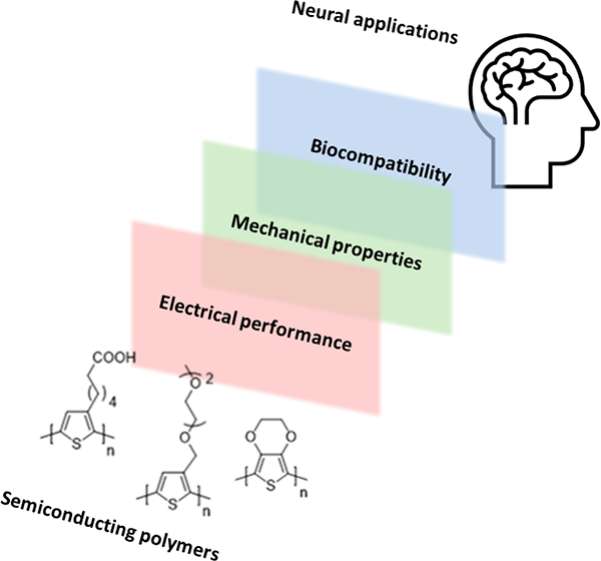

Electronically interfacing with the nervous system for the purposes of health diagnostics and therapy, sports performance monitoring, or device control has been a subject of intense academic and industrial research for decades. This trend has only increased in recent years, with numerous high-profile research initiatives and commercial endeavors. An important research theme has emerged as a result, which is the incorporation of semiconducting polymers in various devices that communicate with the nervous system—from wearable brain-monitoring caps to penetrating implantable microelectrodes. This has been driven by the potential of this broad class of materials to improve the electrical and mechanical properties of the tissue–device interface, along with possibilities for increased biocompatibility. In this review we first begin with a tutorial on neural interfacing, by reviewing the basics of nervous system function, device physics, and neuroelectrophysiological techniques and their demands, and finally we give a brief perspective on how material improvements can address current deficiencies in this system. The second part is a detailed review of past work on semiconducting polymers, covering electrical properties, structure, synthesis, and processing.

## 1 Introduction

Since Galvani’s seminal “twitching frog leg” experiment in the 1780s, marking the origin of the field of bioelectronics, significant advances have been made in the interfacing of neural systems with synthetic materials. Importantly, considerable scientific, technological, and industrial interest still exists today to further advance the state-of-the-art of the field in an expectation to improve healthcare for humanity. Contemporary initiatives aimed at expanding our understanding of neural signaling pathways in animals and humans include the US-led Brain Research through Advancing Innovative Neurotechnologies (BRAIN) initiative launched in 2013 and its European counterpart, the Human Brain Project (HBP). Significant interest has also come from various industrial enterprises, such as Galvani Bioelectronics, a company formed through the partnership between GlaxoSmithKline (GSK) and Verily Life Sciences, a subsidiary of Alphabet Inc., which aims to invest $715 M to advance bioelectronic medicine over the coming years. Alternative highly publicized commercial ventures include Neuralink Corporation, founded by business magnate Elon Musk, and BrainGate, a US start-up company currently owned by Tufts University.

**Figure 1:**
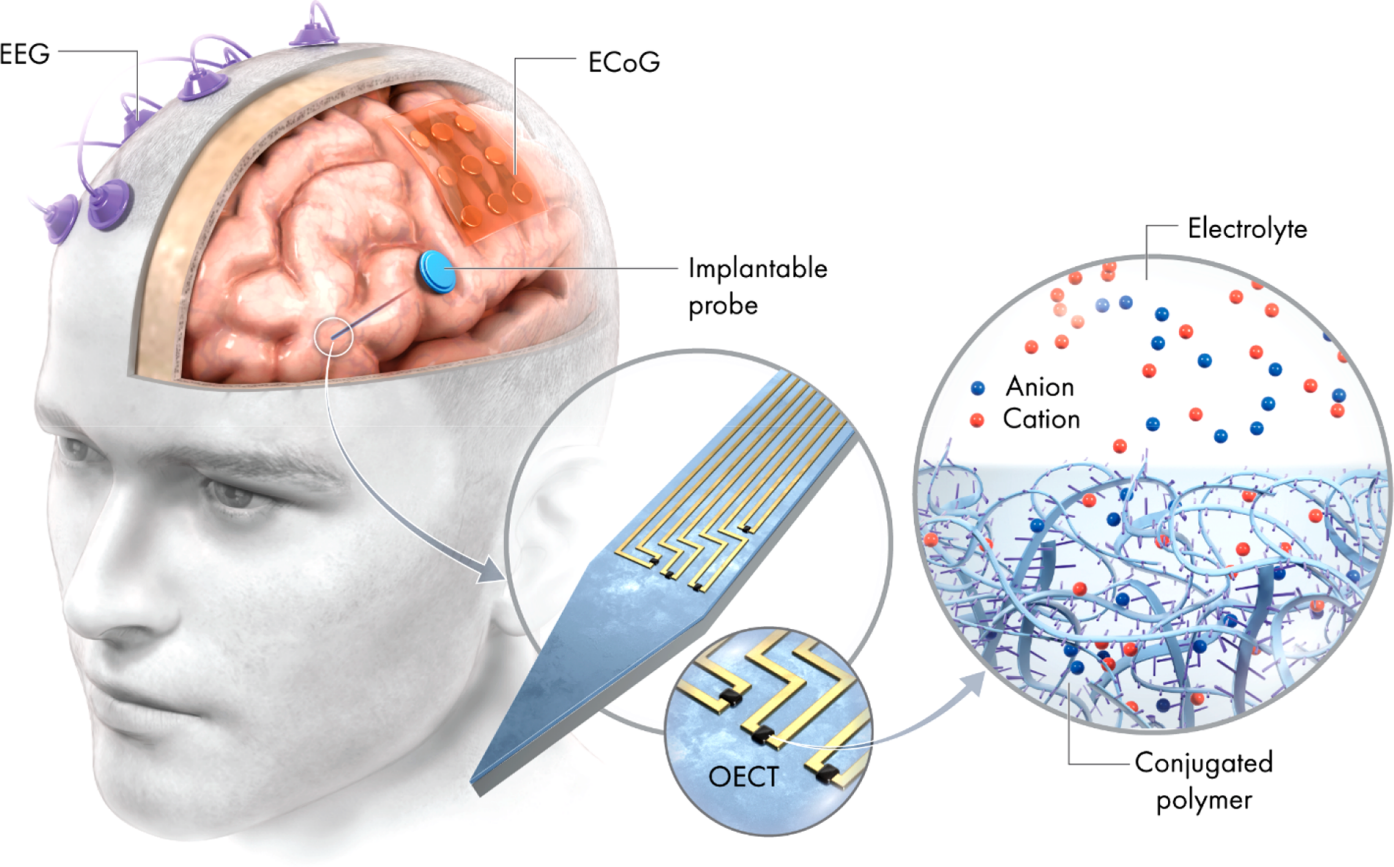
Schematic illustrating various types of *in vivo* neural interfacing, including electroencephalography (EEG), electrocorticography (ECoG), and the use of implantable probes. Close-up of an electronic recording device, in this case an organic electrochemical transistor (OECT) and the interface between the employed conjugated polymer (CP) and the electrolyte present in the biological tissue under study.

To meet the growing demand of such technologies, the past decade has witnessed significant advances in electronic device physics, fabrication, and miniaturization, as famously described by Moore’s law. As such, immense processing power and data-transfer speeds are at the disposal of researchers studying the human brain. However, these strides often cannot be fully exploited, due to the limitations incurred by the materials used to interface with neural systems. Neural activity elicits ionic currents in the cerebrospinal fluid. Their transduction to an electronic signal, usually achieved by a metal electrode such as Pt, is an inefficient process. Moreover, the Young’s modulus of Pt is >100 GPa, which is several orders of magnitude larger than those of neural tissues, which lie in the range 10–10,000 Pa. The significant mismatch in mechanical properties leads to both acute and chronic electrode instability; thus, these materials are not ideal for neural interfacing applications (Figure 1).^1^ Because of these limitations, the past 15 years have witnessed considerable growth and interest in using organic semiconductors and, in particular, organic mixed ionic-electronic conductors (OMIECs) to interface with biological systems.^1−5^ OMIECs can be considered a subset of π-conjugated polymers (CPs) and are particularly suited for the transduction between information carriers in biological systems (typically hydrated ions) and those in semiconductor technologies (electrons), given their (i) oxide-free interfaces and “open” structures enabling bulk electronic transport across the electronic material, (ii) tendency to undergo large structural changes upon ion interaction, (iii) ability to readily be tailored by chemical design, and (iv) softer mechanical properties. The ability of OMIECs to transport electronic charge carriers arises from the overlap of p_*z*_ orbitals on neighboring sp^2^ hybridized atoms, thereby creating an extended π-conjugated system. An electronic charge injected by metal electrodes or chemical doping can be subsequently transported in CPs both intramolecularly along individual CP backbones (fast) and intermolecularly by charge hopping from one molecule to another one (slow).^6^ Depending on the choice of molecular building blocks comprising the CP backbone, CPs can be tailored to transport p- and/or n-type charge carriers. On the other hand, ionic conduction in aqueous media can be instilled into CPs through various means, such as through the grafting of hydrophilic side chains onto the CP backbone or by blending the CP together with a hydrophilic component. The possibilities for synthetic chemists are thus virtually endless.

In this review, we will describe the physics and applications in neurotechnology of organic electrochemical devices, and we will particularly focus on the landscape of available materials. In the interest of completeness, we will also review the use of passive electrodes, due to the significant conceptual overlap between OMIEC-coated electrodes and OECTs. Through this review, we aimed for a self-contained overview of the topic, which would incorporate background information on neuroscience and device engineering and characterization. There are additional reviews on the topic for a more general point of view on semiconducting polymers, to which we can direct the reader.^7,8^ A good review on the fundamentals of neural electrode characterization and performance is the one by Cogan.^9^ An older but very broad and accessible review of various neural applications has been given by Rutten.^10^ A general overview on organic coatings on electrodes has been given by Aregueta-Robles et al.^11^

## 2 The Nervous System: Structure and Signaling

The human nervous system is divided into a central and peripheral part—the CNS, containing the brain and spinal cord, and the PNS, containing the rest of the nerves. It is involved in the control of various autonomous or voluntary actions of the body. A large variety of cells build-up the nervous system. Broadly, they can be categorized into neurons and support cells (typically glia). A typical morphology for neurons includes a cell body, called the soma, with multiple protrusions, called dendrites, coronally protruding therefrom and one particularly long protrusion, known as the axon. The terminus of the axon forms connections with other cells (neurons, muscle, or endocrine cells), known as synapses. This cable-like structure is very conducive to information transfer, as we will cover below.

Neurons are the cells carrying out the bulk of the information transport in the nervous system. Information can be relayed electrically or chemically. In the case of electrical information transfer, changes in the membrane potential of cells are exploited. This is covered extensively in introductory texts but for the sake of completeness will be reviewed here.^12^

### 2.1 Neural Signals

At the cost of metabolic energy, neurons maintain a concentration gradient of different ions (including sodium, potassium, calcium, and chloride) across their membranes. This gives rise to an electrochemical potential difference across the membrane, as predicted by the Nernst equation (and more specifically, the closely related Goldmann equation), known from introductory electrochemistry.^13^ For mammalian neurons, this value is typically around −60 mV (inside vs outside membrane). Small changes to the membrane potential toward 0 mV (i.e., depolarizing) can trigger a response known as an action potential, which consists of a brief (1–2 ms) spike to 150 mV, followed by a brief refractory period during which the original potential is restored at the cost of metabolic energy. Spatially, this process takes place in a small region of the membrane of each neuron, leading to a small propagating area of inverted polarity, known as an action potential. This behavior and its dynamics are determined by the presence of voltage-gated ion channels.

The action potential is propagated spatially until it reaches the junction, or synapse, between two cells. The synapse can be chemical or electrical, depending on whether a chemical messenger is released from the presynaptic cell or if the signal is further propagated electrically. The large majority of synapses in humans are chemical. In a chemical synapse, chemical messengers, known as neurotransmitters, are released by the presynaptic cell and bind to receptors on the postsynaptic cell, leading to some effect (e.g., initiation of excitation of the postsynaptic neuron or muscle contraction or endocrine secretion of a molecule). In this review we will focus on applications that record or stimulate neural activities using electrical means. Biochemical sensing of neurotransmitter release or actuation of neurons via the delivery of biomolecules involves complexities beyond the scope of this review; interested readers can consult recent reviews.^14−16^

As a consequence of the above-described electrical information transport, several types of signals can be observed.^17^ First, one can monitor the millivolt-scale depolarization events in a single cell, in a class of techniques known as intracellular recording. This is beyond the scope of the review, as it is not routinely used in the clinic. Such techniques (e.g., patch clamp), however, are useful in the study of various properties of the cell membrane and of any membrane proteins contained therein—historically, such studies have been among the first to observe individual molecules.^18,19^

There are extracellular ionic currents that are borne out as a consequence of the action potential, which can be observed with suitable recording equipment, as spikes up to 100 μV in magnitude. The exact individual form of each spike is dependent on the distance and orientation of its source with respect to the recording probe. These minor differences are exploited in discriminating the source neuron of each cell.^20^ Such signals are typically referred to as single-units, and the ability to record them has been important in fundamental research on the nervous systems and in exploratory clinical applications, such as prosthetic control. One can also record the cumulative signal of dozens of neurons firing together, leading to what is known as local field potentials (LFPs), which offer an increased signal amplitude, at the cost of reduced resolution. Finally, one can externally record sums of an even greater number of active neurons, by monitoring the resultant voltage on the skin.^13^ Lastly, due to the electrochemical nature of triggering an action potential, this allows for external triggering of it, for example, by electrical stimulation.

## 3 Neuroelectronic Interfaces

Hydrated ions play a major role in conveying biological information, as can be inferred from the description above, while in conventional electronic devices, that role is taken up by electrons. This suggests that some form of transformation between the two is necessary. This process occurs at the interface between a biological tissue under study and a probe. As such, knowledge of the electrochemical properties of an individual electrode and those of its constituent materials is vital in guiding the design and fabrication of electronic devices for neural applications.

The devices capable of this transduction are electrodes and transistors. A conventional electrode is a conductor that merely passively probes the electric field in its immediate vicinity, while a transistor is an active element, where the signal is amplified. Generally, most neurophysiological research is carried out with passive electrodes, and signals thereby acquired are amplified by a dedicated acquisition equipment. This amplification is nowadays carried out in external equipment with semiconductor circuits employed for multiple stages of amplification and transistors. However, in recent developments an individual transistor can be miniaturized and brought into contact with the tissue, acquiring and amplifying signals directly, minimizing the distance that the acquired signal can travel (and thus be subject to noise and attenuation). In this section we will compare electrodes and transistors and introduce the means of characterizing them. The main focus is on transistors, organic electrochemical transistors specifically, and electrodes are introduced due to conceptual similarities between the operation of electrochemical transistors and passive electrodes.

### 3.1 Electrodes

Electrodes in general are conductors that interface a circuit with its nonmetallic surroundings—e.g. tissue in most bioelectronic applications. In such applications, electrodes tend to be corrosion-resistant materials, such as platinum or titanium. Significant research efforts are aimed at improving the electrical and other properties of such metallic electrodes with various coatings, of which conjugated polymers are a prominent representative. Typically, an electrode is characterized electrochemically with several techniques—electrical impedance spectroscopy, cyclic voltammetry, and voltage transient measurements. We will now briefly review these techniques.

#### 3.1.1 Electrode Impedance

EIS (electrochemical impedance spectroscopy) is a widely used electrochemical technique for electrode property characterization. The technique can be used to characterize a specific electrode as a discrete device (and thus give an indication of its quality or inform design of connected devices such as amplifiers) or for fundamental studies on the electrode material. It can provide an easily obtained figure of merit to judge the relative quality of an electrode (e.g., to another electrode or to itself at a different point in time) by providing an impedance value for frequencies in the range of the signal to be recorded. For example, spiking activity from single neurons in recordings consists of 1–2 ms long spikes—as such a measurement of the impedance at 1 kHz gives an idea of how an electrode would perform for that particular purpose.^9^ A low electrode impedance is necessary for high-quality recordings, as it prevents signal attenuation which would otherwise occur, if the electrode impedance exceeds the input impedance of a recording amplifier.^21^ Similarly, in stimulation, a low impedance is also favored, as it implies more efficient power transfer between the electrode and tissue, which in turn minimizes undesirable effects (heating, electrochemical reactions due to faradaic effects).^22^ Lastly, due to the low voltage amplitudes involved in EIS (typically 5–10 mV), it also offers a safe way to monitor electrode quality *in vivo*.

In general, as known from introductory electrochemistry, the behavior of an electrode can be abstracted to standard passive components.^21^ A useful approximate representation for most recording electrodes is a series resistor–capacitor circuit.^23^ The impedance of an electrode typically lowers as its area increases (due to the increase in capacitance of the capacitor) at the cost of a decrease in resolution (as the acquired signal is averaged over a larger area). One of the drivers of the development of various coatings for such electrodes is their rougher surface, providing an increased electrochemical surface area (ESA), for the same geometric surface area (GSA), mitigating the above trade-off. This concept is extended in OMIECs, such as PEDOT:PSS, which exhibit a property, known as “volumetric capacitance”, where due to their ability to conduct both ions and electrons, their capacitance at an electrode–electrolyte interface depends on the film volume overall, as opposed to solely the area.

#### 3.1.2 Cyclic Voltammetry

While recording from an electrode typically occurs near the open-circuit potential and at very small current densities, during stimulation, electrodes can be hundreds of millivolts away from that potential. In assessing an electrode’s suitability for stimulating, we must know its behavior at wider potential ranges. EIS typically gives us information over a small voltage span and a large frequency range, while cyclic voltammograms can reveal the presence of faradaic processes (by showing peaks in the voltammogram) and the electrochemical windows for a given electrode–electrolyte combination (i.e., the range of potentials within which there is no electrolysis of water). Faradaic processes such as water electrolysis or electrode corrosion would be very undesirable during electrical stimulation of tissue.

The voltammogram of a good material for a neural electrode would thus show an entirely capacitive contribution, with no peaks that might indicate faradaic reactions, which could result in generation of harmful species or electrode corrosion.

Additionally, cyclic voltammetry reveals the charge storage capacity of an electrode, which is an important stimulation electrode benchmark. Derived as the time integral of the current in a CV measurement, spanning the electrochemical window, it gives the available physical charge on an electrode for stimulation.^24^

### 3.2 Transistors

#### 3.2.1 General Description of Transistors

**Figure 2:**
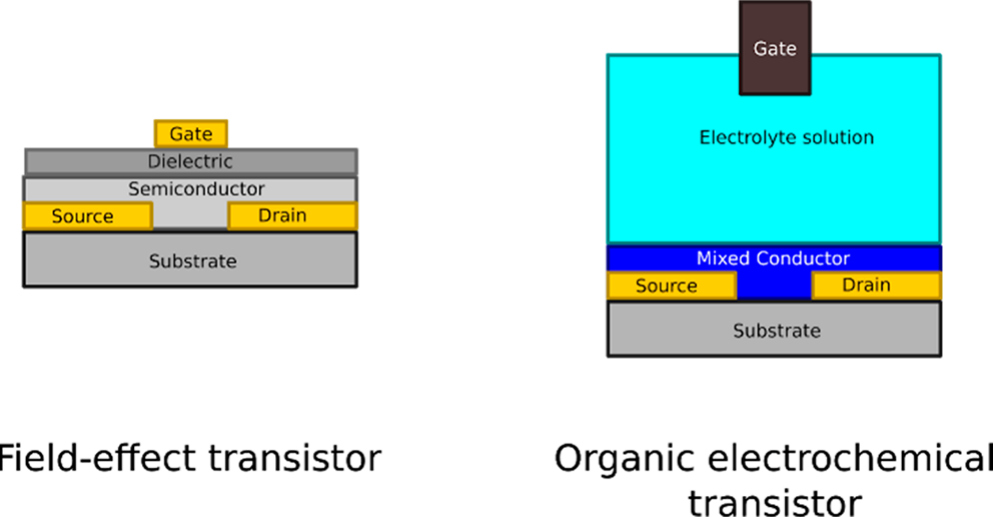
Comparison of a typical field-effect transistor (FET) and organic electrochemical transistor (OECT) architecture.

Generally, transistors consist of three conducting terminals—conventionally called source, drain, and gate for the most common types of transistors (so-called field-effect transistors or FETs). In a common configuration of these devices, semiconducting material is deposited over the source and drain contacts, and the part between the two electrodes defines the so-called channel. Typically, the metal contacts are deposited using a physical vapor deposition technique (such as thermal evaporation), in a lithographically defined pattern. The semiconducting material can also be patterned, whether by direct inkjet printing, lithography, or selective etching. A layer of dielectric is subsequently deposited (in the case of FETs), and the gate electrode is then patterned on top of that dielectric, ensuring a capacitive coupling between the gate and channel (Figure 2). This is the so-called top-gate bottom-contact architecture. Other configurations are also possible—for example the gate and dielectric can be at the bottom of the stack, and the source and drain on the top (so-called bottom-gate top-contact), or all three electrodes could be at the bottom (bottom-gate bottom-contact).

A transistor can act as an electronically controlled switch or as an amplifier. Typically, a voltage is applied to the gate electrode, which changes the conductivity of the semiconducting channel. This allows the control of the current that flows between the source and drain electrodes, forming the basis of a transistor’s function as an amplifier or a switch. This modulation of conductivity is also the basis for the name of the device—a transfer resistor. Traditionally, commercially available transistors can be classified by many different characteristics. The charge carrier of the transistor is identified by referring to it as either n-type or p-type (for electrons or electron vacancies , a.k.a. “holes”, respectively), and its mode of operation can be “accumulation” (normally off, switches on when biased) or “depletion” (normally on, switches off when biased).

In the case of FETs, channel conductivity is modulated by a field-effect changing the charge density inside the semiconductor near the interface with the gate dielectic.^25^ In recent years, however, the development of mixed-conductor materials, such as polyethylenedioxythiophene:polystyrenesulfonate (PEDOT:PSS), has led to an explosion of interest in devices known as organic electrochemical transistors (OECTs) and their application in biosensing.^26−28^ In an OECT, the device structure is changed—the gate electrode and semiconducting channel are both immersed in an electrolyte, and there is no dielectric layer on the channel. Applying a voltage on the gate electrode changes the doping level of the semiconductor, through the injection of ions from the electrolyte into the channel. For example, in a typical OECT device, the semiconducting layer is made of PEDOT:PSS, which is a p-type material (carriers are positively charged cationic moieties along the backbone). PEDOT itself is a semiconducting polymer, and PSS is a dopant and an ionic conductor. As such, when a positive voltage is applied to the gate, it leads to cations penetrating the polymer. This compensates the sulfonate anions on the PSS, via hole extraction at the drain (i.e., electron transfer between the metal contact and the semiconductor). In turn this leads to lower doping and reduced electrical conductivity in the PEDOT domains, leading to depletion mode behavior.

As mentioned above, OECTs operate based on doping and dedoping of a semiconducting channel, using ions. This is beneficial, in terms of biosensing applications, as it allows (and even exploits) functioning in an environment rich in electrolytes, such as serum. While a drawback to this is the typically slow device operation, recently published strategies, where ions are incorporated into solvated reservoirs in the conducting channel, have demonstrated significant increases in operating speed.^29,30^

#### 3.2.2 Characterizing Transistors

Typically, transistors are characterized by acquiring their steady-state characteristics, including their output and transfer curves, and by applying an AC signal, such as a square pulse or sine wave, for deriving their transient characteristics. AC characterization is often necessary as a lot of transistor parameters are frequency dependent. In practice, the DC measurements are done with dedicated instruments known as source-meter units (SMUs) or semiconductor parameter analyzers (SPAs). AC characterization data can be reported using the same instruments or often by adapting potentiostats or combinations of signal generators and oscilloscopes.

Transistor characteristics and ways to derive them will be reviewed next. Most of the transistor characteristics have been defined for traditional inorganic semiconductors; however, if we gloss over the molecular-scale details of the operation of different transistor types, the same quantitative descriptors can be applied. Introductory texts in microelectronics offer excellent coverage of the topic.^25^

##### 3.2.2.1 Transfer Curves

**Figure 3:**
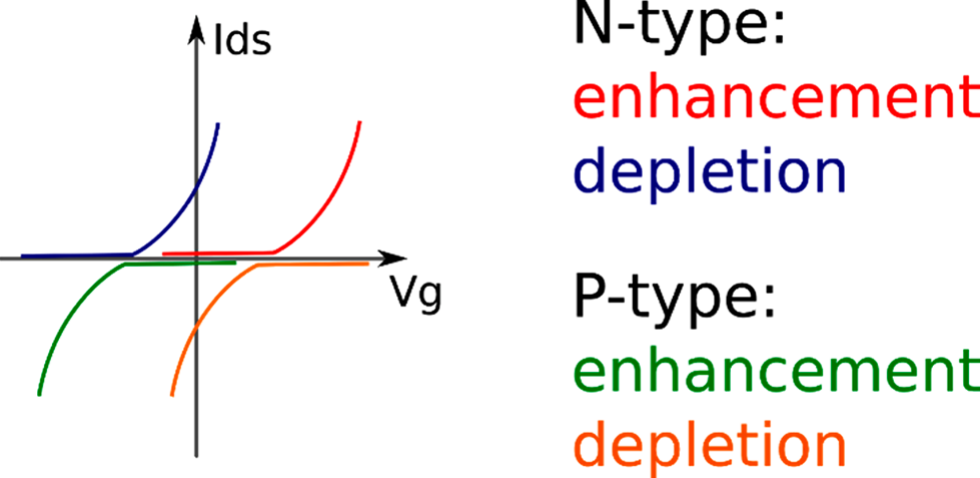
Representative transfer curve shapes for p- and n-type transistors, operating in depletion and enhancement mode.

One can scan the gate-source voltage, *V*_gs_, for a given drain-source voltage, *V*_ds_, and monitor the drain current, *I*_ds_, and the leakage current across the gate, *i*_gs_ (the source terminal is typically grounded, i.e. at 0 V). The resultant curve is known as the transfer curve. This curve yields several characteristics such as the on and off drain currents, *i*_on_ and *i*_off_, respectively, or by taking the first derivative of the transfer curve, the transconductance, *g*_m_, of the device, as a function of gate voltage, can be obtained. *g*_m_ is an especially important quantity for applications of a transistor as a transimpedance amplifier (i.e., where a voltage signal is converted and amplified to a current signal), and especially so its maximum value, *g*_max_, and the voltage at which it occurs, *V*_*g*_max__. Significant efforts have gone into engineering OECTs with *g*_max_ at *V*_gs_ = 0,^26^ due to the benefits this brings in simplifying circuitry and minimizing the need for continuously biasing a gate electrode. A further value that can be extracted is the threshold voltage, *V*_th_. The threshold voltage is broadly defined as the minimum gate voltage required for conduction of the source-drain channel. Its physical significance is broadly the voltage required between the gate and semiconducting channel, in order to have a sufficient number of charge carriers in the channel to achieve conduction. Finally, a materials parameter that can be determined is the electronic charge carrier mobility, μ, which determines the drift velocity of electronic charge carriers in an electric field. It should be noted, however, that extracting *V*_th_ and carrier mobilities in an accurate, artifact-free method is still an object of active research in its own right.^31^ Finally, the overall shape and position of the transfer curve with respect to the origin of the *V*_gs_–*I*_ds_ coordinate system informs us whether a device is p-type or n-type and whether it operates in accumulation or depletion mode (as shown in Figure 3).

##### 3.2.2.2 Output Curves

**Figure 4:**
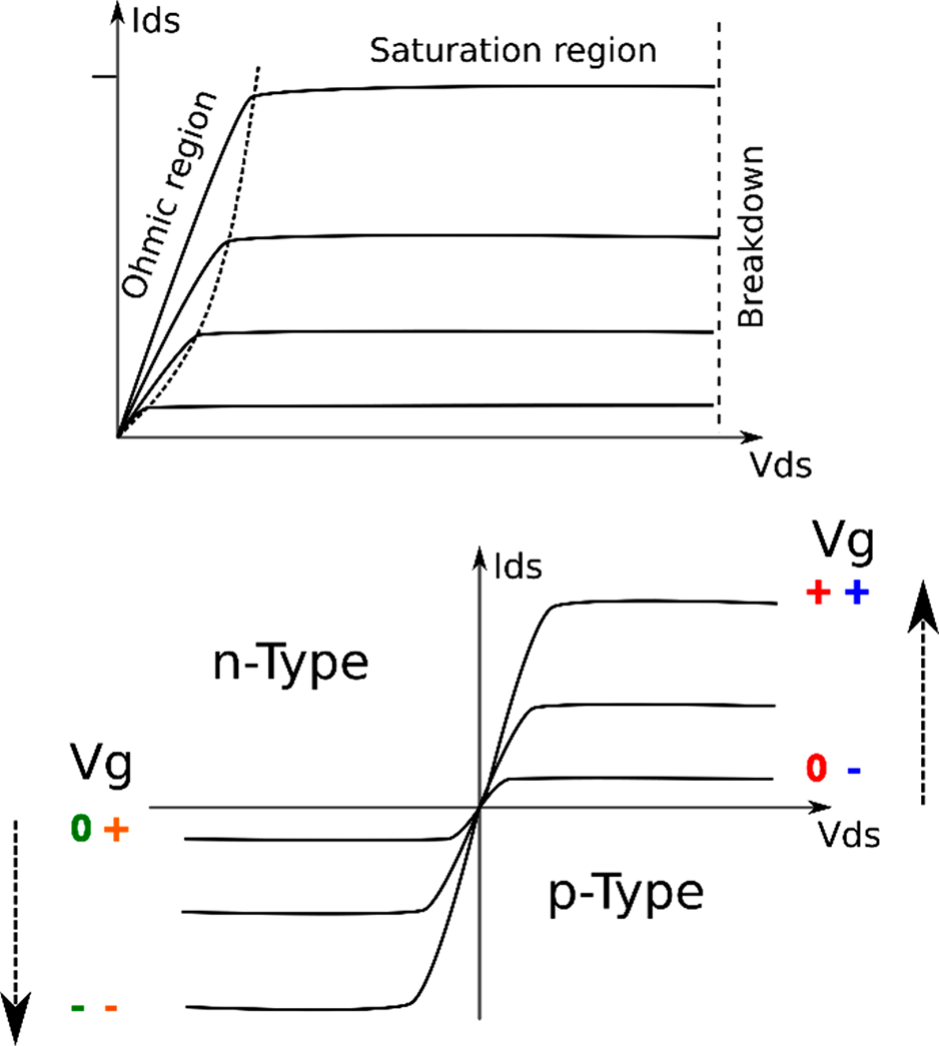
An example series of output curves illustrating the different regions of operations in a device (top) and a series of curves showing how the output curve will vary with gate voltage sign in different types of devices, by type and operating mode (bottom). Green and red show the sign of the current in enhancement mode devices, while orange and blue show it in depletion. Both p-type and n-type operation are displayed for convenience. Typically, only one of the two types of operation is present in a device.

Another important characteristic is the output curve. It displays the variation in drain current with *V*_sd_. Typically, several curves are plotted for different values of *V*_g_ (Figure 4). The output characteristic of a transistor is typically a plateau-like curve, with three distinct regions. The first is an initial rise, known as the Ohmic region, where *I*_d_ has an approximately linear increase with *V*_sd_. At a given voltage, known as the pinch-off voltage (*V*_p_), the curve is flattened, giving rise to the saturation mode. The slope of the curve in the saturation mode gives the output impedance of the device. Beyond that is a breakdown region for the device— in classical FET devices, this corresponds to breakdown of the dielectric, and ohmic conduction across the semiconductor interface, while in devices such as OECTs, this typically occurs at the edge of the electrochemical window.

Frequently, the output curve contains a loading line—which shows the current–voltage relationship of an ohmic load on the transistor, where this ohmic load can be used as an abstract representation for an electrical component that is powered by the transistor (e.g., the next stage in an amplification system). This depiction allows a circuit designer to determine the voltage range in which the device will faithfully amplify (i.e., in a linear, distortion-free manner) a signal voltage applied at the gate and is important for circuits acting as voltage amplifiers.

##### 3.2.2.3 AC Analysis

AC characteristics of transistors can be acquired in several ways. First, analogous experiments to EIS can be carried out, where a sinusoidal signal of varying frequency is applied to the gate of the device and transconductance is recorded as a function of frequency. Additionally, transient responses of the device, such as that to a square voltage pulse at the gate, can give performance metrics in the form of time constants of various responses.

The AC response of a transistor can typically reveal the gate capacitance of the device, which is a strongly material-dependent property. However, it is worth noting that at higher frequencies of investigation, other factors, such as various parasitic capacitances between the different terminals, become increasingly relevant.

#### 3.2.3 Device Engineering of OECTs

As with any device, in order to effectively design OECTs, reliable physical models of their behavior are needed. Device physics of OECTs is a very active field, which has been recently reviewed.^32^

**Figure 5:**
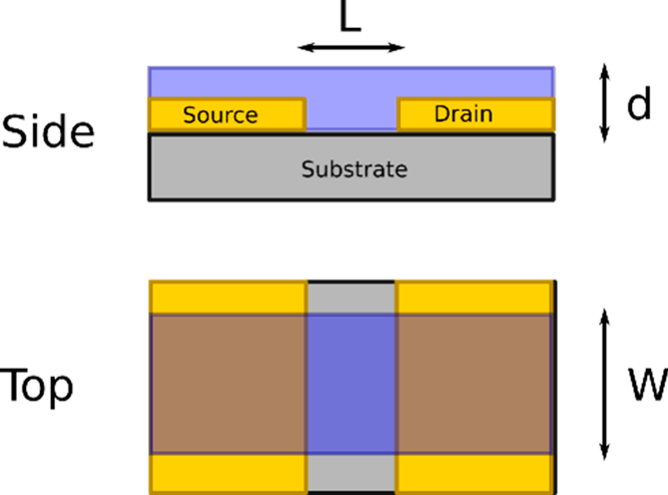
Schematic showing the top and side on structure of a typical OECT.

First, any discussion of device engineering and modeling makes frequent reference to the channel dimensions, which are the length (the distance between the source and drain), the width (the l dimension of the channel parallel to the contacts), and *d*, the film thickness of the deposited channel material, as shown in Figure 5.

The majority of OECT device modeling has risen by way of comparison with traditional MOSFET (metal-oxide semiconductor FET) devices. In common between both devices is the presence of a capacitive element at the gate. In a FET, this is a layer of dielectric and is responsible for the buildup of charge carriers in the channel, and as with any conventional capacitor, its capacitance increases with the gate-channel-overlap area. While such capacitive elements also exist in OECTs, there are two capacitances to consider in that case: The capacitance at the gate electrode–electrolyte interface and the capacitance at the channel. The latter is a function of the volume of the entire channel, due to the volumetric capacitance displayed by OECT materials—ions can penetrate the bulk of the channel, effectively increasing their contact area with the electronically conductive parts of the material.

**Figure 6:**
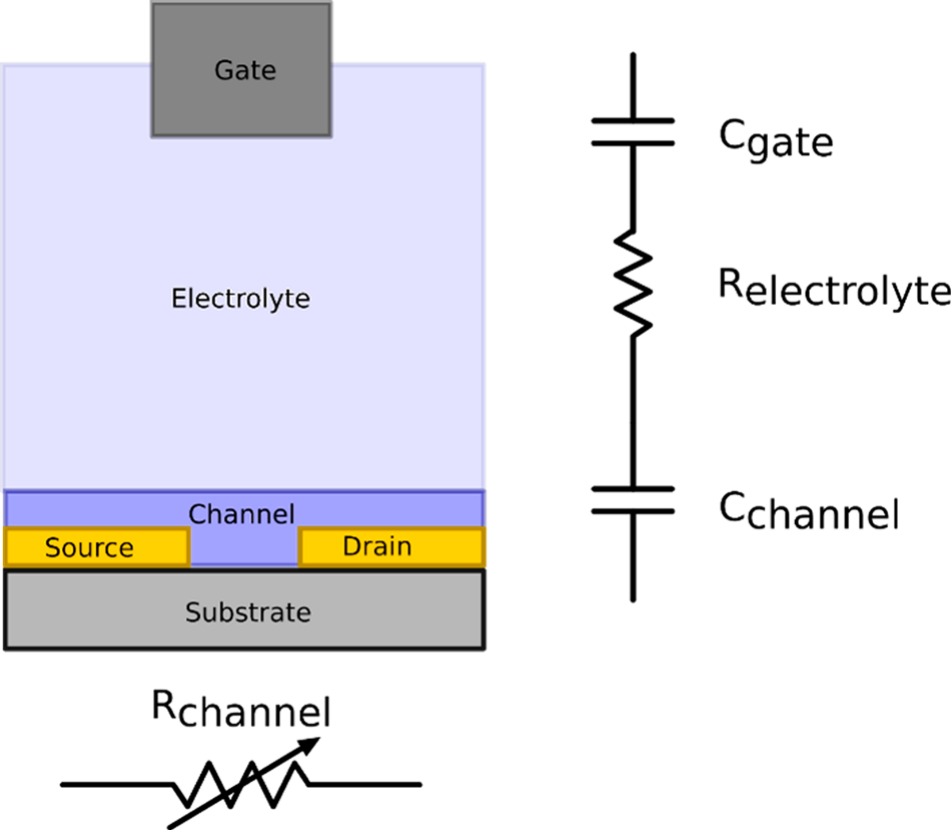
Simplified representation of an OECT in terms of conventional passive components.

The majority of theoretical OECT descriptions are based on the Bernards’ model,^33^ in which the OECT is compartmentalized in an electronic and ionic component. The electronic component models the ohmic behavior in the channel, while the ionic component is represented by a resistor and capacitors in series (Figure 6). As discussed above, the capacitors account for the interfacial capacitance of the gate electrode and the capacitance of the channel, while the resistor incorporates the electrolyte ohmic drop. From basic electronic considerations, it follows that for the channel capacitance to dominate the response, the capacitance of the gate electrode must significantly exceed that of the channel (otherwise, the bulk of the *V*_gs_ would drop across the gate electrode–electrolyte interface and not across the channel). As such, large electrode areas are used for metallic electrodes, potentially replacing them with PEDOT:PSS electrodes (due to their higher volumetric capacitance, C*, defining the capacitance per unit volume of the material), or with nonpolarizable electrodes such as Ag/AgCl, so as to minimize the contribution of the gate–electrolyte interface. Finally, Bernard’s model yields a useful expression for the steady-state transconductance:
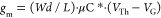
where μ is the electronic charge carrier mobility and C* is the volumetric capacitance.

While the initial version of Bernards’ model represented the channel as a planar capacitor, improvements in understanding of mixed conductors have led to the use of a volumetric capacitance as a representation for the channel capacitance, in later works.^34^ Additional refinements of the model have incorporated doping-dependent mobility of the charge carriers^35^ and extended the description to negative *V*_gs_ and, thus, better predicted the maximum transconductance of a device.^36^ Additionally, it is worth pointing out that Bernards’ model and its improvements aim at providing a satisfying engineering abstraction of the physical device. Recently, models have been developed that accurately predict many experimental observations related to charging, such as the recent work of Tybrandt et al.^37^ In that model, the chemical potential of the holes in the conductive polymer is coupled to the electrical double layer, formed at the interface between the semiconductor (PEDOT) and the ionic phase (PSS), which is a more accurate representation than a continuous volume assumption for the channel.

The aforementioned architecture, with an electrode immersed in an electrolyte acting as a gate, has also undergone evolution. While it is a very convenient setup for analytical chemistry applications, the lack of independently addressable gates makes certain kinds of common circuitry difficult to engineer, especially if a small size is also desired. The recently developed internally gated architecture, which incorporates pockets containing ions within the channel and an electron-insulating, ion-conducting barrier, allows separate gating of multiple devices, along with significantly improved operating frequencies.^30^

For completeness, it is important to note that OECTs represent an extreme case along a continuum of device behaviors, with the opposite extreme defined by another device, the EGOFET—electrolyte-gated organic field-effect transistor.^38^ In those devices, the ions in the electrolyte do not permeate the film and instead accumulate on the channel interface, giving rise to a field effect in the channel. Some materials display an in-between behavior, where ions indeed penetrate the channel but with a significant activation barrier.

A final parameter to consider, where there is interplay between device engineering and dimensions and chemical composition, is the so-called parasitic series resistance, which can significantly influence device performance and confound materials characterization efforts. This effect can be especially prominent in high-transconductance devices.^39^ While its origin can often be traced to purely device-architecture factors, such as interconnect dimensions or overlap between the injecting metal electrode and polymer channel, the chemistry of the materials has been shown to have a significant effect on device n-type OECT characteristics.^40−42^ In general, for the successful operation of any transistor, based on an organic channel, a good energetic match of the workfunction of the metal contact and the LUMO of the semiconductor is required for n-type operation (and conversely, the HOMO of the semiconductor for p-type operation). When these energy levels are close, there is a negligible energetic barrier to be overcome, when electron transfer occurs between the metal contact and the semiconductor (i.e., an Ohmic contact), and thus lower channel resistance leading to higher on currents and gain.

These electrochemical requirements have led to the prominence of gold as an electrode in most p-type organic transistors, due to its energetic suitability (certain n-type polymers, with favorable LUMO energies, can also be used with gold electrodes,^43^ but generally calcium contacts are more suitable). However, room for quantitative improvements in energetic match still exists, leading to various chemical functionalization treatments for electrodes—for example, the use of thiolated SAMs on gold electrodes.^44^ While extensive work in such contact-engineering problems has been carried out for OFETs,^45,46^ comparatively little work on contact engineering has been done with OECTs.

Additionally, difficulties associated with depositing gold (typically involving high-temperature, high-vacuum processes, such as thermal or electron beam evaporation) make its use on stretchable substrates or low-cost, high-volume applications (e.g., for disposable wearable electrodes, as could be used in EEG applications) difficult. Replacing gold with lower-cost materials, such as inkjet-printed carbon or silver, that can be deposited at close-to ambient conditions could greatly expand the use of OECTs in high-volume applications.^47^

#### 3.2.4 Benchmarking OECT Materials

From the above discussions, it is clear that multiple figures of merit (FOMs) can be derived from the transistor characteristics. Historically, several different figures have been used to compare OECTs and the materials constructing them, since the increase in interest toward them in the past decade. Initially, devices were compared by reporting dimensionally normalized transconductances. These metrics, however, are much more suitable in comparing individual devices and architectures, with a view toward potential applications. Comparing devices with the aim of materials optimization would require FOMs that are geometry and architecture independent, and this has been recently noted,^28^ leading to the widespread adoption of the μC* product as a materials-based figure of merit. This trend of changing the figures of merit makes historically benchmarking materials difficult.

## 4 Material Properties of Semiconducting Polymers and Their Control

### 4.1 PEDOT:PSS: An Established Neuroelectrode Material

While several polymers have been studied for neuroelectronic applications, recent literature is dominated by PEDOT:PSS. PEDOT:PSS is a polymeric blend of two materials—polyethylenedioxythiophene (PEDOT), an organic semiconductor, and polystyrenesulfonate (PSS), a polyelectrolyte. The blend is biphasic, with a PEDOT-rich and a PSS-rich phase.^48−50^ Ions from an electrolyte can effectively percolate through the PEDOT:PSS film where they are transported in the PSS-rich phase. This allows for an effectively larger contact area between the electrolyte and the electrode.^50^ This is the basis for a phenomenon known as volumetric capacitance, which has been exploited in the construction of neural recording microelectrodes.^51^ This has allowed for very large ESA/GSA (electrochemical surface area/geometric surface area) ratios, enabling new experiments—such as the recording of single-unit activity using an array of cortical surface electrodes (as opposed to traditional single-unit recordings, which use penetrating electrodes such as wires and needles).^51^ This has been attributed to the lowered impedance of the electrodes. The impedance of a thin-film electrode made of a mixed conductor, such as PEDOT:PSS, can be shown to depend on the volumetric capacitance C* of the material, along with the solution resistivity, and the dimensions of the electrode.^23^ Typically, values for C* for PEDOT:PSS are reported in the range of 40 F cm^–3^.

The mixed conductivity of PEDOT:PSS is exploited in the construction of OECTs. In those devices, in addition to C*, which dictates the ionic response, another crucial parameter is the charge carrier mobility, μ, which dictates the electronic conductivity. μ depends on the chemical structure of the polymer backbone, the packing of polymer chains, and parameters such as the relative sizes of the PEDOT- and PSS-rich domains. As such, postprocessing of samples and additives during deposition can change the measured conductivity of a PEDOT:PSS sample by 2 or 3 orders of magnitude. Commonly reported additives for PEDOT:PSS films are high-boiling solvents and surfactants.^49,52,53^ Recently, molecular and polymeric additives containing nitrogen atoms with available lone pairs, that can alter the charge carriers, have been employed to change the behavior of devices from depletion mode to accumulation mode.^29,54,55^

Finally, use of sorbitol as an additive has also recently been demonstrated, with evidence that it modifies the structure of PEDOT:PSS films, so that the film incorporates internal ion-filled reservoirs.^29,30^ This leads to a significantly faster response time, giving bandwidths of hundreds of kHz.

### 4.2 Improving Materials for Neural Interfacing

PEDOT:PSS has been established as a material for neuroelectronic interfacing with significant improvement over conventional metal electrodes in many ways and applications. However, the demands of bioelectronics in general, and neural interfacing in particular, form a driver for further improvements in electrode properties, whether by modification of PEDOT:PSS or by the introduction of potential successor materials. There is scope for further reducing electrode impedance, increasing transconductance, and improving power consumption. Furthermore, devices operating at higher frequencies can enable more sophisticated neural electronics, such as those capable of some limited signal processing and conditioning.

#### 4.2.1 Volumetric Capacitance

**Figure 7:**
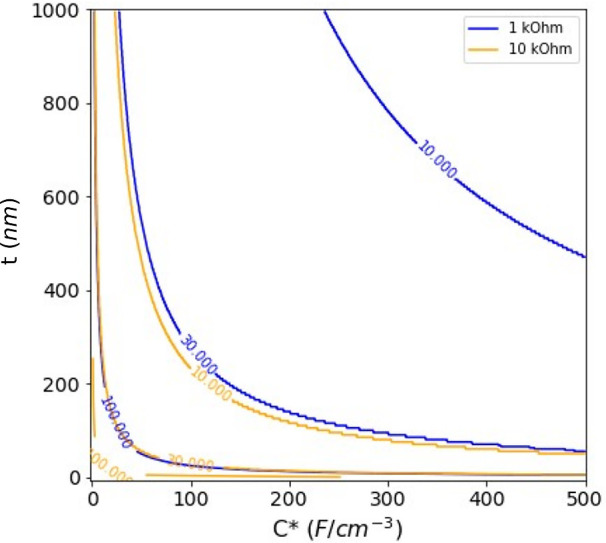
Isoimpedance lines for a given volumetric capacitance, C*, thickness, t for a square electrode of a given side (10, 30, or 100 μm).

As mentioned above, current devices made of PEDOT:PSS typically report volumetric capacitances of approximately 40 F cm^–3^. Using the equations describing the behavior of gold bioelectrodes with thin PEDOT coatings from Koutsouras et al.,^23^ we can predict the minimum dimensions of an electrode, with a given C*, that would have a hypothetical target impedance (Figure 7). As an example, to achieve an impedance of 1 kΩ at 1 kHz (i.e., a very low impedance, in a frequency range crucially important to single-unit recording) for a square electrode with a 10 μm side, an order of magnitude increase in C*, to approximately 400 F cm^–3^, should be aimed for.

#### 4.2.2 Charge Carrier Mobility and Identity

In addition to being an ionic conductor, PSS also acts as a dopant (an acceptor)—the sulfonate groups on the PSS compensate holes on the PEDOT chains.

Due to the involvement of both an ionic and electronic circuit, OECT behavior is dependent on both C* and μ.^32^ Their product governs behavior such as the maximum transconductance and, individually, the response time scale (i.e., the time scale associated with changes in *I*_d_ when *V*_g_ changes). Generally, two time constants are important in the temporal response—the electronic one (depending on μ) and the ionic one (depending on C*).^32^ Which factor dominates determines whether the transistor will have a predominantly slow response, a predominantly ionic response, or a fast, predominantly electronic one, similar to recently reported sorbitol-enhanced OECTs.^29,30^

**Figure 8:**
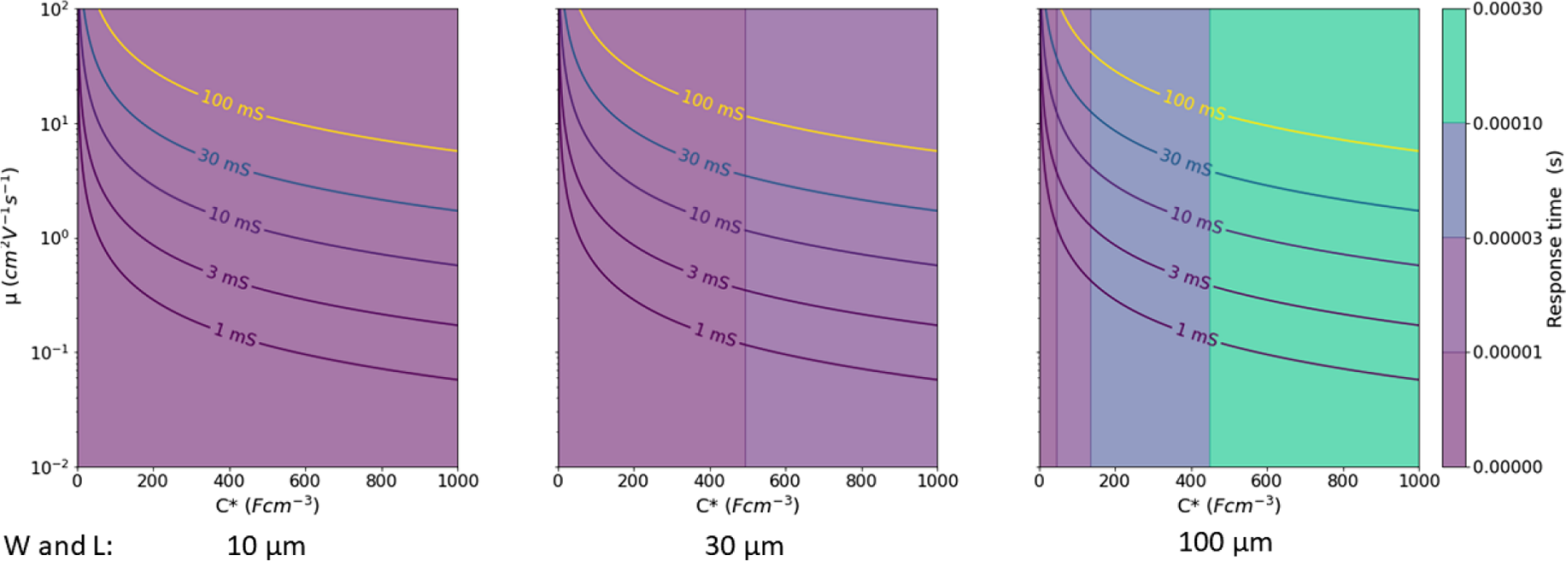
Plots showing the transconductance and response time for transistors of various dimensions, made of materials with values of μ and C* indicated on each plot. Resistive contribution is calculated according to data given in ref (23): Ω, *V*_g_ = 0, *V*_t_ = −0.5, *t* = 350 nm.

Using previously reported equations and data,^23,32^ for the dependence of the response time and transconductance, based on μ and C*, and making several assumptions about device parameters, one can draw a series of plots showing the behavior of devices of various dimensions. These illustrate an important reality about OECT operation—high transconductance often comes at the cost of slow operation. This is a consequence of basic electrochemistry, due to the increased overall capacitance of the device. This trend is illustrated in Figure 8. Increasing the volume and transconductance makes devices slower, necessitating higher and higher μ, to ensure satisfactory response time and transconductance.

PEDOT:PSS is typically a p-type conductor, which has fundamental consequences on what types of devices are easy, or even possible, to engineer, using the material. Also, a PEDOT:PSS channel is normally doped in the absence of an applied gate voltage and switches off when a positive gate voltage is applied in what is known as depletion mode behavior. Modifications have been carried out, using chemical additives, to lower intrinsic off current and achieve p-type accumulation mode behavior, i.e., normally off, switching on upon biasing the gate. In general, however, a full complement of organic materials, displaying all permutations of p- and n-type, depletion and accumulation mode behavior would be desired for maximum flexibility in circuit design.

It is important to point out considerations about charge carrier and accumulation vs depletion behavior in an OECT are an important increase in complexity over those when selecting materials for a passive electrode. For example, high conductivity is a necessity in a passive electrode, while that may not be the case in complex circuits incorporating OECTs.

#### 4.2.3 Biocompatibility

PEDOT:PSS and several derivatives and analogues have shown good biocompatibility in multiple studies.^56,57^ PEDOT:PSS has demonstrated compatibility with neural cells and an ability to attenuate glial proliferation *in vitro* in several studies.^58^ This is a promising result, as the foreign-body reaction to an implant in the brain leads to encapsulation in a glial scar, which is detrimental to the electrical performance of an implanted device. Additionally, the organic chemical nature and comparatively mild deposition conditions of PEDOT:PSS would also lend it to functionalization with various biomolecules.^59^ Such biomolecules could also be used to replace PSS as a dopant, potentially improving biocompatibility, without compromising conductivity.^60^ Additionally, PEDOT:PSS can also be used in drug-eluting studies, where a molecule that can suppress the foreign-body reaction is released, such as dexamethasone or derivatives thereof.^61−64^

This ability to elute compounds naturally leads to an important consideration regarding the biocompatibility of PEDOT:PSS and other conjugated polymers—namely the potential to leach out any small molecule contaminants. In general, this might be PEDOT or PSS oligomers, or any small molecule additives, such as surfactants. In the context of PEDOT-containing polymers (and those derived of structurally similar monomers), it is important to note that Ames and comet testing has shown that EDOT may potentially exhibit some toxicity.^65^ Additionally, an important consideration is any impurities in the final film, such as any residual transition metal catalysts. Care should also be taken when applying a voltage at an electrode, as this could iontophoretically displace charged species into the tissue.

A commonly cited advantage of conductive polymers, in the context of biocompatibility, is increased mechanical deformability. While softer compared to many metallic electrodes, PEDOT:PSS is still in the low-GPa, high-MPa range of mechanical moduli, which is 2 to 3 orders of magnitude stiffer than brain tissue.^66^

Practically, assessing biocompatibility can be done by standardized testing, for various applications (e.g., for cutaneous electrodes versus implanted ones). A general starting point for demonstrating biocompatibility can be the ISO-10993 standard.

#### 4.2.4 Mechanical Deformability

While many ways exist for engineering stretchability and deformability in electronic devices with metal electrodes and interconnects (e.g., by incorporating serpentine or fractal curves or by predeforming substrates during deposition),^67,68^ the intrinsic deformability of organic electronic materials remains a desirable advantage. However, despite the relatively lower mechanical modulus for organic materials, it is still high compared to most neural tissue—PEDOT:PSS has been reported in the range of several GPa,^69^ while brain tissue is in the several hundred Pa to low kPa range^70^—typical metals used for electrodes, such as platinum or titanium, range in the hundreds of GPa. As such, conductive polymers with mechanical properties similar to PEDOT:PSS tend to be sufficiently soft to reduce trauma, due to an implant’s micromotion in the brain,^71^ but are still stiffer than the surrounding tissue, potentially triggering a foreign-body reaction.^66^

The mechanical properties of conductive polymers are a consequence of their typically long, rigid, conjugated polymer backbones, themselves dictated by electronic requirements. Attempting to reconcile the mechanical and electronic properties of organic electronic materials, by use of additives or by using chemically novel entities, is a very active area of research.^72,73^ Similarly, formulating conductive polymers as hydrogels has also been reported but is outside of the scope of this review, as that would require a review of hydrogel chemistry in parallel—we direct the reader to a selection of excellent reviews.^74−76^ Additionally, PEDOT:PSS can also be blended with elastomers, as an alternative to hydrogel approaches.^77^

Lastly, in characterizing mechanical properties, it is important to point out the large variety of techniques available, that reflect the different scale and mechanical stress applied to the sample. In typical applications, semiconducting polymers are deposited as thin films and often upon a thicker, and often stiffer, substrate that tends to dominate the mechanical response. Atomic force microscopy (AFM) has been used for characterizing the mechanical properties of polymer-coated bioelectrodes.^78^ AFM brings the additional advantage that it is capable of sensing the influence of an underlying stiffer substrate, which would be beneficial in very thin and soft coatings.^79^ Other suitable methods have been reviewed by Root et al.^73^

## 5 Types of Neural Applications

The field of semiconducting polymers in neural applications is very broad, which allows for it to be categorized in many ways. In this review we discuss applications in electrophysiology. Those can be subdivided into *in vitro* and *in vivo* applications, with a further subdivision of invasive and noninvasive *in vivo* applications. In this review, we will focus on *in vivo* applications. Electrophysiology applications can be further subdivided based on whether the devices record or stimulate neural activity. Additional applications, beyond the scope of this review, include uses in tissue engineering, drug delivery, and optically active devices.

### 5.1 Noninvasive Applications

Noninvasive neural applications are constrained to techniques such as electroencephalography (EEG) and magnetoencephalography (MEG) when recording signals. There are also other cutaneous electrophysiological recording applications, where organic conductors are used—notably electrocardiography (ECG) and electromyography (EMG), which however do not record neural signals and are thus outside the scope of this review. There are also established stimulation-based applications—transcutaneous nerve stimulation (TENS) and transcranial electrical stimulation (tES).

#### 5.1.1 Electroencephalography

Electroencephalography (EEG) is the use of electrodes placed on the scalp to record the voltages generated on the skull that are caused by the summation of the electrical activities of neurons in the brain. Typically, these signals can range from the tens of Hz range up to mV in magnitude. The signals of interest can be classified based on their spectral bands, with the most studied being alpha and gamma (frequently designated as the 8–15 Hz and >32 Hz ranges). Electrodes are typically large (length scales of several centimeters are common), due to low signal amplitude—as such lowering impedance in contact with skin (and frequently in the presence of hair) is highly desirable. In general, this application is less demanding in terms of biocompatibility, being external to the body. Improvements in the adhesive properties of the recording electrode (or transistor channel) could improve recording quality, by reducing the potential for mechanical artifacts. Additionally, due to the wearable character of EEG, polymers that can withstand mechanical deformation are also sought after.

Organic electronic polymers have seen frequent use, in efforts to satisfy these requirements. A recent report demonstrated a PEDOT-based electrode that is cured, by illumination, directly onto a patient’s scalp and was used to demonstrate EEG recordings on freely moving animals.^80^ Additionally, electrochemical PEDOT:PSS deposition has allowed the fabrication of conducting strings, with the potential to easily incorporate EEG into wearables.^81^ PEDOT:PSS dry microneedle electrodes have also been shown to reduce skin impedance compared to conventional wet electrodes.^82^ Fast-responding, small footprint OECTs, with an adhesive surface, have been demonstrated to successfully record signals between individual hair follicles.^30^ Other polymers have also been trialled for use in improving EEG recordings, with examples of passive polyanilline electrodes,^83^ and in a demonstration of how novel polymers enable novel device designs, a subthreshold transistor amplifier for EEG, using p(g2T-TT).^84^ Finally, PEDOT:PSS electrodes show better compatibility with magnetic fields than their metallic counterparts, making them suitable, for example, in situations where EEG is used simultaneously with MEG.^85^

### 5.2 Invasive

Invasive electrophysiological applications consist of implantable technologies. Typically, those can be subcutaneous EEG and tES, electrocorticography (ECoG), and penetratingelectrode interfaces. Similarly, interfacing with various elements of the peripheral nervous system (i.e., nerves outside the brain and spinal cord) has been done in analogous preparations. Invasive applications can be acute or chronic, depending on the time scale of the device implantation. In current clinical practice, stimulation-only devices tend to predominate on chronic time scales. Stimulators for various applications exist—for pain management, for motor disorders, for prosthetics actuation, or, recently, for electroceutical applications (i.e., stimulation of nerves responsible for autonomic control of organs and systems, such as the vagus nerve). Devices that record electrophysiological data chronically tend to be confined to a research environment. This is in part due to the long-term instability of the water–electrolyte interface; corrosion tends to compromise the electrical contact, which increases impedance. This issue is also exacerbated by the presence of a foreign-body reaction,^86^ which encapsulates and thus insulates the implant, in addition to contributing to electrochemical corrosion via reactive oxygen species.^87^ While, in applications where stimulation is only required, degradation of the interface can be partially accommodated by adjusting stimulation parameters, this is not possible in recording applications, where the initial signal is biologically determined and then degrades significantly.^88^ Attempts to rectify this, and thus enable chronically recording implants, have been one of the key drivers for the search for novel electrode materials, such as organic electronic polymers.

#### 5.2.1 Electrocorticography

Electrocorticography (ECoG) is a similar idea to EEG, although in this case, devices are placed underneath the skull, on the surface of the cerebral cortex. An electrode array is placed on the cortex and can be used to record a variety of signals. Biocompatibility requirements are more demanding than for noninvasive applications, due to the implanted character, as are the demands on the material, due to attack on the device by the immune system. Mechanical flexibility is also a beneficial trait for a device, as it would promote conformal contact with the brain surface.

Generally, signals such as LFPs (localized field potentials–consisting of the spatiotemporal summation of the activity of dozens to several hundreds of neurons) are recorded, which are in the hundreds of Hz range. However, in a very illustrative example of successful uses of organic conductors as neural electrode coatings, the use of PEDOT:PSS coatings on gold microelectrodes lead to an order of magnitude decrease in impedance at 1 kHz.^51^ This decrease was sufficient to allow the detection of single-units (i.e., voltage spikes caused by the depolarization of a single neuron) with a planar electrode. This type of signal typically requires a low impedance at 1 kHz, as the duration of the individual voltage spikes is around 1 ms. Similarly, the decrease in individual electrode impedance by PEDOT:PSS coating has also been used to increase available channel counts.^89^ PEDOT:PSS compatibility with organic materials has also enabled the realization of polymer arrays with hydrogel substrates, allowing for better adhesion and conformity to the brain surface, with additional advantages in MRI compatibility, by forgoing metal electrodes.^90^

#### 5.2.2 Peripheral Nerve Interfacing

Peripheral nerve interfacing, whether stimulation only or bidirectional, comprises a significant portion of clinical electrophysiological applications. Several possible architectures exist for peripheral nerve interfaces, incorporating surface or penetrating electrodes. Generally, the challenges in this environment derive from the combination of an electrochemically, biologically, and mechanically challenging environment (e.g., for implants attached to motor nerves, or generally in areas of the body which undergo significant movement).

Various stimulators have been in the clinic for decades, for various motor difficulties and for control of neuroprosthetics.^91,92^ Recently this has been expanded to so-called electroceuticals, where various autonomic nerves are stimulated.^93^ In light of these stimulation-focused applications, conducting polymers find application due to their increase in charge injection capacity, and thus stimulation effectiveness, with additional benefits of mechanical conformability.^94^

#### 5.2.3 Penetrating Electrode Recordings

Penetrating electrodes consist of micron-scale probes that can be used for recording LFPs or single-units. Generally, they provide the highest spatial resolution and enable probing of subsurface structures in the brain (i.e., below the cortex), at the cost of increased invasiveness and foreign-body reaction. Demands on the electrical properties in such implants are also greatest, due to the typically small device size, constrained by the footprint of the implant needle.

## 6 Organic Mixed Ionic-Electronic Conductor Classes

**Figure 9:**
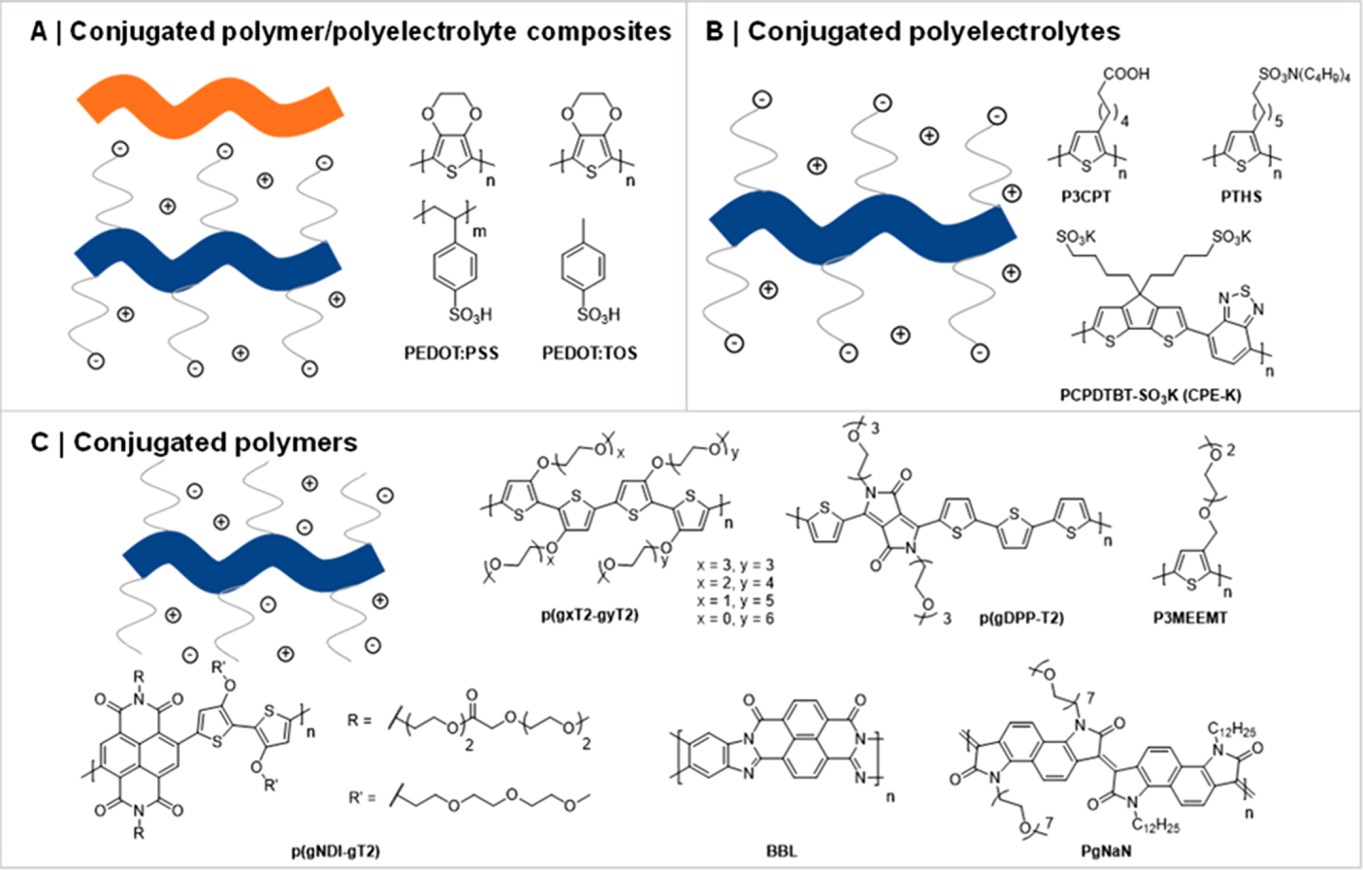
Different classes of OMIECs, including conceptual sketches highlighting the defining characteristics for each OMIEC class. Broad ribbons correspond to polymer backbones, while narrow ribbons to pendant side chains. Blue and orange denote material sections responsible for electronic and ionic charge carrier transport, respectively. Cations and anions are represented by their respective charge symbols in a circle.

Broadly speaking, OMIECs can be classified into three main categories: (i) conjugated polymer/polyelectrolyte composites, (ii) conjugated polyelectrolytes, and (iii) conjugated polymers; see Figure 9. The key distinguishing feature across these material classes is whether they make use of a single component responsible for both ionic and electronic charge carrier transport (conjugated polyelectrolytes and conjugated polymers) or whether they employ multiple components to segregate ionic from electronic charge carrier transport (conjugated polymer/polyelectrolyte composites). Moreover, a further division can be made depending on whether the material under study features a covalently attached ionic moiety, which can be compensated by a counterbalancing ion or an electronic charge carrier on the conjugated polymer backbone.

### 6.1 Conjugated Polymer/Polyelectrolyte Composites

**Figure 10:**
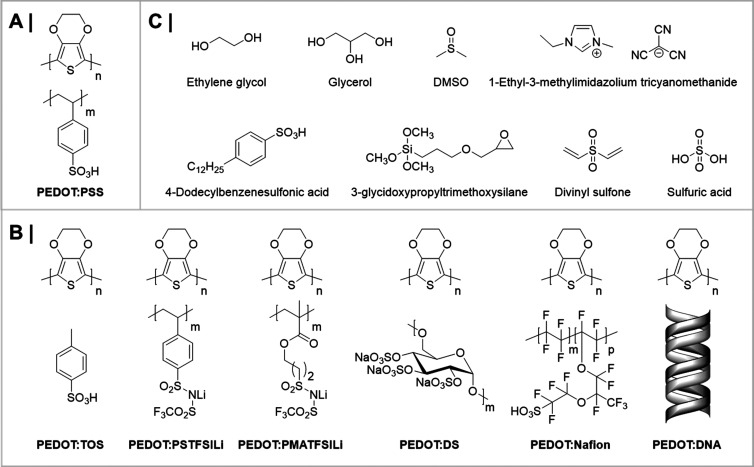
(A) Chemical structure of PEDOT:PSS. (B) Chemical structures of conjugated polymer/polyelectrolyte composites derived from PEDOT:PSS. (C) Frequently employed molecular additives to improve the performance, stability, or processability of PEDOT:polyelectrolyte-based composites.

To date, the most widely explored and well-studied conjugated polymer/polyelectrolyte composites for bioelectronic applications are those based on the conjugated poly(3,4-ethylenedioxythiophene) (PEDOT) backbone, first developed by Bayer AG in the 1980s (Figure 10).^95−97^ In the absence of a charge balancing polyelectrolyte component, PEDOT films possess several desirable features for organic electronic and bioelectronic applications, including high electrical conductivities (>300 S cm^–1^) and high optical transparencies across the visible light spectrum yet are virtually insoluble, thus preventing facile solution processing.^98^ In these materials, solution processability is typically imparted by inclusion of a charge balancing polyelectrolyte, most commonly poly(styrene sulfonic acid) (PSS), thereby leading to an aqueous PEDOT:PSS dispersion with good film forming properties, moderate electrical conductivity (0.1–1 S cm^–1^), high mechanical flexibility, and robust thermal and electrochemical stabilities.^95−97,99^ These favorable characteristics of PEDOT:PSS, in addition to its widespread commercial availability (e.g. Clevios PH1000, Baytron P, and Orgacon), have promoted its adoption in virtually every area of organic electronics, including as anodes or hole injection layers in organic light-emitting diodes,^100−102^ anodes, or hole transport layers in organic photovoltaics,^49,103−105^ optical elements in electrochromic displays,^98,106^ p-type legs in thermoelectric generators,^107−109^ active matrix displays,^110^ electrode materials for supercapacitors,^111^ and many more.

#### 6.1.1 Synthesis of Composites

Synthetically, PEDOT-based composites can be obtained through three primary polymerization methods: electrochemical, chemical oxidative, and chemical vapor phase polymerization (VPP). In each polymerization technique reaction conditions can be tuned to modify the final properties of the polymers. Electrochemical polymerization proceeds through the electrochemical oxidation of PEDOT’s 3,4-ethylenedioxythiophene (EDOT) monomer, dissolved in a solvent and in the presence of an electrolyte. The properties of the final polymer can easily be tuned by changing the employed deposition method (potentiostatic, galvanostatic, pulsed, or cyclic voltammetry), applied potential, amount of passed charge, and scanning speed.^112−115^ Similarly, monomer concentration, electrolyte concentration and nature, and solvent choice can also have drastic impacts on the resulting electrochemical and morphological properties of the polymers.^106,112,116^ In this context typical solvents include acetonitrile, propylene carbonate, and water, while common electrolytes include lithium perchlorate, tetrabutylammonium perchlorate, and tetrabutylammonium hexafluorophosphate. Alternatively to using PSS as polyanions,^117^ the use of biologically active dopants such as biopolymers to further promote the compatibility between the PEDOT composite and the investigated biological specimen has also attracted considerable attention. Prominent examples of biopolymer counterions include glycosaminoglycans (e.g. heparin, hyaluronic acid, etc.), nerve growth factors, and polysaccharides, among many others.^57,118−120^ Early demonstrations of PEDOT:PSS-coated neuroelectrodes were also carried out via electrodeposition.^121−123^ Electrodeposition of PEDOT has even been performed in living tissue.^124,125^ Additionally, PEDOT has been deposited in a variety of supporting matrices,^126,127^ membrane materials such as Nafion,^128^ carbon nanotubes,^127^ and metal nanoparticles.^64^ Crown ether functionalization for Na^+^ and K^+^ ion sensing has also been realized by this method.^129,130^ Historically, the use of electrodeposition has been particularly important in the fabrication of implantable neural probe electrodes.^131−133^ PEDOT derivatives, such as PEDOT-MeOH/PSS, have also been electropolymerized for use in implantable neural probes.^121^ In addition to commonly investigated silicon and gold substrates, PEDOT coatings on Pt, IrOx, and Mg have also been reported.^134,135^

Despite the vast synthetic flexibility of the electrochemical polymerization method, its primary drawback is the necessity for a conductive surface for the polymerization process to occur, thus severely limiting substrate choice and rendering it unsuitable for large-scale polymer synthesis. However, electrodeposition, nonetheless, can be successfully used to bridge small areas of insulating material, such as the gap between an OECT source and drain electrode.^136^ Furthermore, the incorporation of bulky biopolymers into PEDOT-based films during electrochemical polymerization has also been shown to be detrimental toward the films’ electrical and mechanical properties.^118,137,138^ Chemical polymerization methods thus tend to be preferred, which typically involve the use of the EDOT monomer, an iron(III) oxidant, and a solvent. Common iron salts used are iron(III) chloride, iron(III) perchlorate, and iron(III) *p*-toluenesulfonate, while sodium and ammonium persulfate have also been employed as alternative oxidants. Variation in either of these three synthetic handles, as well as their concentration, reaction time, temperature, etc., can be employed to tune the resulting material properties. Importantly, the electrical and mechanical properties of PEDOT-based composites synthesized through oxidative chemical polymerization are on par as those synthesized through electrochemical polymerization means,^98,139^ while biopolymer incorporation is also possible with this polymerization technique.^60,140,141^ Moreover, the final polymers’ properties can be adjusted through postdeposition processing, thus enabling further polymer property optimization following their synthesis.

Finally, vapor phase polymerization is another established technique to incur PEDOT composites and is closely related to the chemical polymerization method. In this case, however, the EDOT monomer is introduced in vapor form to an oxidant-coated surface, thus leading to polymerization at the vapor–oxidant interface.^142,143^ A subcategory of vapor phase polymerization is chemical vapor deposition in which both the oxidizing agent and EDOT monomer are introduced as vapors, thus foregoing the need to deposit the oxidizing agent on the desired substrate prior to polymerization. Similarly to chemical polymerization, vapor phase polymerization also typically makes use of iron(III)-based oxidants,^144,145^ although alternative oxidants such as bromine,^146^ copper(II) chloride,^147^ and chloroauric acid^148^ have also been employed. A typical issue encountered during VPP is an excessively high reactivity of the employed oxidant, leading to uncontrolled polymerization kinetics and in turn the formation of polymers with short conjugation lengths and several defects along the conjugated polymer backbones that negatively impact the polymers’ electrical properties.^149^ Therefore, considerable focus has been placed on tailoring the oxidant’s reactivity, with two main solutions having been developed. The first involves the use of liquid alkaline inhibitors with relatively high vapor pressures such as pyridine or imidazole to regulate the pH in the oxidant film.^139,144^ Here the base is thought to inhibit any undesirable acid catalyzed side reactions by adjusting the pH in the oxidant thin film. The second method utilizes glycol-based additives in which the glycol-based species are thought to reduce the apparent reactivity of the oxidant by formation of a complex.^150,151^ Overall, VPP has established itself as a suitable technique to afford PEDOT-based conjugated polyelectrolyte blends with excellent electronic properties, with electrical conductivities as high as 8800 S cm^–1^ having been reported.^152^ Nonetheless, the use of VPP to afford high-quality OECT channels has been limited,^28,153,154^ in particular due to the widespread commercial availability of PEDOT:PSS dispersions synthesized through solution chemical oxidation processes, which only require deposition and postprocessing treatments to incur semiconductor films with similar properties that can be achieved by VPP.

#### 6.1.2 Additives and Postdeposition Treatments of Composites

##### 6.1.2.1 Secondary Dopants

**Figure 11:**
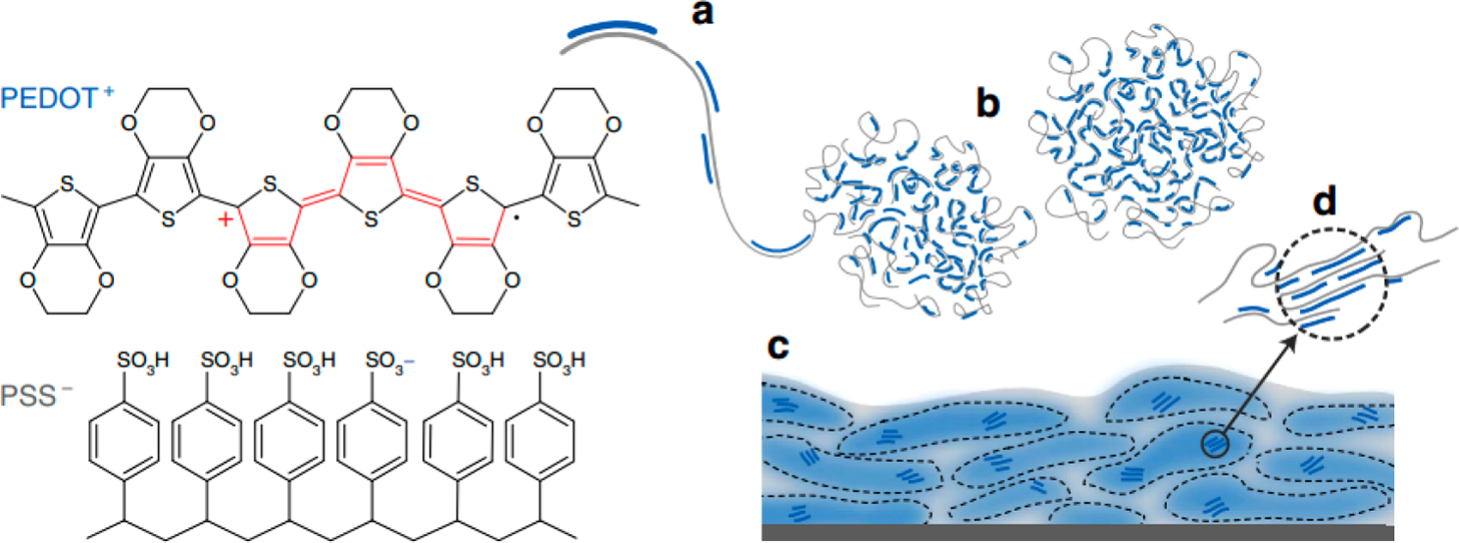
Schematic highlighting the microstructure of PEDOT:PSS, including (a) the synthesis of short PEDOT segments (blue) onto the PSS template (gray), (b) the formation of colloidal particles in a dispersion, and (c) resulting PEDOT:PSS films with PEDOT:PSS-rich (blue) and PSS-rich (gray) phases. (d) Inset showing the formation of crystalline domains that benefit electronic charge carrier transport. Figure reproduced with permission from ref (50). Copyright 2016 Springer Nature under CC BY license https://creativecommons.org/licenses/by/4.0/.

**Figure 12:**
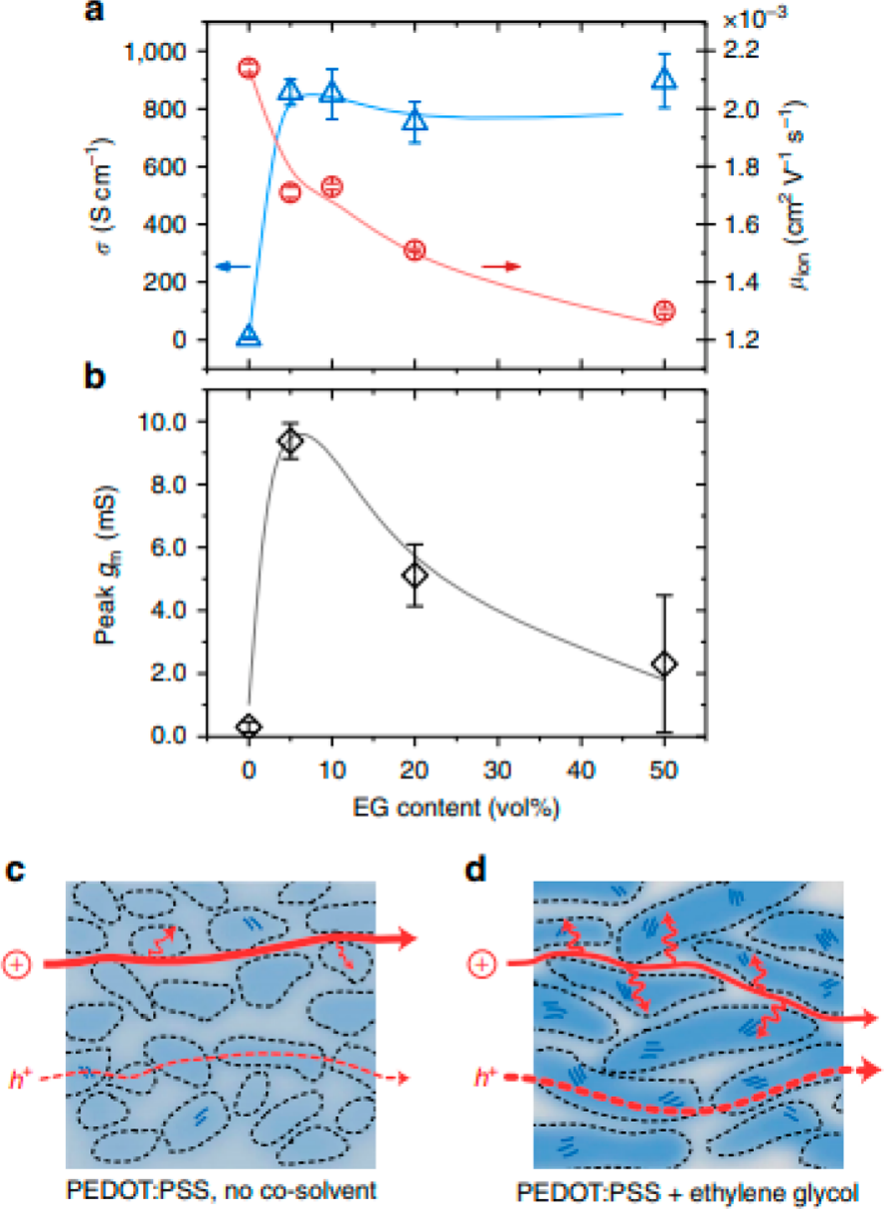
(a) Electrical conductivity (blue trace) and K^+^ ion mobility (red trace) of PEDOT:PSS as a function of ethylene glycol formulation content. (b) Transconductance of the PEDOT:PSS-based OECT as a function of ethylene glycol formulation content. (c and d) Schematic highlighting the impact of ethylene glycol addition on the morphology of PEDOT:PSS and the associated changes in ionic and electronic charge carrier transport. The width of the red arrows denotes the relative ease of electronic and ionic charge carrier transport across the material. Figure adapted with permission from J. Rivnay et al.^50^ Copyright 2016 Springer Nature.

In bioelectronic applications, PEDOT:PSS films are typically obtained by spin-coating from commercially available PEDOT:PSS aqueous dispersions. Structurally, PEDOT:PSS’s film morphology has been reported to be composed of PEDOT-rich domains dispersed in a PSS-rich matrix; see Figure 11.^155−157^ In their pristine form, films cast from these dispersions typically incur low conductivity values (0.1–1 S cm^–1^), thus rendering them unsuitable for most electronic applications.^50,99,103,158^ The addition of molecular additives, such as common organic solvents (e.g. ethylene glycol (EG), glycerol, *N*-methylpyrrolidone, DMSO, etc.) often referred to as secondary dopants, to PEDOT:PSS casting dispersions is, however, an effective treatment to improve their electrical conductivities by over 3 orders of magnitude (Figure 12).^159,160^ In fact, such additives are also frequently used when preparing PEDOT:PSS films for OECT applications.^50,161−163^ Studies focused on the use of EG as a secondary dopant to boost OECT performance revealed that increasing the content of the EG additive has multiple parallel effects on the film microstructure at both the molecular scale and mesoscale.^50^ In particular, EG content increases have been shown by grazing incidence wide-angle X-ray scattering (GIWAXS) to incur slightly tighter π–π stacking across different polymer chains and increase crystallite size. The growth in domain sizes was accompanied by increases in the heterogeneity of the PEDOT:PSS cores and PSS-rich matrices making up the films’ microstructure as confirmed by combined near edge X-ray absorption fine structure (NEXAFS) and resonant soft X-ray scattering (rSOXS) analyses. The EG-induced coarsened and more heterogeneous microstructure has a direct impact on both the recorded electronic and ionic charge carrier mobilities and, therefore, also on the final OECT device performance. In fact, while the coarser morphologies were detrimental toward ionic charge carrier mobilities (*μ*_*ion*_ ∼ 2.2 × 10^–3^ cm^2^ V^–1^ s^–1^ for the blend containing 0 v/v% EG to ∼1.3 × 10^–3^ cm^2^ V^–1^ s^–1^ for the blend containing 50 v/v% EG), they were found to significantly boost the electrical conductivity from 6 to 800 S cm^–1^, ultimately requiring a careful balancing in these opposing factors to maximize the recorded OECT performance. This concept was well reflected in the OECTs’ transconductances recorded for the various blends, with the blends containing 0, 20, and 50 v/v% of the EG additive incurring an average *g*_m_ of ∼1.2, ∼6.5, and ∼3.2 mS, respectively. On the other hand, the blend employing 5 v/v% EG additive yielded the highest transconductance of ∼8.0 mS. Further evaluation of the blends in terms of μ, C*, and μC* confirmed the reported trend with the highest values again reported for the 5 v/v% EG-containing blend, which incurred a μ of 1.9 cm^2^ V^–1^ s^–1^ a C* of 39 F cm^–3^, and a μC* of 47 F cm^–1^ V^–1^ s^–1^;^28^ see Table 1.

**Table 1:** OECT Performance Summary of Various PEDOT:PSS-Based Conjugated Polymer/Polyelectrolyte Composites

OMIEC	*g*_m_ (mS)	*L* (μm)	*W* (μm)	*d* (nm)	*g*_m*,*norm_ (mS μm^–1^)^a^	μ (cm^2^ V^–1^ s^–1^)	C* (F cm^–3^)	μC* (F cm^–1^ s^–1^ V^–1^)	ref
PEDOT:PSS + 0 v/v% EG	1.2	50	50	390	3.08	0.20	36		(50)
PEDOT:PSS + 5 v/v% EG	∼8.0	50	50	174	45.98	1.90	39	47	(28, 50)
PEDOT:PSS + 20 v/v% EG	∼6.5	50	50	208	31.25	1.45	31		(50)
PEDOT:PSS + 50 v/v% EG	∼3.2	50	50	184	17.39	1.45	21		(50)
PEDOT:PSS + DMSO SA	∼2	300	1000	69	8.70	0.13			(164)
PEDOT:PSS + DMSO SVA	∼4.4	300	1000	100	13.20	0.19			(164)
PEDOT:PSS + DMSO SPT	7	300	1000	110	19.09	0.44			(164)
PEDOT:PSS/[EMIM][TCM]	∼14.2	100	1000	200	7.10	3.80	87	335	(168)
PEDOT:PSS + 0.05 wt % GOPS	∼0.8	50	50	62	12.90	6.40			(173)
PEDOT:PSS + 1 wt % GOPS	∼0.8	50	50	90	8.89	4.70			(173)
PEDOT:PSS + 2.5 wt % GOPS	∼0.4	50	50	130	3.08	4.50			(173)
PEDOT:PSS + 5 wt % GOPS	∼0.05	50	50	180	0.28	1.70			(173)
PEDOT:PSS + 3 v/v% DVS	13.2	10	100	100	13.20				(176)
Pr-P		20	80						(178)
EG-P	4	20	80	190	5.26		31	100	(178)
Crys-P	19	20	80	200	23.75	4.34	113	490	(178)
PEDOT:TOS						0.93	136	72	(28)
PEDOT:PSTFSILi100	3.41	10	100	200	1.71	0.23	26	20	(28, 183)
PEDOT:PSTFSIK250	2.58	10	100						(183)
PEDOT:PSTFSIK20	3.05	10	100						(183)
PEDOT:PMATFSILi80	1.65	10	100						(183)
PEDOT:DS						0.0064	65	2.2	(28)
PEDOT:Nafion	6	1500	1000	30000	0.30				(184)
PEDOT:Nafion	0.28	1	4						(185)
PEDOT:DNA	0.16	60	2000	263	0.02				(170)

a Values calculated by dividing *g*_m_ by the product *Wd/L*.

Various DMSO treatment methods, including the solvent additive (SA), solvent vapor annealing (SVA), and solvent post-treatment (SPT) methods, have also been evaluated to improve the performance of PEDOT:PSS films for OECT applications.^164^ Specifically, these involve either mixing PEDOT:PSS with DMSO before film deposition (SA), exposing a cast PEDOT:PSS film to a saturated DMSO vapor (SVA), or dipping a cast PEDOT:PSS film into a DMSO bath. From these studies a performance order of SPT > SVA > SA was determined, with the SPT, SVA, and SA treatments incurring PEDOT:PSS-based OECTs with maximum *g*_m_ values of ∼7, ∼4, and ∼2 mS, respectively. The superior performance of SPT treated films was ascribed to the increased phase segregation of PEDOT:PSS into PEDOT-rich and PSS-rich domains and enhanced structural order.

**Figure 13:**
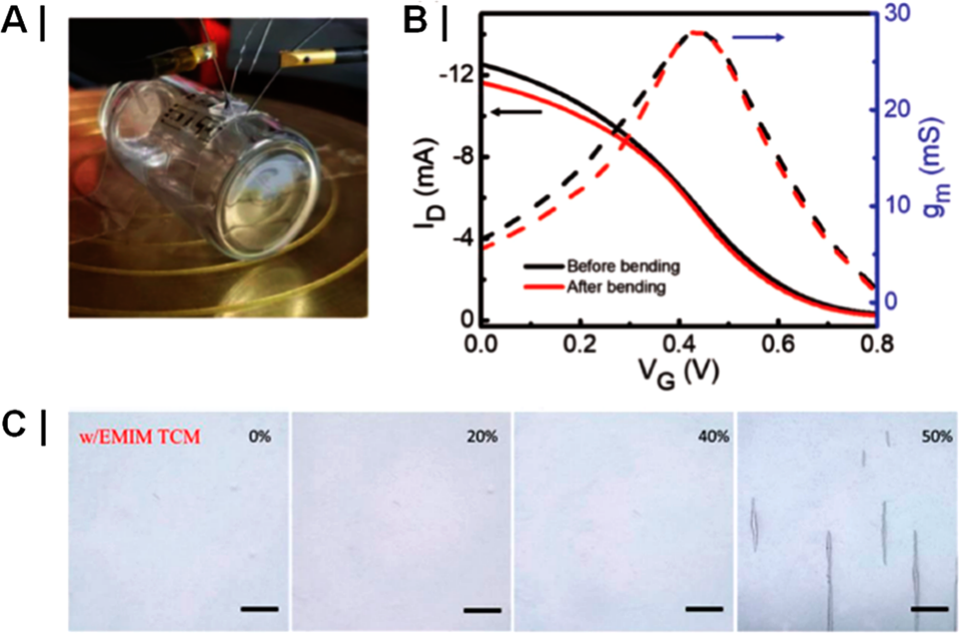
(a) Bending of PEDOT:PSS/[EMIM][TCM]-based OECTs. (b) Transfer curves of PEDOT:PSS/[EMIM][TCM]-based OECTs prior to and after bending. (c) Optical micrographs of PEDOT:PSS/[EMIM][TCM]-based films under various strains. Figure adapted with permission from ref (168). Copyright 2019 John Wiley and Sons.

A different class of secondary dopants that has been successfully employed to maximize the performance of PEDOT:PSS-based OECTs is ionic liquids, which have previously been demonstrated to boost the electrical conductivity of PEDOT:PSS to values > 1000 S cm^–1^.^165−167^ When adding 1.5 wt % 1-ethyl-3-methylimidazolium tricyanomethanide [EMIM][TCM] to a PEDOT:PSS solution prior to casting on devices, the performances of the resulting PEDOT:PSS/[EMIM][TCM] films were found to incur a μC* figure of merit of 335 F cm^–1^ V^–1^ s^–1^, translating into an approximately 4-fold performance enhancement compared to the best performing PEDOT:PSS film treated with ethylene glycol.^168^ Specifically, [EMIM][TCM] addition was found to benefit both the films’ volumetric charge storage properties and electronic charge carrier mobilities, given the recorded C* of 87 F cm^–3^ and the μ of 3.8 cm^2^ V^–1^ s^–1^. These features were in turn ascribed to the formation of tighter PEDOT π–π stacking distances and fibrillar morphologies. One significant difference between treating PEDOT:PSS films with [EMIM][TCM] or EG arises from [EMIM][TCM]’s nonvolatile nature, resulting in [EMIM][TCM] persisting within the cast film. The benefit thereof is that [EMIM][TCM] can act as a plasticizer, thereby resulting in good stretchability and flexibility of the resulting PEDOT:PSS/[EMIM][TCM] films, which were shown to withstand strains up to 40% and bending radii of 5 mm. Finally, PEDOT:PSS/[EMIM][TCM] devices also incurred excellent stabilities retaining 93% of their initial drain current values after 500 electrochemical switching cycles and ∼90% of their maximum transconductance after storage in ambient conditions for one month (Figure 13).

##### 6.1.2.2 Processability Additives—Surfactants and Cross-linkers

**Figure 14:**
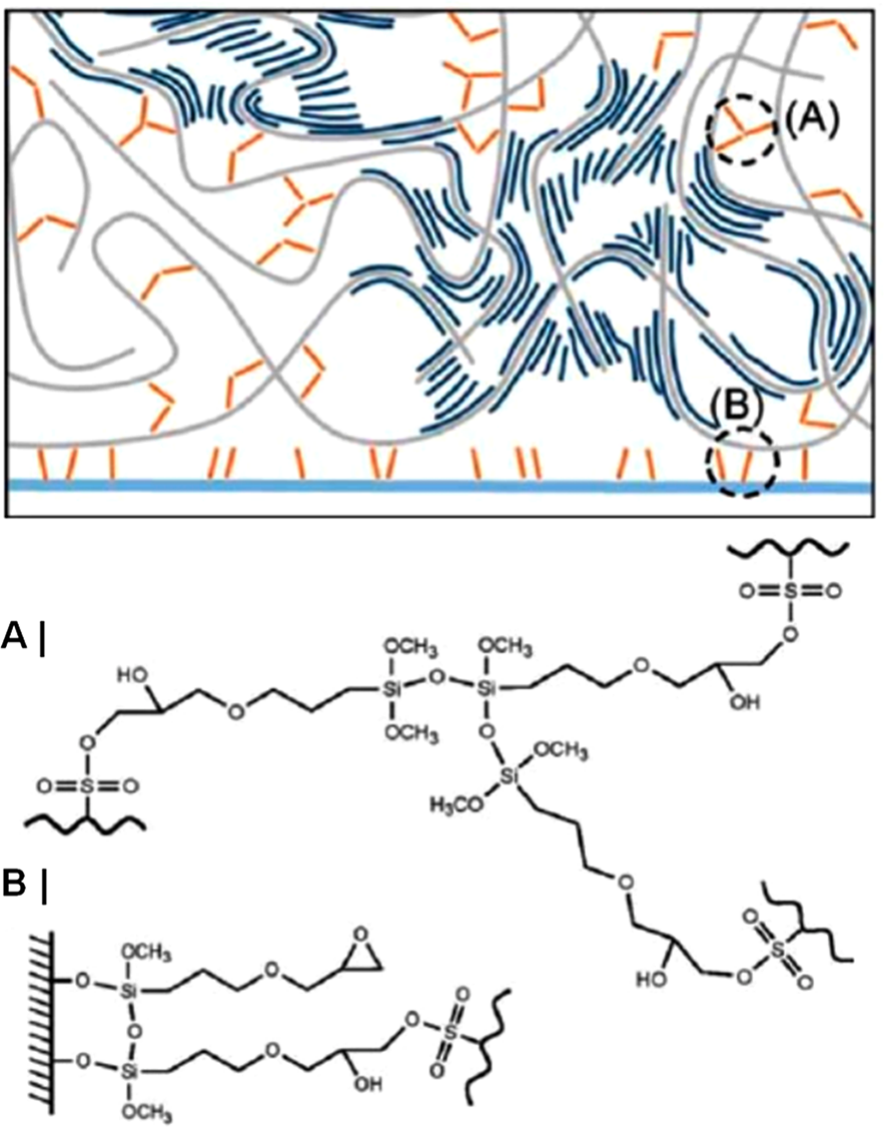
Schematic highlighting the cross-linking mechanism of GOPS when blended with PEDOT:PSS onto a glass substrate. Figure adapted with permission from ref (172). Copyright 2017 John Wiley and Sons.

Other additives commonly employed in PEDOT:PSS composites to ensure good OECT performance are the molecular surfactant 4-dodecylbenzenesulfonic acid (DBSA) and the cross-linking agent 3-glycidoxypropyltrimethoxysilane (GOPS) (Figure 14). Unlike the aforementioned secondary dopants, the primary purpose of these additives is not to boost OECT performance but instead to facilitate organic semiconductor processing or thin-film stabilization onto device substrates.^26,161,169−173^ Typical DBSA ratios used for depositing PEDOT:PSS layers onto devices lie below 0.5 v/v%, although higher DBSA concentrations have been shown to enhance PEDOT:PSS’s electrical conductivity. The gains in PEDOT:PSS’s electrical performance are, however, offset by the increased difficulty in fabricating high-quality films, which has been ascribed to increased phase separation in the polyelectrolyte blend.^161^

Given PEDOT:PSS’s hydrophilicity, thin-film stabilization through the use of the silane-based cross-linking agent, GOPS, is often required to prevent thin-film delamination upon immersion and/or operation in aqueous electrolytes.^163,173^ Due to the presence of multiple components in the deposited films, GOPS’s exact cross-linking mechanism is yet to be fully elucidated, especially as cross-linking reactions with glass substrates have also been suggested to occur. According to the literature, the methoxy groups in GOPS can react with surface silanol groups present in silicon-based surfaces (e.g. glass).^174,175^ In parallel, the highly strained nature of GOPS’s epoxide ring and the availability of the C–O σ* orbitals render it reactive toward numerous nucleophiles, including water, alcohols (e.g. ethylene glycol), thiols, amines, and acids (e.g. the toluene sulfonic acid group in PSS) to form network-like structures. In terms of electrical conductivity and OECT transconductance, it has been demonstrated that minimizing the amount of GOPS employed is beneficial, as increasing the employed GOPS concentration from 0.05 wt % to 5 wt % resulted in an electrical conductivity drop from ∼460 to ∼120 S cm^–1^ and an even more pronounced decrease in the recorded transconductance from ∼13 to ∼0.4 mS μm^–1^.^173^ The decrease in PEDOT:PSS’s electrical performance upon increasing GOPS concentrations has been ascribed due to an effective dilution of the conducting phase upon the introduction of the electrically insulating cross-linker and the extended cross-linked network preventing the effective stacking of conjugated polymer backbone chains. For applications involving the transport of mixed charge carriers, it is also important to evaluate the effects of GOPS introduction in terms of ion mobility. In this context, introduction of 1 wt % GOPS into PEDOT:PSS films was shown to significantly reduce the extent of volumetric swelling of the composite upon electrochemical addressing, with the pristine film exhibiting a volume increase of 155% and the GOPS-containing film one of only 35%, thereby also affecting the film’s ability to uptake water.^171^ A direct consequence of the film’s reduced ability to take up water molecules was a reduced ion mobility, which compared to the pristine film was lowered by 1 order of magnitude from 1.4 × 10^–3^ to 1.9 × 10^–4^ cm^2^ V^–1^ s^–1^ for potassium ions. From the above it follows that keeping GOPS concentrations at a minimum is favorable in terms of not only electrical but also ionic charge carrier transport.

An alternative cross-linker to GOPS is divinylsulfone (DVS), which exhibits a higher reactivity toward nucleophiles (e.g. alcohols, amines, ...), therefore enabling cross-linking to be performed at significantly reduced temperatures (either at room temperature for 14 h or at 50 °C for 1 h) compared to GOPS (140 °C for 1 h).^176,177^ From a mechanistic standpoint the cross-linking in DVS is hypothesized to occur by nucleophilic attack of the cosolvents and/or secondary dopants (e.g. ethylene glycol) present in the commercially available PEDOT:PSS dispersions onto the unsaturated C=C bond in DVS, thereby leading to a cross-linked network featuring ether linkages. A clear advantage of using DVS as cross-linker compared to GOPS is that DVS incorporation into PEDOT:PSS composites does not have determinative effects on the composites’ electrical properties but instead helps improve their electrical conductivities from approximately 400–500 S cm^–1^ in the GOPS control to values between 550 and 750 S cm^–1^ in the DVS cross-linked composite. Moreover, compared to GOPS cross-linked films, in which the fraction of GOPS present has a dramatic effect on the recorded electrical conductivity, the electrical performance of DVS cross-linked films appears to be less sensitive to the fraction of incorporated DVS, with thin films retaining high electrical conductivities ∼700 S cm^–1^ during addition of almost 10 v/v% DVS. The suitability of DVS as a cross-linking agent for PEDOT:PSS films for OECT applications was confirmed by devices incurring high transconductance values up to 13.2 ± 0.65 mS, which compares favorably relative to the highest performing devices employing GOPS as a cross-linker, for which transconductance values between 2 and 3 mS were reported. The shelf life stability of DVS cross-linked PEDOT:PSS-based OECTs was also high, with devices retaining 94% and 80% of their initial transconductance after 20 and 64 days of storage, respectively. Finally, PEDOT:PSS films making use of the DVS cross-linker were also demonstrated to be biocompatible through cytotoxicity tests, with samples showing identical cell viabilities compared to that of the inert control. Nonetheless, one significant limitation of using DVS as a cross-linker for PEDOT:PSS composites is that DVS is only able to form cross-links within the conjugated polymer network, but not with the employed substrate, thus also necessitating the addition of 0.2% GOPS into the blend to ensure suitable substrate adhesion.

##### 6.1.2.3 Concentrated Sulfuric Acid Treatment

**Figure 15:**
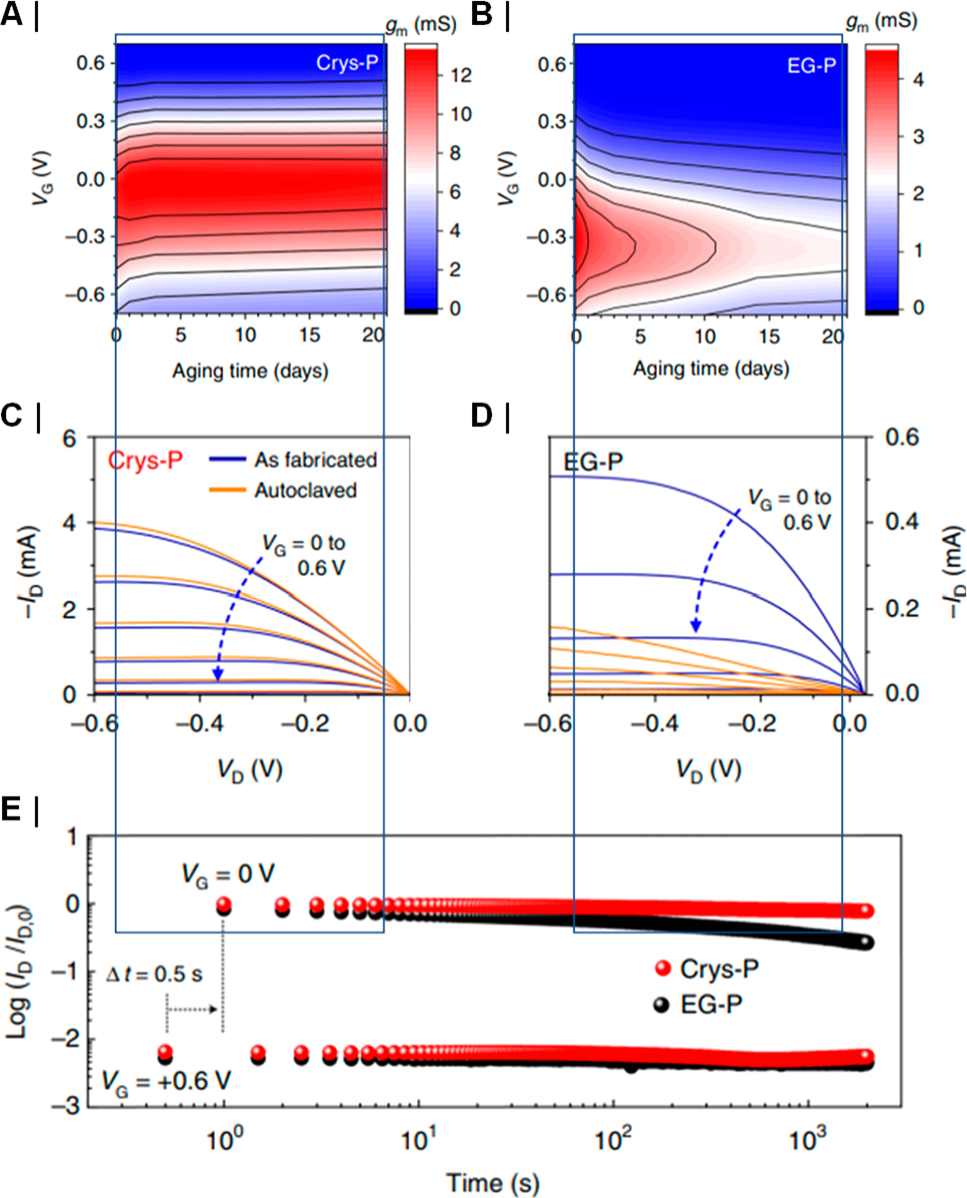
(A and B) Stabilities of Crys-P and EG-P in aqueous media over 21 days. (C and D) Stabilities of Crys-P and EG-P following autoclaving sterilization. (E) Operational stabilities of Crys-P and EG-P following 2000 electrochemical switching cycles. Figure adapted with permission from ref (178). Copyright 2018 Springer Nature.

A more recent approach combines high performance with high stability, and therefore, the benefits incurred by secondary dopants and cross-linking agents involve postdeposition treatment of as-spun PEDOT:PSS films with concentrated sulfuric acid.^178^ This approach has previously also been deployed to incur PEDOT:PSS films with excellent electrical conductivities (up to 4200 S cm^–1^) and charge carrier mobilities (>4 cm2 V^–1^ s^–1^).^179,180^ As shown by GIWAXS, concentrated sulfuric acid treatment was shown to induce a significant structural rearrangement in the PEDOT:PSS film (Crys-P) leading to enhanced crystallinity and highly anisotropic molecular ordering compared to its pristine form (Pr-P) and to an ethylene glycol treated (EG-P) reference film (Figure 15). Furthermore, the acid treatment also led to a preferential edge-on alignment of the PEDOT chains in Crys-P, which is likely to enhance OECT performance by facilitating the interchain transport of charge carriers in the in-plane direction. X-ray photoelectron spectroscopy (XPS), on the other hand, highlighted that in addition to these morphological changes, solvent-assisted crystallization of the PEDOT:PSS films also led to significant changes in the chemical composition of the films, with the Crys-P films featuring a three times lower molar ratio of styrenesulfonate units compared to the Pr-P and EG-P references, in turn minimizing the fraction of electrically insulating component within the conjugated polymer composite. In combination, these changes in the films’ microstructures and chemical compositions resulted in an almost 5-fold OECT performance increment in the Crys-P films (19 mS) compared to the EG-P reference (4 mS), which was confirmed by evaluation of the respective μC* values (490 ± 41 F cm^–1^ V^–1^ s^–1^ for Crys-P and 100 ± 7 F cm^–1^ V^–1^ s^–1^ for EG-P). Extensive stability measurements, including shelf life stability evaluation, repeated electrochemical addressing, and thermal shock through autoclave sterilization, highlighted that in addition to improved OECT performance, solvent-assisted crystallization was also able to improve OECT stability. In fact, compared to the EG-P films, Crys-P films featured a more robust stability under each of the three stressing conditions.

##### 6.1.2.4 Counterion Variation

Another convenient synthetic approach to alter the electrical properties of PEDOT-based composites is through substitution of the PSS counterion. In this context a vast array of alternative counterions have been explored, including both molecular (e.g. tosylate (TOS), hexafluorophosphate, perchlorate, etc.)^144,154,181,182^ and polymeric counterions (e.g. lithium poly[(4-styrenesulfonyl)(fluorosulfonyl)imide] (PSTFSILi), hyaluronic acid, deoxyribonucleic acid (DNA), Nafion, etc.).^60,170,183−185^ Detailed OECT performance evaluation of these alternative PEDOT-based composites has, however, remained scarce in the literature, with few reports highlighting structure–property relationships. Moreover, with the exception for PEDOT:TOS, counterion substitution does, in general, not yield PEDOT-based composites with superior electrical properties compared to the PEDOT:PSS reference. Instead the benefits of using alternative charge compensating counterions stem from alternative desirable features such as reduced acidity to minimize printhead corrosion,^183,186^ the induction of specific cellular interactions (e.g. cell adhesion, cell growth support, ...),^141^ and the hydrophilic character^187^ and optical properties^188^ of the materials.

### 6.2 Conjugated Polyelectrolytes

Conjugated polyelectrolytes are a class of OMIECs in which ionic conductivity is achieved through covalent attachment of hydrophilic charged moieties in the CP’s side chains. Analogous to their alkylated counterparts, grafting of these side chains onto the conjugated backbone results in these materials being solution processable. Notably, however, while their alkyl equivalents tend to be soluble in traditional organic solvents employed for CP processing (such as aromatic hydrocarbons or chlorinated solvents), conjugated polyelectrolytes do not tend to be soluble in these solvents but instead require higher polarity solvents such as dimethyl sulfoxide, methanol, or water to be dissolved and processed.^189,190^ This aspect of conjugated polyelectrolytes has been exploited in numerous organic electronic devices, where sequential deposition of electroactive layers without morphology degradation of the underlying layers is of importance. A specific example of this concept can be observed in organic photovoltaic technologies, where the solvent orthogonality of conjugated polyelectrolytes and the active layer blend allows for the facile utilization of conjugated polyelectrolytes as interlayers leading to improved device performances.^191−193^ Moreover, the processability of these materials from less toxic and more environmentally benign solvents is also beneficial in terms of industrial upscaling. Several different types of charged moieties have been explored in the wider organic electronics literature, including those bearing positive,^192,193^ negative,^194−196^ or both positive and negative charges (i.e., zwitterionic species).^197−200^ Conjugated polyelectrolytes can further be subdivided into various classes depending on whether the conjugated backbone of the polymer under study is in its charged and therefore conductive state or charge neutral and therefore insulating under ambient conditions. This property of conjugated polyelectrolytes is strongly linked to their energy levels and in particular their highest occupied molecular orbital (HOMO).

#### 6.2.1 Conducting Backbone Conjugated Polyelectrolytes

**Figure 16:**
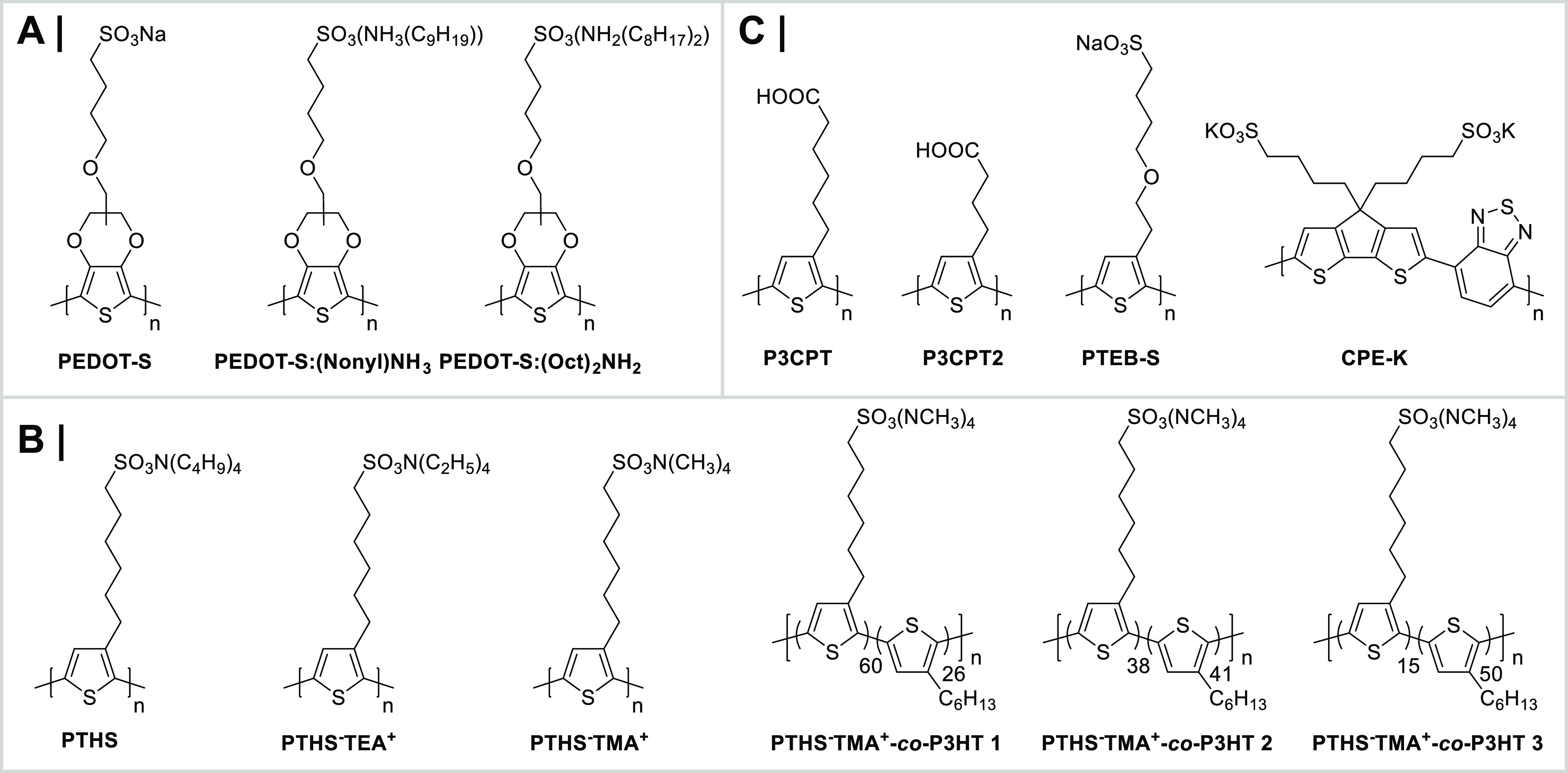
(A) Chemical structures of PEDOT-S-based conjugated polyelectrolytes. (B) Chemical structures of PTHS-based conjugated polyelectrolytes. (C) Chemical structures of miscellaneous conjugated polyelectrolytes.

Conjugated polyelectrolytes whose polymer backbone is in its conducting state share many features with PEDOT:PSS, most notably their depletion mode of operation, thus requiring the application of a bias at the gate electrode to turn the devices off. Unlike PEDOT:PSS, however, the absence of a bulky and electrically insulating PSS phase should translate to better charge storage capacities of these polymers, thus translating to higher device performances and improved signal recording abilities in neural applications. One early example of such a material is PEDOT-S (see Figure 16), whose use as an OECT channel material came only several years after its original synthesis.^201^ Similarly to the 3,4-ethylenedioxythiophene (EDOT) monomer employed in the synthesis of PEDOT:PSS, PEDOT-S’s corresponding monomer can also be employed to synthesize water-soluble polyelectrolytes through either electrochemical^202,203^ or chemical polymerization techniques.^204−206^ For its chemical synthesis PEDOT-S is typically prepared by oxidative polymerization employing FeCl_3_ as catalyst. A particular advantage of preparing PEDOT-S by such means is the increased flexibility in terms of film deposition on a broader range of substrates, including OECTs. OECTs based on PEDOT-S have been fabricated successfully, incurring transconductances as high as 16.2 mS at a *V*_G_ around +0.2 V. Interestingly, unlike for PEDOT:PSS-based systems, for which cross-linkers such as GOPS are typically employed to prevent dissolution of the water-soluble channel material during operation in an aqueous medium, PEDOT-S-based OECTs were not operated employing an aqueous electrolyte but instead circumvented dissolution in aqueous media by resorting to a solid-state ionic-liquid-based electrolyte.^201,207^ Similarly to PEDOT:PSS, PEDOT-S also operates in depletion mode, with increasing positive gate voltages leading to a reduction in current passed through the device. Due to their depletion mode of behavior, PEDOT-S-based OECTs yielded similar on/off ratios in the order of 10^2^–10^3^ as those that are typically achieved with PEDOT:PSS-based OECTs.^168,208^ To overcome the limitations incurred by its depletion mode of operation, PEDOT-S was substituted with another conjugated polyelectrolyte, specifically sodium poly(2-(3-thienyl)ethoxy-4-butylsul-fonate) (PTEB-S), which despite also containing sodium sulfonate end groups on its side chains does not have a conjugated polymer backbone that is oxidized under ambient conditions.^201^ The PTEB-S noncharged conjugated backbone under ambient conditions can be traced back to its lack of a 3,4-ethylenedioxythiophene bridge. The substitution of the 3,4-ethylenedioxythiophene bridge with a single alkyl substituent at the 3-position of the thiophene ring prevents the bridging oxygen atoms from pushing additional electron density on the conjugated polymer backbone through a resonance effect, thereby increasing PTEB-S’s ionization potential to 5.0–5.3 eV^209^ and creating a thermodynamic barrier preventing it from being oxidized spontaneously under ambient conditions.^210^ OECT fabrication with PTEB-S did indeed lead to channels operating in accumulation mode as evidenced by the higher flow of current through the devices during application of increasingly negative gate voltages. An interesting aspect of PTEB-S’s operation is that two concurring processes may be responsible for its electrochemical doping, including cation ejection from the polymer phase into the electrolyte and anion injection from the electrolyte into the polymer phase. So far, the determination of which of these processes dominates has not yet been established and would certainly be of interest to boost the understanding of the doping mechanism for such materials. Ultimately, OECTs based on PTEB-S yielded maximum *g*_m_ values of 0.7 mS at a *V*_G_ of −0.8, which were lower compared to those based on PEDOT-S, even when considering the approximately 2-fold lower thickness of OECTs based on PTEB-S compared to PEDOT-S. In addition to lower OECT steady-state performances, PTEB-S-based OECTs also incurred lower device stabilities upon repeated electrochemical addressing. In fact, during preliminary OECT electrochemical cycling stability studies, PEDOT-S-based devices appeared to retain both their on and off currents well, while the on current in PTEB-S-based devices was reduced to approximately one-third of its initial value over the same pulsing time. Although these results might thus point toward the lower electrochemical cycling stability of PTEB-S-based devices, different voltage ranges were employed to evaluate the stability of devices, precluding for direct comparisons to be made regarding the charge carrier densities achieved during the doping and dedoping of the polymers. To combine the advantageous aspects incurred by the individual conjugated polyelectrolytes, namely the high *g*_m_ and electrochemical cycling stability of PEDOT-S and the accumulation mode of behavior of PTEB-S, PEDOT-S and PTEB-S were blended together in various mass ratios between 1:1 and 2:1. OECT based on both blends could be operated in accumulation mode and incurred similar maximum *g*_m_ values around 9.0–12.0 mS, while featuring similar geometries across each other and the PEDOT-S reference.

For electronic recording devices to be compatible with biological systems, it is a requirement that the constituent CP films are mechanically stable in water. Similarly to pristine PEDOT:PSS, the charged moieties present in PEDOT-S also resulted in its water solubility, thus preventing the use of aqueous-based electrolytes for OECT operation. To overcome this limitation of PEDOT-S, follow-up work by the authors involved counterion exchange with ammonium salts to modify PEDOT-S’s hydrophilic character.^211^ Dioctylammonium chloride ((Oct)_2_NH_2_Cl) and nonylammonium chloride ((Nonyl)NH_3_Cl) were deemed to be suitable species to instill sufficient hydrophobic character in the resulting PEDOT-S:(Nonyl)NH_3_ and PEDOT-S:(Oct)_2_NH_2_ polymers. Moreover, the use of both monoalkylated and dialkylated ammonium counterions also enabled evaluation of the effect of counterion steric hindrance on the electrochemical performance of the two polymers. Starting from PEDOT-S, PEDOT-S:(Nonyl)NH_3_ and PEDOT-S:(Oct)_2_NH_2_ were obtained by a counterion exchange-precipitation method,^212^ in which precipitation of the counterion exchanged materials provided a kinetic driving force to shift the equilibrium reaction toward the products. Following counterion exchange, the resulting polymers were no longer water-soluble but could instead be processed effectively through the use of organic solvent mixtures. OECTs of PEDOT-S:(Nonyl)NH_3_ and PEDOT-S:(Oct)_2_NH_2_ could be operated successfully for several cycles in an aqueous-based electrolyte, highlighting the success of counterion exchange imparting sufficient hydrophobic character in the polymers. Maximum transconductances of 11.0 mS and 7.0 mS were recorded for PEDOT-S:(Oct)_2_NH_2_ and PEDOT-S:(Nonyl)NH_3_, respectively, whereby PEDOT-S:(Nonyl)NH_3_-based devices were approximately 50% thicker, consequently suggesting a performance difference closer to 2:1 between the two polymers. In addition to improved steady-state performances, PEDOT-S:(Oct)_2_NH_2_-based devices also demonstrated better transient characteristics, with no hysteresis being observed between the forward and backward scans. This was suggested due to PEDOT-S:(Oct)_2_NH_2_’s smoother and smaller grainlike morphological features as measured by atomic force microscopy (AFM). Higher electrochemical cycling stabilities were also recorded for PEDOT-S:(Oct)_2_NH_2_ with devices showing no signs of degradation over 10000 electrochemical switching cycles, compared to PEDOT-S:(Nonyl)NH_3_ only retaining 71% of the initial drain current after 1000 cycles across the same voltage range.

#### 6.2.2 Semiconducting Backbone Conjugated Polyelectrolyte

##### 6.2.2.1 Sulfonated Backbones

**Figure 17:**
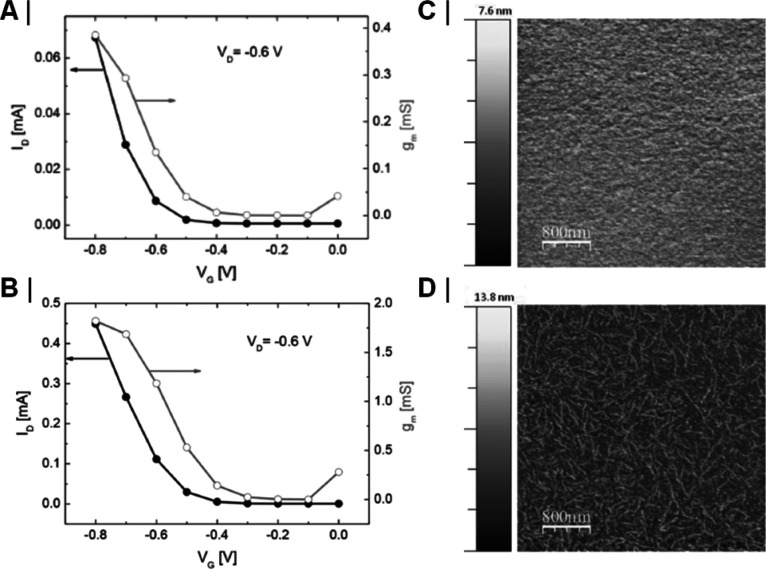
OECT performance and morphology of PTHS in the (A) absence and (B) presence of a 5% ethylene glycol additive. Figure adapted with permission from ref (190). Copyright 2014 John Wiley and Sons.

An early example of a conjugated polyelectrolyte designed specifically to operate in accumulation mode is the polythiophene PTHS (poly(6-(thiophen-3-yl)hexane-1-sulfonate)).^190^ Devices employing channel materials that enable accumulation mode behavior are particularly interesting to develop for sensing applications, as their presence in the normal off state confers them with lower power requirements but also because analyte detection over a virtually negligible background signal should also result in superior device sensitivities.^213^ Analogously to the aforementioned conjugated polyelectrolytes, PTHS’s hydrophilicity was imparted by the terminal ionic sulfonate group grafted directly on its six-carbon-atom-containing alkyl side chain. The dominance of the sulfonate moiety to impart hydrophilicity in conjugated polyelectrolytes is unsurprising given sulfonic acid’s low p*K*_a_, around −2, which should lead to a reduced pH-dependent behavior under the typical pH range (1–14) in comparison to their carboxylic acid counterparts, who’s higher p*K*_a_, around 5, makes them susceptible to stronger pH-dependent electrochemical behavior.^214^ PTHS was synthesized by Grignard metathesis polymerization of its nonionic bromide precursor, thus avoiding the use of an expensive palladium transition metal catalyst and the synthesis of an organometallic coupling partner.^215^ The final polymer was subsequently obtained by postpolymerization functionalization of its alkyl bromide-containing precursor through a nucleophilic ammonium sulfite salt. One additional benefit of this polymerization technique is its ability to yield high-number-average-molecular-weight (*M*_*n*_) polymers and low dispersity values (*Đ*), which are typically beneficial in terms of the polymers’ resulting charge transport properties. In fact, PTHS was obtained with a *M*_*n*_ of 42.0 kDa and a *Đ* of 1.09. Analogously to PEDOT:PSS, the ionic sulfonate group present in PTHS’s side chain also resulted in PTHS being water-soluble; thus, during device fabrication, thermal cross-linking with the (3-glycidyloxypropyl)trimethoxysilane (GOPS) was necessary to stabilize the resulting polymer films during aqueous electrolyte exposure and OECT operation. OECTs fabricated with PTHS were operated employing a 0.1 M aqueous sodium chloride solution as the supporting electrolyte, whereby application of increasingly negative gate biases resulted in increasing currents flowing across the channel, thus confirming the polymer’s accumulation mode of behavior. From a chemical design point of view, this can be attributed to PTHS’s side chain not containing any chalcogen atoms that are in direct electronic communication with the polymer’s conjugated polymer backbone, thus not rendering PTHS susceptible to spontaneous oxidation under ambient conditions. This aspect was further reflected in spectroelectrochemical measurements of the polymer, in which application of a positive bias was necessary to suppress the polymer’s π–π* optical transition and concomitantly resulting in the appearance of longer wavelength absorption features pertaining to the polymer’s doped species, specifically the polymer’s polaron and bipolarons. Ultimately a maximum transconductance of 0.40 ± 0.02 mS was recorded for PTHS when cast with addition of 1% GOPS to stabilize the resulting film for OECT operation in an aqueous environment. A significant improvement in the polymer’s OECT performance was observed by incorporation of 5 v/v% of the additive ethylene glycol (EG), with devices of identical channel dimensions incurring 5-fold higher transconductances of 2.0 ± 0.2 mS (Figure 17). The improved OECT performance upon EG inclusion was ascribed to a similar effect as typically observed when including EG in PEDOT:PSS formulations, specifically the formation of a coarser polymer morphology resulting in increased long-range order and the formation of fiber-like structures. In a follow-up study, the polymer’s steady-state performance as ascribed by the metric μC* was calculated to be 5.5 F cm^–1^ V^–1^ s^–1^.^28^

To systematically evaluate the effects of counterion substitution on the electronic properties of PTHS-based polymers, a series of PTHS-based polymers containing either the original tetrabutylammonium (PTHS-TBA+), tetraethylammonium (PTHS-TEA+), or tetramethylammonium (PTHS-TMA+) counterion were synthesized.^216^ Counterion exchange was envisaged as a suitable strategy to alter both the electronic properties and aggregation behavior of the conjugated polyelectrolytes, thus leading to potential performance increments.^217,218^ In each case, the final polymer was obtained by postpolymerization functionalization of its alkyl bromide-containing precursor through a nucleophilic ammonium sulfite salt. Note how the same batch of alkyl bromide-containing precursor was employed for the synthesis of the resulting conjugated polyelectrolytes, thus also ensuring the presence of the same number of repeat units in each conjugated backbone and enabling for a robust comparison across the three polymers. UV–vis absorption spectroscopy of the pristine polymer thin films revealed a gradual bathochromic shift of the maximum absorption wavelength (*λ*_max*,*film_) upon reduction of the tetraalkylammonium counterion size, with PTHS-TBA+, PTHS-TEA+, and PTHS-TMA+ incurring *λ*_max*,*film_ values of 499, 540, and 549 nm respectively, thus pointing toward an increased degree of aggregation and molecular ordering. The presence of a red-shifted aggregation-induced shoulder in the absorption spectra of PTHS-TEA+ and PTHS-TMA+ further supported the above findings. Electrochemical evaluation of the polymers demonstrated counterion exchange also to affect the recorded ionization potential (IP) and thus the ability and tendency of the polymers to be doped electrochemically. The IPs recorded for PTHS-TBA+, PTHS-TEA+, and PTHS-TMA+ were 5.52, 5.50, and 5.43 eV, respectively. A consequence of lowering the IP upon decreasing counterion size was the polymers’ ability to undergo electrochemical doping more readily. In fact, as evidenced by spectroelectrochemical measurements, decreasing the counterion size resulted in progressively larger depressions of the main π–π* absorption feature and more intense absorption features in the near-infrared (NIR) and infrared areas, which are typically ascribed to the polymers’ polaronic and bipolaronic species.^219^ An interesting aspect to note from the combined above findings is that despite counterion size reduction resulting in the formation of more aggregated films, this did not adversely affect counterion insertion during the electrochemical doping of the polymers. These results thus suggest that counterion size reduction could improve not only the charge storage but simultaneously also the charge carrier transport abilities, which should result in significantly improved OECT performances. OECTs were fabricated with each polymer and confirmed the above hypothesis, with PTHS-TMA+ incurring not only the highest volumetric capacitance (C*) of 82 F cm^–3^ (cf. 22 and 5 F cm^–3^ obtained for PTHS-TEA+ and PTHS-TBA+, respectively) but also the highest electronic charge carrier mobility of 267 × 10^–9^ cm^2^ V^–1^ s^–1^ (cf. 8.21 × 10^–9^ and 8.18 × 10^–9^ cm^2^ V^–1^ s^–1^ obtained for PTHS-TEA+ and PTHS-TBA+, respectively). Ultimately, the maximum transconductance values recorded for PTHS-TMA+, PTHS-TEA+, and PTHS-TBA+ were 45.5, 2.16, and 0.13 μS, respectively.

**Figure 18:**
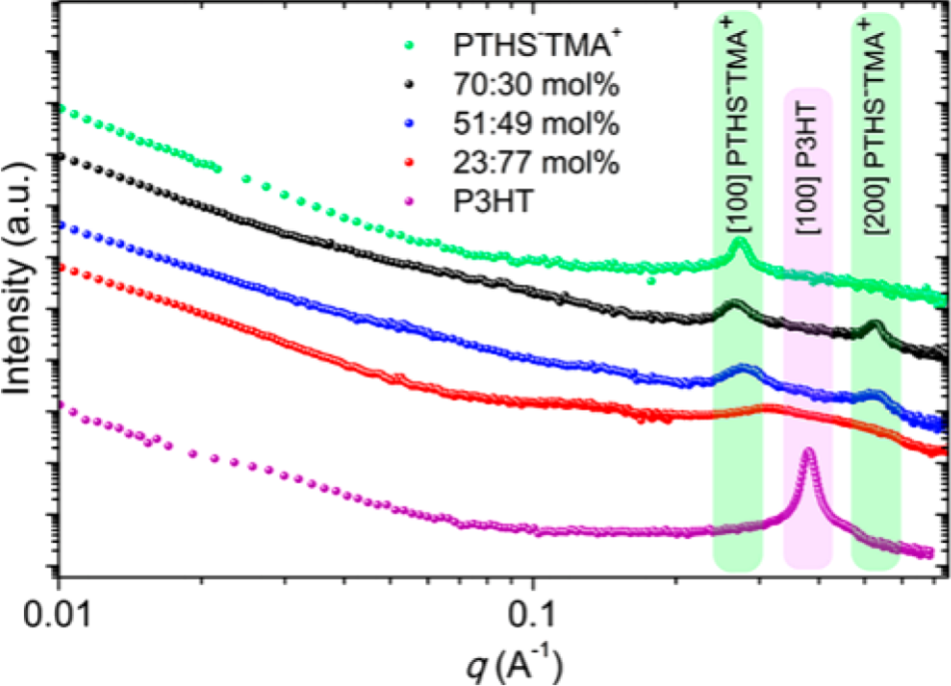
Small- and wide-angle X-ray scattering profiles recorded for PTHS-TMA+, PTHS-TMA+-*co*-P3HT 1, PTHS-TMA+-*co*-P3HT 2, PTHS-TMA+-*co*-P3HT 3, and P3HT. Figure adapted with permission from ref (220). Copyright 2019 American Chemical Society.

Further optimization of PTHS-based polymers was conducted by copolymerization of PTHS-TMA+ with varying degrees of poly(3-hexylthiophene) (P3HT),^220^ a conjugated polymer which has attracted considerable attention in several other organic electronic research areas due to its simple structure yet relatively good charge transport properties.^221−223^ Incorporation of various degrees of hydrophobic P3HT fractions into PTHS-TMA+-based polymers was portrayed as a promising method to control the degree of hydration and swelling in the materials, a task of utmost importance in organic mixed ionic–electronic conductors, where a balance between high ionic and high electronic charge carrier mobility typically has to be struck.^50^ Three gradient copolymers of PTHS-TMA+-*co*-P3HT, namely PTHS-TMA+-*co*-P3HT 1, PTHS-TMA+-*co*-P3HT 2, and PTHS-TMA+-*co*-P3HT 3, with P3HT contents of 30, 49, and 77 mol % were synthesized with comparable absolute molecular weights of 16 ± 3 kDa. Microstructure investigation of the polymers was conducted through small- and wide-angle angle X-ray scattering (SAXS and WAXS) (Figure 18).

As indicated from the SAXS and WAXS data, both PTHS-TMA+-*co*-P3HT 1 and PTHS-TMA+-*co*-P3HT 2 containing substantial fractions of PTHS-TMA+ gave rise to the same [100] lamellar stacking feature observed for pristine PTHS-TMA+ and an additional second order feature that was absent in PTHS-TMA+. On the other hand, the polymer containing the lowest fraction of PTHS-TMA+, PTHS-TMA+-*co*-P3HT 3, did not give rise to such lamellar scattering features, most likely due to insufficiently high fractions of PTHS-TMA+ present. Notably, no P3HT related scattering peak could be recorded in any of the three polymers. Therefore, while PTHS-TMA+ hinders the crystallization of P3HT in these gradient copolymers, P3HT seems to be aiding the crystallization of PTHS-TMA+. Ultimately, this resulted in PTHS-TMA+-*co*-P3HT 1 and PTHS-TMA+-*co*-P3HT 2 displaying a certain degree of order in the solid state, while PTHS-TMA+-*co*-P3HT 3 samples were largely disordered. The structural findings from SAXS were supported by the recorded thin-film UV–vis spectra of the polymers, with PTHS-TMA+-*co*-P3HT 1 and PTHS-TMA+-*co*-P3HT 2 exhibiting an aggregation-induced red-shifted shoulder that was absent in PTHS-TMA+-*co*-P3HT 3’s spectrum. In terms of conjugated polymer backbone energetics, P3HT content variation did not appear to have a significant impact given that similar IPs between 4.81 and 4.94 were recorded for the three polymers by photoelectron spectroscopy measurements in air (PESA). Despite the polymers featuring similar energy levels, their spectroscopic responses during electrochemical biasing in a 0.1 M aqueous sodium chloride solution varied significantly. Despite possessing the most ordered microstructures, PTHS-TMA+-*co*-P3HT 1 and PTHS-TMA+-*co*-P3HT 2 incurred the largest responses. In fact, an approximately 50% reduction in their main π–π* absorption around 530 nm could be observed when applying a positive bias of +0.9 V. On the other hand, only a 20% reduction in the same absorption feature at the same potential could be observed for PTHS-TMA+-*co*-P3HT 3, indicating its reduced propensity to undergo volumetric charging in an aqueous environment. This was attributed to the presence of excessive hydrophobic P3HT content, hindering the uptake of hydrated counterions. OECT fabrication and operation of the three polymers reflected each aspect of the characterization data. PTHS-TMA+-*co*-P3HT 3’s most hydrophobic nature was reflected in the lowest recorded C* value of 31 F cm^–3^ (cf. 107 and 100 F cm^–3^ obtained for PTHS-TMA+-*co*-P3HT 1 and PTHS-TMA+-*co*-P3HT 2). PTHS-TMA+-*co*-P3HT 3’s most hydrophobic nature also prevented the recording of reliable electronic transistor mobility data. The μ values of 0.009 and 0.017 cm^2^ V^–1^ s^–1^ recorded for PTHS-TMA+-*co*-P3HT 1 and PTHS-TMA+-*co*-P3HT 2, respectively, on the other hand, clearly illustrate the concept of the hydrophobic P3HT fraction being able to induce larger degrees of order in the polymer thin films.

##### 6.2.2.2 Carboxylated Backbones

**Figure 19:**
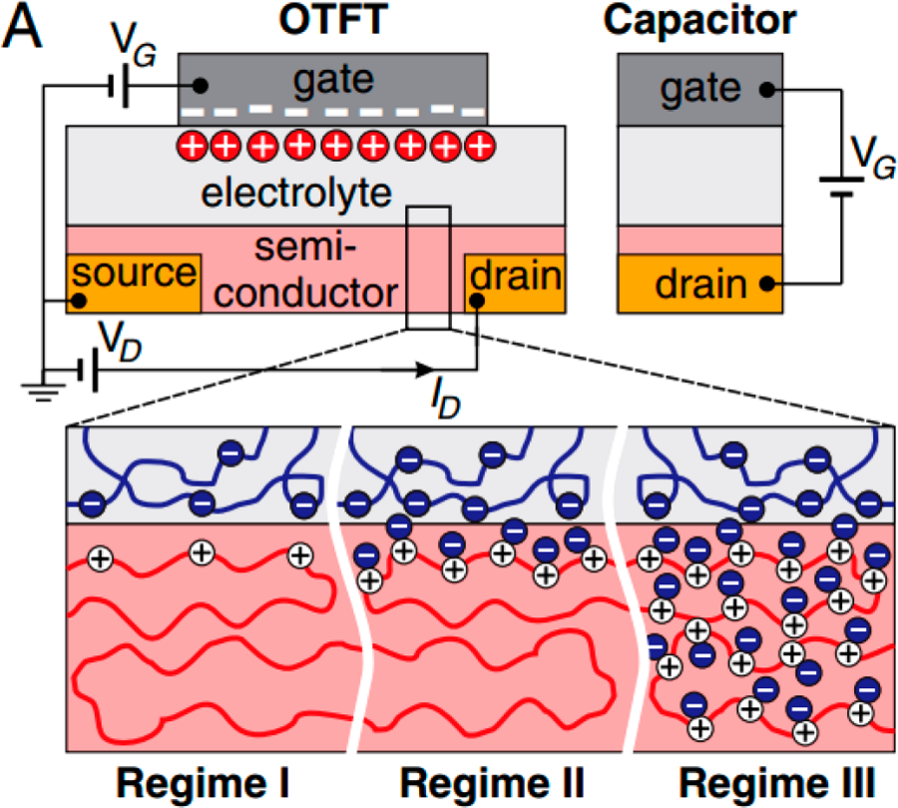
Different operating modes of electrolyte-gated OFETs employing P3CPT as channel material. Field-effect (regime I), interfacial electrochemical doping (regime II), and bulk electrochemical doping (regime III). Figure adapted with permission from A. Laiho et al.^189^ Copyright 2011 National Academy of Sciences.

Conjugated polymer backbones based on polythiophene have enjoyed considerable popularity in conjugated polyelectrolytes, an early example of such being poly(3-carboxypentylthiophene) (P3CPT), which compared to the aforementioned conjugated polyelectrolytes does not employ a sulfonate side group to impart hydrophilicity, but instead a carboxylic acid one.^189^ P3CPT, which had previously also been used as a photoactive component in dye sensitized solar cells (DSSCs)^224^ and a donor polymer in organic photovoltaics (OPV)^225^ and organic field-effect transistors,^226^ was originally tested in combination with a solid-state poly(vinylphosphonic acid-*co*-acrylic acid) electrolyte to assess its mode of operation in transistor-based devices. Here, it was found that three operational modes were possible, specifically a purely field-effect transistor mode, a purely electrochemical transistor mode, or a mixed field-effect/electrochemical transistor mode, whereby each of these could be addressed upon the application of different magnitudes and durations of voltages at the contact electrodes (Figure 19). Another early example of carboxylated backbones, PTAA-Li (poly(thiophene-3-acetic acid)), was used in combination with PEDOT in solar cells.^227^

In a follow-up study, P3CPT was investigated as an OECT channel material while switching to an aqueous-based electrolyte.^228^ The compatibility and successful operation of channel materials employing aqueous-based electrolytes are important given that these conditions correspond more closely to those found in biological milieus. OECTs fabricated with P3CPT could be operated successfully in a 0.1 M aqueous sodium chloride solution, yielding an accumulation mode of behavior and a maximum transconductance of 13.3 mS upon the application of a −0.6 V gate electrode bias. From a processing point view, P3CPT also demonstrated an advantage compared to its previously discussed sulfonate end group bearing counterparts, as the carboxylic acid terminal groups incurred a reduced hydrophilic nature compared to their sulfonate counterparts, thus foregoing the need to employ cross-linking agents when employing P3CPT as channel material for OECTs operating in aqueous media.

Further evaluation of carboxylic acid-containing conjugated polyelectrolytes as OECT channel materials was reported recently.^229^ Here a derivative of the original P3CPT, herein referred to as P3CPT2, employing a four- rather than six-carbon-atom chain, was synthesized and utilized as OECT channel material in aqueous media. Opposite to P3CPT, which was coated through spin-coating of polymer solutions in dimethyl sulfoxide, thin films of P3CPT2 were obtained following a previous literature procedure involving spray-casting of aqueous polymer solutions of its potassium salt followed by treatment with *p*-toluenesulfonic acid in methanol solution to achieve solvent resistance.^230,231^ A particular advantage of this coating process is its use of water, a solvent particularly well suited for the environmentally benign and green processing of organic semiconductors. The drawbacks of this coating technique are, however, its more complex nature and longer processing times that involve a 10 min acidification and 60 min drying step prior to the desired thin-film samples being obtained. OECTs based on P3CPT2 were fabricated, incurring a maximum *g*_m_ of 26 mS at a *V*_G_ of −0.6 V, similarly to those based on P3CPT. Although a direct steady-state performance comparison across the two materials is complicated, given the different device dimensions employed, estimates suggest a higher OECT performance of P3CPT2 compared to P3CPT, which may arise either from improved morphologies, charge storage capacities, or electronic charge carrier transport. The volumetric capacitance of P3CPT2 was indeed measured and estimated to be around 150 ± 18 F cm^–3^, thus comparing well to other conjugated polymers functionalized with ionic side chain motifs.^28,216,220^

##### 6.2.2.3 Donor–Acceptor Copolymer Backbones

As evidenced from the above discussion, the vast majority of conjugated polyelectrolytes developed in the context of OECTs employ a polythiophene conjugated backbone. A recent example of a conjugated polyelectrolyte employing an alternative conjugated π-system, specifically an alternating cyclopentadithiophene and benzothiadiazole one, is CPE-K.^232^ The alternation of electron-rich (cyclopentadithiophene) and electron-deficient (benzothiadiazole) conjugated building blocks results in the formation of a donor–acceptor (D-A) copolymer. Significantly higher charge carrier mobilities have been recorded for D-A copolymers in organic field-effect transistors (OFETs), compared to all-thiophene-based polymers, thus prompting the authors to extend this approach to OECTs.^233−241^ CPE-K was synthesized as previously reported through a Suzuki–Miyaura polymerization.^242^ Analogously to polythiophene-based conjugated polyelectrolytes featuring hydrophilic sulfonate groups, the sulfonate groups on each of CPE-K’s alkyl chains resulted in its solubility in water, thus also necessitating the use of GOPS to cross-link and stabilize thin films in aqueous media. Specifically, in-depth X-ray photoelectron spectroscopy (XPS) measurements revealed that GOPS cross-linking was predominantly caused by the nucleophilic attack of CPE-K’s sulfonate groups with the electrophilic carbon center at GOPS’s epoxide ring. OECTs featuring regular contacts were fabricated with CPE-K as the channel material and incurred a maximum *g*_m_ of 4 mS at a *V*_G_ of −0.55 V. The use of interdigitated electrodes resulted in a significant improvement in *g*_m_ up to 68.1 mS, whereby it has to be noted that this improvement is related to changes in device geometry rather than material-based improvements. Evaluation of *g*_m_ as a function of different device geometries allowed for a performance comparison against previously reported OECT channel materials, indicating that OECTs based on CPE-K incur steady-state performances similar to those of PTHS. While OECT electronic charge carrier mobilities were not reported for CPE-K, electrochemical impedance spectroscopy (EIS) measurements supported this conclusion, given that similar C* values of 134 and 124 F cm^–3^ were recorded for CPE-K and PTHS, respectively. From the above data it follows that conjugated polyelectrolytes making use of a donor–acceptor structure are a promising and exciting class of materials for OECT channel materials. As of now, no clear structure–property relationships have yet emerged for this class of polymers, thus requiring further investigations in the upcoming years.

### 6.3 Conjugated Polymers

Conjugated polymers can be used to address the limitations posed by PEDOT:PSS. Specifically, for OECT applications this includes overcoming PEDOT:PSS’s (i) depletion mode of operation, resulting in lower on–off ratios and increased power requirements, (ii) presence of an electrically insulating PSS phase, effectively diluting the fraction of electrochemically active material and typically limiting the volumetric capacitance of PEDOT:PSS to ∼40 F cm^–3^, (iii) necessity for extensive pre- and postprocessing treatments to achieve its maximum OECT performance, and (iv) highly complex structure, limiting its use as a model system for systematically investigating structure–property relationships.

**Figure 20:**
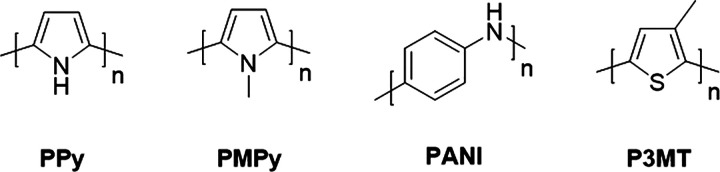
Chemical structures of early conjugated polymers used for OECT applications.

Early conjugated polymers used primarily in the 1980s and 1990s (although some such as polyaniline have been incidentally observed more than a century prior^243^) include structurally rather simple CPs such as polypyrrole,^244−249^ poly(*N*-methylpyrrole),^245,250^ polyaniline,^251−255^ and poly(3-methylthiophene);^256^ see Figure 20. Importantly, in the field of neuroelectrodes, nitrogen-containing polymers, such as polypyrrole, were among the earliest demonstrations of contact between conjugated polymers and neurons.^257,258^ While these materials were not necessarily tailored specifically for their use in OECTs, their deployment as channel materials laid the foundations of the field, providing initial insights into the physical processes governing OECT operation and basic chemical structure–property relationships to guide future material design. Since then, these materials have in large part been superseded by OMIECs specifically tailored for OECT applications, with ethylene glycol (EG) functionalized CPs representing the most well established and studied class of conjugated polymers for OECT applications. Such polymers also have had extensive use outside of OECTs, for some time, as reviewed by Andersson et al.^259^ Historically, due to these polymers being synthesized chemically, as opposed to electrochemically, this has allowed the production of better-defined samples (compared to electrochemically deposited polymers) for extensive physicochemical investigation via electrochemical means.^260^

**Figure 21:**
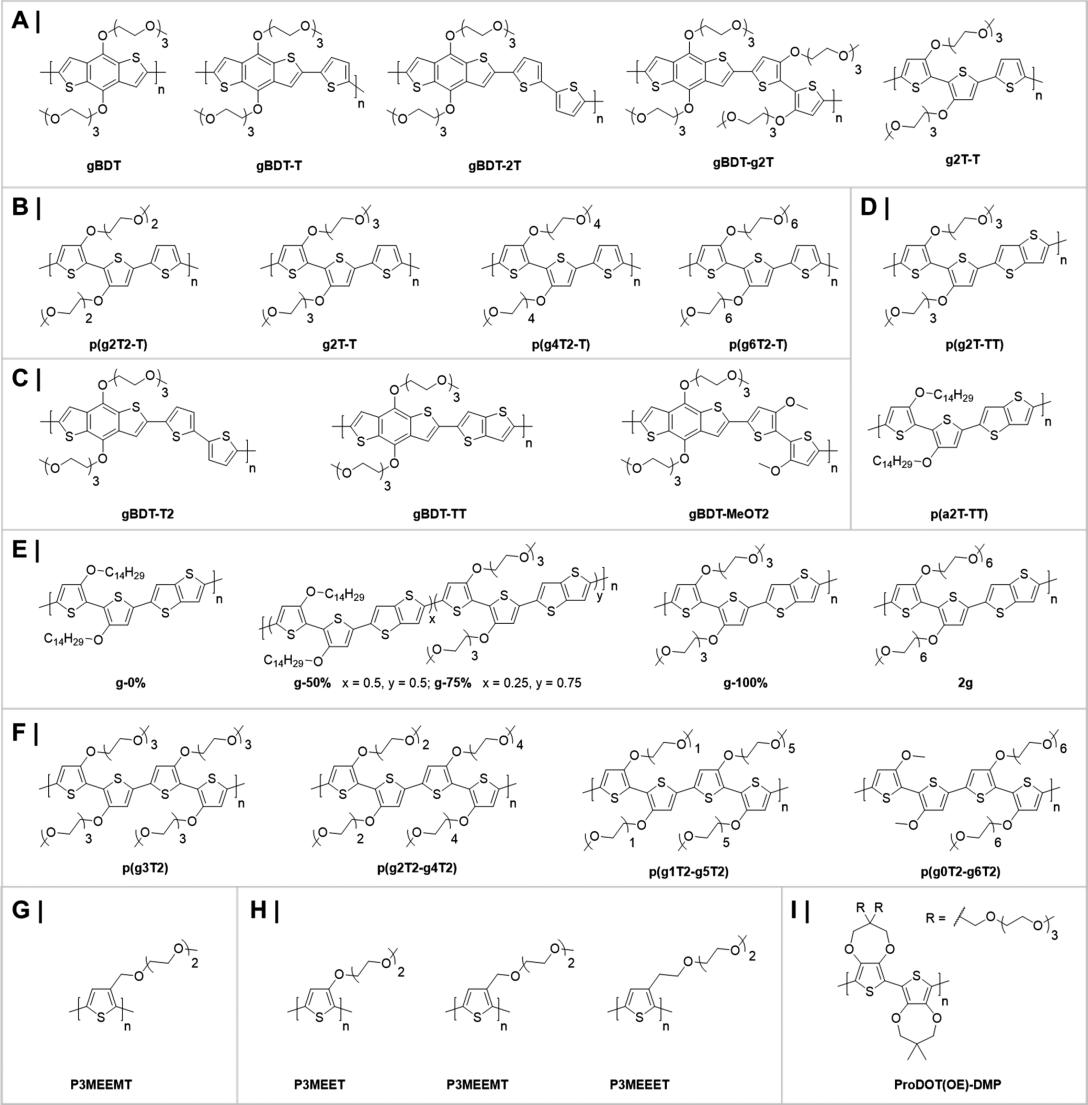
Chemical structures of (A) gBDT, gBDT-T, gBDT-2T, gBDT-g2T, and g2T-T;^261^ (B) p(g2T2-T), g2T-T, p(g4T2-T), and p(g6T2-T); (C) gBDT-T2, gBDT-TT, and gBDT-MeOT2; (D) p(g2T-TT) and p(a2T-TT); (E) g-0%, g-50%, g-75%, g-100%, and 2g; (F) p(g3T2), p(g2T2-g4T2), p(g1T2-g5T2), and p(g0T2-g6T2); (G) P3MEEMT; (H) P3MEET, P3MEEMT, and P3MEEET; and (I) ProDOT(OE)-DMP.

#### 6.3.1 Electron-Rich Backbones

##### 6.3.1.1 Benzodithiophene and Thiophene

The first report detailing the targeted synthesis of EG functionalized conjugated polymers for OECT applications came in 2016,^261^ in which a series of five thiophene- and benzodithiophene-based polymers, comprising gBDT, gBDT-T, gBDT-2T, g2T-T, and gBDT-g2T, was synthesized by conventional Stille cross-coupling polymerization. Previously, glycolated materials have been investigated in light-emitting electrochemical cells.^262^ Thiophene and benzodithiophene moieties were chosen as aromatic building blocks given their electron-rich nature and tendency to have high degrees of backbone planarity, which in turn should result in low operating voltages (necessary for stable OECT operation in the electrochemical window of water) and good electronic charge carrier transport abilities.^263−265^ For each polymer, hydrophilic triethylene glycol solubilizing chains were selected as the pendant side chains. Cyclic voltammetry and spectroelectrochemistry data revealed that variation of the aromatic building blocks comprising the conjugated polymer backbone had a substantial effect on the measured energy levels, with the recorded ionization potentials for the polymers varying between 4.38 and 4.92 eV. In particular, incorporation of the triethylene glycol substituted bithiophene unit (g2T) provided the most significant reduction of the recorded IP, with both g2T-T and gBDT-g2T featuring the lowest IP values of 4.38 and 4.43 eV, respectively. A direct consequence of g2T-T’s and gBDT-g2T’s lower IP was that during OECT operation, these polymers reached their peak transconductance within water’s electrochemical stability window, while gBDT, gBDT-T, and gBDT-2T did not. Moreover, g2T-T and gBDT-g2T also exhibited greater electrochromic responses during spectroelectrochemical measurements in a 0.1 M NaCl supporting electrolyte, in turn suggesting their increased ability to store electrical charges upon electrochemical driven oxidation. Ultimately, the lower IPs and increased charge storage abilities of g2T-T and gBDT-g2T resulted in their approximately 1–2 order of magnitude higher *g*_m_ values compared to the remaining three polymers in the series. The higher *g*_m_ of 7.9 mS recorded for g2T-T, compared to gBDT-g2T’s *g*_m_ of 0.47 mS, on the other hand, was attributed to its microstructure in the solid state, including its narrower π–π stacking distances and preferential edge-on rather than face-on orientation in GIWAXS measurements. Both of these features should favor superior electronic charge carrier mobilities in g2T-T, whereby this hypothesis was confirmed by g2T-T’s significantly higher μ of 0.28 cm^2^ V^–1^ s^–1^ compared to gBDT-g2T’s μ of 0.01 cm^2^ V^–1^ s^–1^ recorded in OECTs. The maximum *g*_m_ that could be achieved by OECTs based on g2T-T was further improved by switching from a spin-coating to a drop-casting polymer deposition process and further increasing the channel thickness. Ultimately, the maximum *g*_m_ that could be accomplished with g2T-T was around 21 mS, which at the time compared favorably with the highest performing devices based on PEDOT:PSS. Additional benefits of employing g2T-T rather than PEDOT:PSS as a channel material stemmed from its accumulation mode of behavior, allowing lower power consumptions, in combination with good switching times in the order of 1 ms.

##### 6.3.1.1.1 Glycolated Side Chains

**Figure 22:**
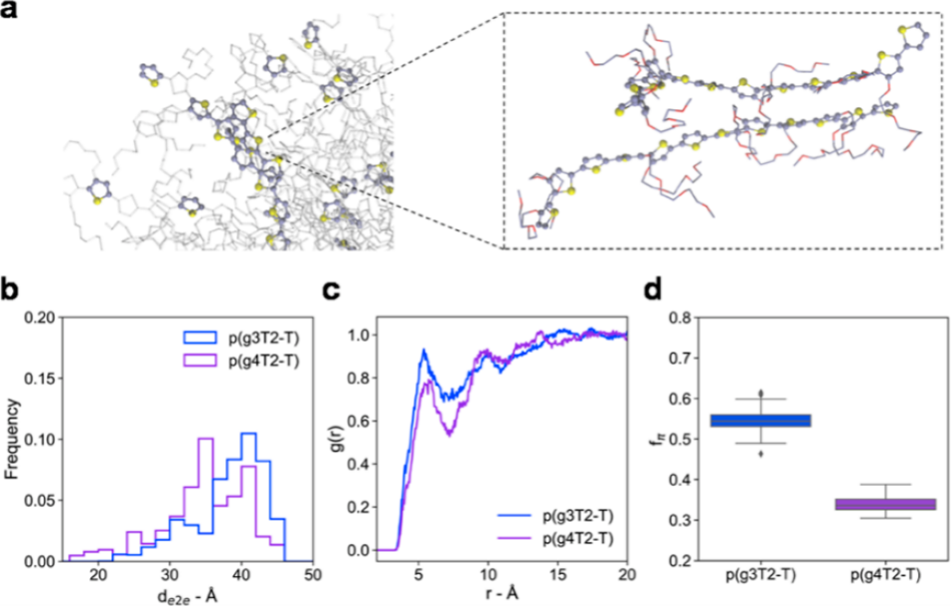
MD simulations of g2T-T and p(g4T2-T) comparing their aggregation and solid-state ordering behavior. Figure adapted with permission from ref (268). Copyright 2020 American Chemical Society.

A noteworthy feature of the investigated polymer series was that each member employed triethylene glycol solubilizing chains. Side chain engineering of conjugated polymers is however a popular chemical design strategy to maximize polymers’ performance in alternative electronic devices.^266,267^ In this context, the simplest and most straightforward means of side chain manipulation involves side chain length shortening and elongation. With this consideration in mind, the most promising member of the aforementioned polymer series, namely g2T-T, was selected and the number of ethylene glycol repeat units in its side chains modified between two to six repeat units.^268^ As anticipated, differential pulse voltammetry (DPV) indicated that side chain length modification does not have a significant impact on the conjugated polymer backbone energetics, with all polymers showing a similar onset of oxidation in aqueous media (*E*_ox,aq_) around −0.2 ± 0.1 V vs Ag/AgCl. On the other hand, both cyclic voltammetry and electrochemical impedance spectroscopy (EIS) demonstrated that side chain length engineering drastically impacts the polymers’ charging capabilities. Specifically, a careful balance in the EG side chain length must be reached; that is, the chosen EG side chain length should be sufficiently long to allow for substantial ion interaction but should be kept at a minimum length given its electrically insulating nature, thereby minimizing the polymer’s charge storage capacity. Side chain length tailoring also noticeably impacted the polymers’ structural properties, with shorter EG side chains on average incurring a more ordered microstructure as evidenced by thin-film UV–vis spectroscopy and GIWAXS measurements. Further structural evaluation of the polymers was conducted through molecular dynamics (MD) simulations of the two most promising members of the polymer series, namely g2T-T and p(g4T2-T) (Figure 22). Although g2T-T’s and p(g4T2-T)’s chemical structures only differ by one EG repeat unit length, MD simulations showed that the use of tri- rather than tetraethylene glycol-based side chains promotes more planar and extended conjugated polymer backbones and consequently also an approximately 2-fold higher fraction of π-stacking thiophene rings (2/3 for g2T-T cf. 1/3 for p(g4T2-T)), thus indicating a superior electronic charge transport in g2T-T compared to p(g4T2-T).^269^ OECTs were fabricated for all polymers, except for p(g6T2-T), whose much higher solubility resulted in significant delamination issues and prevented the fabrication of stable an OECT in aqueous media. As anticipated from the electrochemical and microstructure measurements, g2T-T exhibited the highest volumetric capacitance of 211 F cm^–3^ (cf. 8 F cm^–3^ for p(g2T2-T) and 192 F cm^–3^ for p(g4T2-T)) and highest electronic charge carrier mobility of 0.16 cm^2^ V^–1^ s^–1^ (cf. ∼10^–4^ cm^2^ V^–1^ s^–1^ for p(g2T2-T) and 0.06 cm^2^ V^–1^ s^–1^ for p(g4T2-T)), thus also resulting in its highest recorded *g*_m_ of 3.03 mS (cf. 0.12 mS for p(g2T2-T) and 0.88 mS for p(g4T2-T)). EG side chain length variation can thus have a significant impact on the OECT performance of conjugated polymers, and a careful balance in the optimum EG side chain length has to be achieved to maximize the polymer performance in devices.

After the initial publication of EG functionalized BDT-based OMIECs for OECT applications, a more detailed follow-up study evaluating their stability and performance in electrochemical devices followed.^270^ Three BDT-based polymers were synthesized, namely gBDT-T2, gBDT-TT, and gBDT-MeOT2, with each employing the same triethylene glycol substituted BDT moiety but different comonomers, specifically bithiophene (T2), thieno[3,2-*b*]thiophene (TT), and 3,3′-dimethoxy-2,2′-bithiophene (MeOT2). Preliminary CV measurements in an organic electrolyte were employed to determine the polymers’ IPs, with gBDT-T2, gBDT-TT, and gBDT-MeOT2 exhibiting IP values of 4.85, 4.70, and 4.31 eV, respectively, thus suggesting a greater propensity of the polymers to undergo oxidation upon going from a T2 to TT to MeOT2 comonomer. A direct consequence of gBDT-MeOT2’s lower IP was its ability to form polarons at lower applied potentials than gBDT-T2 and gBDT-TT. As evidenced by infrared (IR) and UV–vis spectroscopy, this in turn prevented gBDT-MeOT2 undergoing an irreversible oxidation with water when cycled within a safe electrochemical potential window up to +0.7 V, while gBDT-T2 and gBDT-TT had an increased propensity to undergo degradation reactions and form quinone-like structures. The formation of these quinone structures was detrimental for OECT operation, as it resulted in a decrease of the donor strength and a break in the effective conjugation length. An important finding from this study was, therefore, that although high-IP polymers promote oxygen and water stability in their neutral state,^210^ they concomitantly also disfavor the stability of the electrochemically oxidized state, as previously suggested in the literature.^271^ In OECTs, this behavior resulted in devices based on gBDT-MeOT2 exhibiting transconductances almost 2 orders of magnitude larger than those recorded for devices of comparable dimensions based on gBDT-T2 and gBDT-TT. Note that this is mainly due to the low doping levels that can be achieved in gBDT-T2 and gBDT-TT before the polymers degrade. It is also important to consider that, in principle, the conjugated polymer backbones of gBDT-T2 and gBDT-TT could exhibit higher OECT performances than gBDT-MeOT2, yet due to the instability of their oxidized states in water, these polymers undergo degradation reactions before this could be verified.

p(g2T-TT) is a close structural analogue of g2T-T, in which the thiophene comonomer has been substituted by a thieno[3,2-*b*]thiophene one.^272^ From a molecular design point of view, the fused and more extended nature of the thieno[3,2-*b*]thiophene unit compared to its thiophene counterpart should lead to higher conjugated backbone rigidities and, hence, also increased π–π stacking interactions and higher charge carrier mobilities. In fact, polymers making use of this design strategy had already been reported for OFET materials, for which high electronic charge carrier mobilities had been realized.^273^ The successful translation of this design concept also to OECT channel materials is well reflected in the tighter π–π stacking distance of 3.5 Å recorded for p(g2T-TT) compared to the 3.6 Å distance obtained for g2T-T, therefore also resulting in a μ improvement from 0.28 cm^2^ V^–1^ s^–1^ for g2T-T to a μ around 1 cm^2^ V^–1^ s^–1^ for p(g2T-TT).^261,272^ The superior electronic charge carrier mobilities of p(g2T-TT) were also reflected in the higher transconductance that could be achieved in devices, with p(g2T-TT) incurring a maximum *g*_m_ around 27 mS. Notably, when comparing devices of similar dimensions, the recorded transconductance for p(g2T-TT)-based devices was higher than those based on the widely available PEDOT:PSS.

##### 6.3.1.1.2 Alkyl Side Chains

Similar to a previous investigation,^189^ another fundamental aspect of this study was to also evaluate the effects of the side chain nature (either ethylene glycol- or alkyl-based) on the recorded electrochemical performance. Compared to p(g2T-TT), its alkylated counterfigure p(a2T-TT) exhibited notably different electrochemical properties. While p(g2T-TT) exhibited a highly reversible and substantial quenching of its π–π* absorption during spectroelectrochemical measurements in an aqueous 0.1 M NaCl solution, p(a2T-TT)’s electrochemical charging was irreversible, suggesting signs of either ion trapping and/or physical/chemical alteration. Electrochemical impedance spectroscopy confirmed these findings, while also demonstrating p(a2T-TT)’s significantly lower capacitive behavior compared to p(g2T-TT). Indeed, while coating and electrochemical addressing of gold electrodes with p(a2T-TT) did not significantly improve the electrode’s capacitance, an almost two-order-of-magnitude augmentation in effective capacitance could be accomplished with p(g2T-TT). Both of these aspects were attributed to the hydrophobic nature of the alkyl chains in p(a2T-TT) preventing the uptake of water, thereby creating a pathway for the ingress of ions upon electrochemical charging. The study was thus fully in line with previous literature and highlights the necessity for a certain degree of film hydration to be present to enable operation of conjugated polymers in aqueous electrolytes.^189^

**Figure 23:**
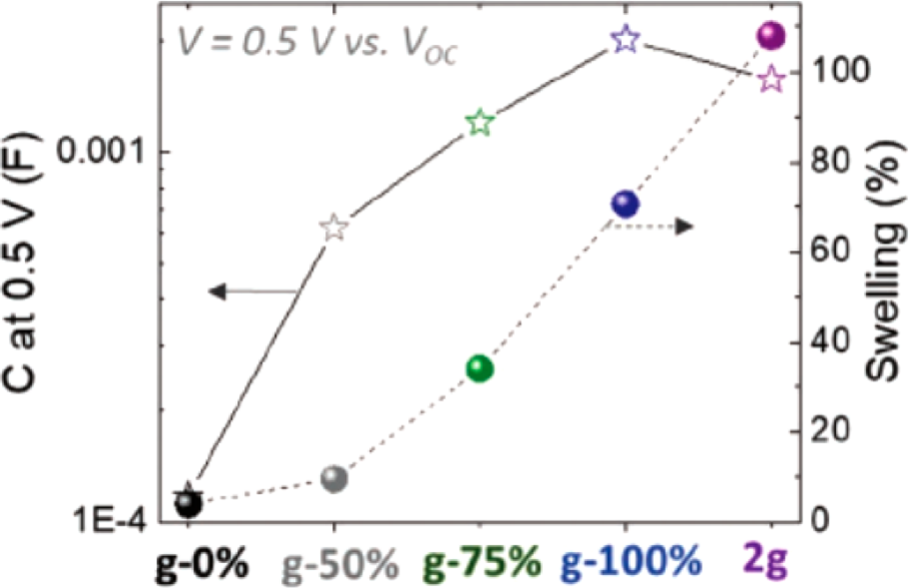
Capacitance and swelling recorded for the polymer series under the application of an applied voltage of +0.5 V versus the open-circuit potential (*V*_OC_). Figure adapted with permission from ref (274). Copyright 2020 John Wiley and Sons.

From the above investigations, it followed that hydrophilic side chains are required to facilitate ion transport to enable OECT operation in conjugated polymers based on the 2T-TT backbone. The exact degree of hydrophilic character necessary to maximize OECT performance was investigated in a recent study.^274^ Note that in this study the original p(a2T-TT) and p(g2T-TT) polymers are referred to as g-0% and g-100%. Two of the newly synthesized polymers were copolymers containing mixed fractions of g-0% and g-100%, namely g-50% and g-75%. The final member of the series was 2g, a derivative of g-100% employing hexa- rather than triethylene glycol side chains. As already determined for another EG functionalized polythiophene series,^268^ the top OECT performer of this polymer array was once again the triethylene glycol flanked g-100% polymer, incurring a maximum *g*_m_ of 18.8 mS. Increasing the hydrophobicity, on the other hand, resulted in lower *g*_m_ values, with g-0%, g-50%, and g-75% incurring *g*_m_ values of 6.9 × 10^–5^, 0.048, and 7.1 mS, respectively. Similarly, increasing the hydrophilicity also proved to be detrimental, with 2g yielding a *g*_m_ of 1.3 mS. From EIS measurements it followed that g-100%’s higher performance stemmed from its superior charge storage ability, with g-100% showing the highest C* of 295 F cm^–3^ (cf. C* values of 19, 97, 206, and 231 F cm^–3^ measured for g-0%, g-50%, g-75%, and 2g, respectively). The origin of this trend was related to the polymers’ degree of swelling as determined by electrochemical quartz crystal microbalance monitoring with dissipation (eQCM-D) experiments. While increasing the polymers’ hydrophilicity when going from g-0% to g-100% incurred progressively larger swelling and capacitance (Figure 23), further increasing the polymers’ hydrophilicity increased only the polymers’ swelling but not capacitance. The lower volumetric capacitance for 2g thus arises due to an increase in film volume, essentially increasing the volume over which the capacitance is stored and due to the increased fraction of EG side chains, which dilute the fraction of electroactive polymer present.^197^ Ultimately, this study indicates that while hydrophilic side chains are required for polymer swelling, water ion uptake, and therefore, also ion transport into the organic semiconductor to enable OECT operation, excessive swelling can also dilute the fraction of electronic material present, thus resulting in lowered OECT performances.

##### 6.3.1.2 Thiophene-Only Backbones

##### 6.3.1.2.1 Head-to-Head and Head-to-Tail Backbones

**Figure 24:**
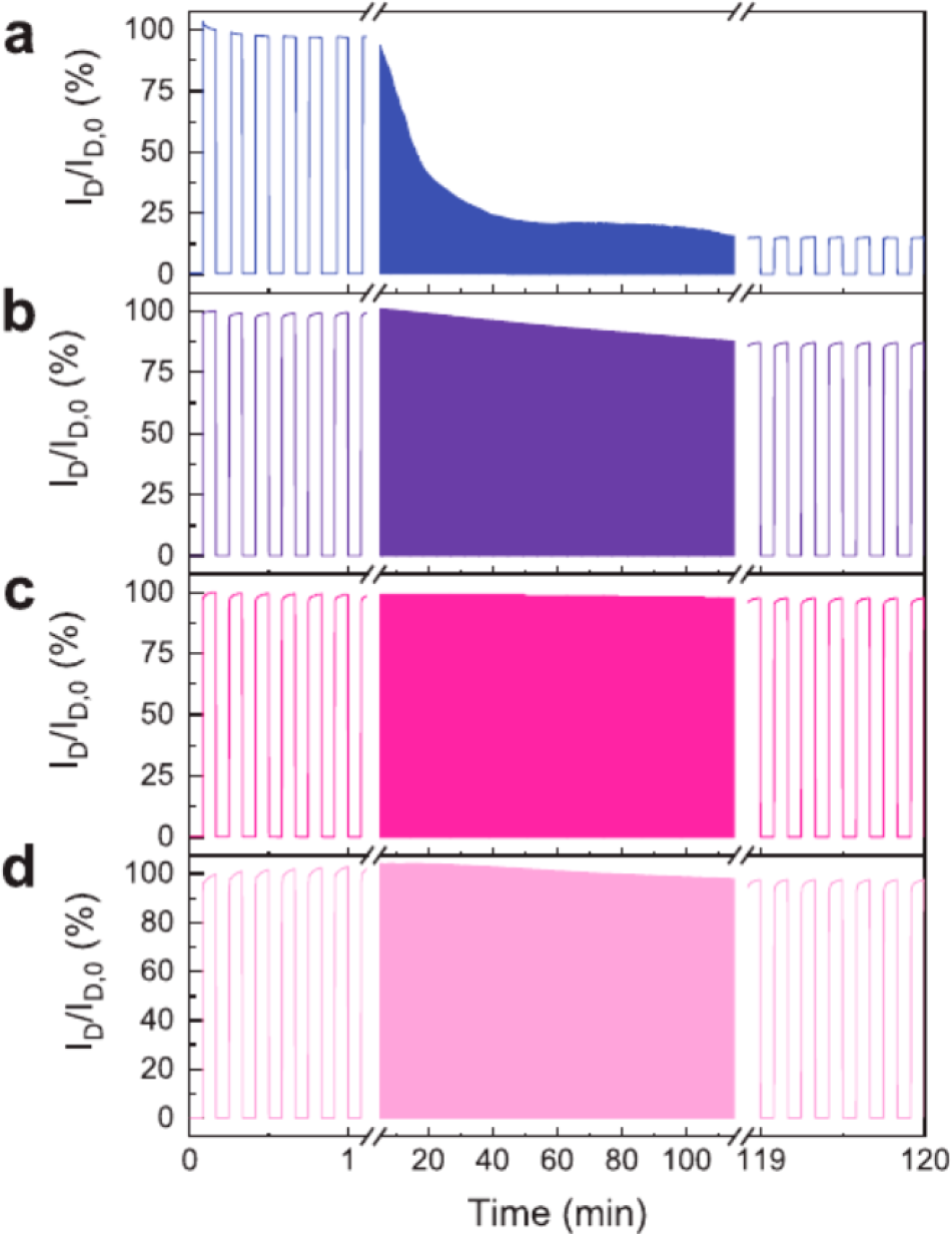
Electrochemical cycling stability recorded for p(g3T2), p(g2T2-g4T2), p(g1T2-g5T2), and p(g0T2-g6T2) over 2 h of continuous addressing. Figure adapted with permission from ref (277). Copyright 2020 John Wiley and Sons.

A conjugated polymer making use of the same triethylene glycol functionalized bithiophene unit as employed in g2T-T and p(g2T-TT) is the homopolymer p(g3T2). p(g3T2) has received attention for a wide array of bioelectronic applications, including for electrochemical actuators and batteries operating in aqueous saltwater solutions.^197,275,276^ Recently, a series of polymers based on p(g3T2), which only differ in the distribution of their ethylene glycol chains that are tethered to the conjugated backbone, have been designed specifically for OECT applications; see Figure 21.^277^ Note that the average number of EG repeat units per thiophene ring remained constant (three) across the series in this design strategy. From a chemical design point of view, this polymer series aimed to incorporate several design elements outlined in previous studies that yielded superior OECT performances. Specifically, an average EG length of three was chosen to maximize the resulting OECT performance and ensure good polymer solubility.^268,274^ The presence of a glycoxy substituent on each thiophene ring was also deemed to be beneficial toward electrochemical cycling stability, with the lone pair of electrons on the oxygen atoms grafted onto the conjugated polymer backbone being able to donate electron density through a mesomeric effect, thus reducing the polymers’ IP and stabilizing the polymers’ charged species.^270^ Moreover, the added steric bulk from the glycoxy substituents should also help in shielding the conjugated backbone from interfering chemical species, thereby also contributing to an increased device stability. In OECTs, devices based on each polymer performed well, incurring transconductance values of 8.9, 6.5, 10.2, and 8.1 mS for p(g3T2), p(g2T2-g4T2), p(g1T2-g5T2), and p(g0T2-g6T2), respectively. Extraction of the polymers’ μC* gave values of 161, 522, 496, and 302 F cm^–1^ s^–1^ V^–1^ for the highest performing channel of p(g3T2), p(g2T2-g4T2), p(g1T2-g5T2), and p(g0T2-g6T2), respectively. The difference in OECT steady-state performance recorded across the four polymers was ascribed to the polymers’ different degrees of swelling, with p(g2T2-g4T2) and p(g1T2-g5T2) incurring the best balance of sufficient swelling to maximize the polymers’ capacitive properties, yet not excessive swelling, which as previously highlighted, can result in a decrease of both μ and C*.^274^ In terms of electrochemical cycling stability, the polymers were cycled for 2 h across two voltage limits, one corresponding to the “OFF” state and one corresponding to the “ON” state of the devices. Note that in this study the gate and drain voltage combination defining the “ON” state was the one incurring the highest performance, i.e. transconductance. After 2 h of electrochemical cycling, p(g3T2), p(g2T2-g4T2), p(g1T2-g5T2), and p(g0T2-g6T2) retained 15%, 87%, 98%, and 98% of their initial currents, with the values incurred for p(g1T2-g5T2) and p(g0T2-g6T2) being among the highest reported for any EG functionalized conjugated polymer (Figure 24). This aspect is particularly important when considering the polymers’ applicability in biomedical devices, where long-term device stability is of utmost importance. The electrochemical stability trend of the polymers was closely in line with the polymers’ degree of swelling upon electrochemical addressing, thus suggesting that lower degrees of electrochemical swelling, resulting in reduced volumetric changes upon repeated electrochemical addressing, may aid electrochemical device stability by minimizing any morphological changes and/or substrate delamination that the polymers undergo.

##### 6.3.1.2.2 Head-to-Tail Analogs

While adjacent thiophene rings functionalized with EG side chains at the 3-position can be coupled in a head-to-head or tail-to-tail fashion as shown above, another arrangement involves their head-to-tail arrangement, as in P3HT,^278^ as is the case in P3MEEMT;^279^ see Figure 21. Synthetically, P3MEEMT was prepared as previously outlined through a Grignard metathesis (GRIM) polymerization,^280^ thus foregoing the need of employing an expensive palladium transition metal catalyst and highly toxic organostannane species during its synthesis. P3MEEMT has also been prepared by electrochemical polymerization methods in the past, thus highlighting its compatibility to be made through a range of synthetic methods.^280^ When tested in OECTs using a 0.1 M aqueous potassium chloride solution as the supporting electrolyte, as cast P3MEEMT films were determined to lead to a μC* value of 49.1 ± 5.0 F cm^–1^ V^–1^ s^–1^. For many polymers used in OFET devices, thermal annealing is employed to increase the crystallinity and in turn the electronic charge carrier mobility of the resulting polymer.^281−283^ In an attempt to translate this strategy to OECT technologies, the authors annealed P3MEEMT films at 125, 145, and 165 °C. Progressively increasing the annealing temperature was, however, found to incrementally decrease the recorded μC* value, with the highest annealing temperature also incurring the lowest μC* of 20.9 ± 1.1 F cm^–1^ V^–1^ s^–1^. C* values were found to be unaffected by the annealing treatment, thus indicating that annealing had predominantly exerted a negative impact on P3MEEMT’s μ. This, in turn, was ascribed to the annealing treatment incurring more crystalline polymer films, which despite incurring a higher OFET mobility in their dry state, were more prone to intergrain disruption during water uptake throughout electrochemical doping.

A follow-up study on P3MEEMT involved modification of its methyl spacer between the conjugated polymer backbone and the solubilizing diethylene glycol side chains.^284^ While in one of the synthesized derivatives, P3MEET, the methyl linker was removed, in P3MEEET, the methyl linker was extended to become an ethyl one. In devices using a 0.1 M aqueous NaCl solution as the supporting electrolyte, P3MEET, P3MEEMT, and P3MEEET gave μC* values of 0.04, 9.8, and 11.5 F cm^–1^ V^–1^ s^–1^, respectively. EIS measurements demonstrated a linear increase in the obtained C* values upon going from P3MEET to P3MEEMT to P3MEEET, with P3MEET, P3MEEMT, and P3MEEET yielding C* of 80 ± 9, 160 ± 12, and 242 ± 17 F cm^–3^, respectively. The low charge storage capacity of P3MEET was compounded by its poor charge carrier mobility of only 5.2 × 10^–4^ cm^2^ V^–1^ s^–1^. P3MEEMT and P3MEEET, on the other hand, gave similar hole mobilities of 0.06 and 0.05 cm^2^ V^–1^ s^–1^ respectively.

Alternative environmentally benign polymerization techniques to synthesize conjugated polymers for OECT applications have also been employed. Direct heteroarylation polymerization (DHAP) is a type of palladium catalyzed polymerization reaction that enables the formation of C–C bonds between a halogenated heteroarene and a heteroarene bearing activated C–H bonds, while foregoing the need to synthesize organometallic intermediates.^285,286^ Indeed, DHAP has been shown to be a successful tool for synthesizing conjugated polymers for alternative organic electronic applications with performances comparable to those as synthesized by conventional (e.g. Stille or Suzuki) cross-coupling polymerization methods.^287,288^ ProDOT(OE)-DMP is an example of an EG functionalized conjugated polymer that has been synthesized through such an approach and successfully employed as an OECT channel material; see Figure 21.^289^Figure 21 also displays the chemical structure of ProDOT(OE)-DMP’s repeat unit that consists of two different 3,4-propylenedioxythiophene (ProDOT) rings, one bearing two pendant triethylene glycol side chains on its bridgehead carbon atom and one bearing a 2,2-dimethyl bridge. OECTs fabricated with ProDOT(OE)-DMP incurred a maximum *g*_m_ of 0.62 mS. Although this value is slightly lower compared to OECTs based on other EG functionalized polythiophene-based polymers, the transistor aspect ratio (*W*/*L*) used in this study was 1 rather than the more common aspect ratio of 10, which has indeed also been employed during the performance evaluation of most other EG functionalized polythiophene polymers. Theoretically, the maximum *g*_m_ that can be incurred for ProDOT(OE)-DMP-based devices with devices employing an aspect ratio of 10 should thus be closer to 6 mS, which compares significantly more favorably against other EG functionalized polymers.

#### 6.3.2 Donor–Acceptor Backbones

##### 6.3.2.1 TFT

**Figure 25:**
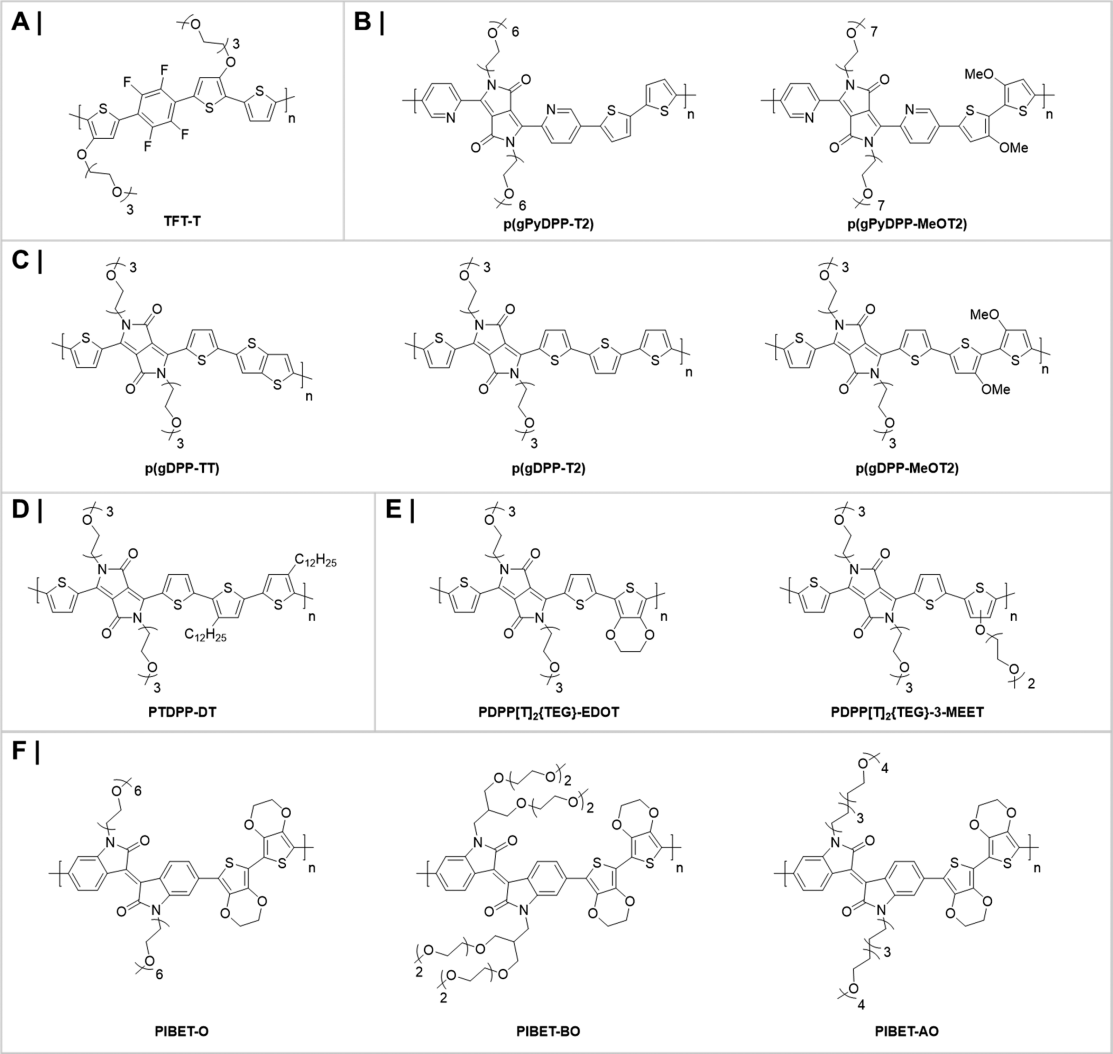
Chemical structures of (A) TFT-T; (B) p(gPyDPP-T2) and p(gPyDPP-MeOT2); (C) p(gDPP-TT), p(gDPP-T2), and p(gDPP-MeOT2); (D) PTDPP-DT; (E) PDPP[T]_2_{TEG}-EDOT and PDPP[T]_2_{TEG}-3-MEET; and (F) PIBET-O, PIBET-BO, and PIBET-AO.

DHAP was also employed as a methodology to synthesize another series of conjugated polymers based on a glycolated 1,4-dithienyl-2,3,5,6-tetrafluorophenylene (TFT) unit, copolymerized with either benzene, thiophene, thieno[3,2-*b*]thiophene, bithiophene, or tetrafluorophenylene, to produce the polymers TFT-P, TFT-T, TFT-TT, TFT-2T, and TFT-F, respectively; see Figure 25.^290^ From a molecular design point of view, the use of thiophene-based and TFT building blocks should result in the formation of highly planar conjugated polymer backbones that should be beneficial toward high electronic charge carrier transport. Similarly, the use of noncovalent attractive intramolecular S–F, S–O, and F–H interactions should further promote planarity along the conjugated polymer backbone.^291−293^ The more electron deficient nature of the TFT unit should also lead to reduced ionization potentials and, therefore, a reduced susceptibility of the polymers to reactions with oxygen under ambient conditions.^210^ The successful outcome of achieving planar conjugated polymer backbone conformations was highlighted by DFT simulations, with each polymer incurring a nearly planar dihedral angle within not only the TFT subunit but also the C–C bond connecting the TFT moiety to the respective comonomer. The only exception to this trend was the TFT-F polymer, for which a 36° dihedral angle was calculated across the C–C bond connecting the TFT and F building blocks. Here steric clashes and electrostatic repulsions between the fluorine and oxygen atoms on adjacent glycolated thiophene and tetrafluorophenylene units resulted in a twisted conjugated polymer backbone, which was expected to be detrimental toward electronic charge carrier transport both intra- and intermolecularly. Initial evaluation of the polymers’ electrochemical stabilities was conducted by repeated CV measurements in an aqueous electrolyte. Here, only the TFT-T polymer, featuring the highest side chain density per conjugated polymer backbone repeat unit length and one of the lowest onsets of oxidation, incurred a reversible oxidation process that was stable over multiple switching cycles. OECT evaluation of TFT-T was subsequently conducted, with devices incurring a peak *g*_m_ of 0.17 mS and a μC* product of 10 F cm^–1^ V^–1^ s^–1^.

##### 6.3.2.2 DPP

Similarly to TFT-T, whose conjugated polymer backbone can be described as comprising an electron-rich donor component (thiophene) and an electron-deficient acceptor moiety (1,4-dithienyl-2,3,5,6-tetrafluorophenylene), the two polymers p(gPyDPP-T2) and p(gPyDPP-MeOT2) can also be classified as donor–acceptor (D-A) copolymers, with the pyridine flanked diketopyrrolopyrrole (DPP) building block being the acceptor and the bithiophene or 3,3′-dimethoxybithiophene unit acting as the donor.^294^ When tested in OECTs, p(gPyDPP-MeOT2) was able to exhibit a maximum stable transconductance of 1.4 mS at a *V*_G_ of −0.7 V, a μ of 0.030 ± 0.007 cm^2^ V^–1^ s^–1^, and a C* of 60 F cm^–3^. On the other hand, due to insufficiently high device stabilities, it was not possible to fully evaluate the OECT performance of p(gPyDPP-T2). The low device stability in p(gPyDPP-T2) was attributed to its significantly lower IP of 5.5 eV compared to the IP of 5.0 eV recorded for p(gPyDPP-MeOT2), thus lying outside the electrochemical stability window of water and leading to a less stabilized oxidized form of the polymer. Moreover, the absence of any methoxy groups at the 3,3′ positions in the T2 unit of p(gPyDPP-T2) was also suggested to potentially lead to a reduced steric barrier around the conjugated polymer backbone, thus rendering it more susceptible to interfering side reactions occurring during electrochemical doping. Although the OECT performance of these D-A copolymers is, therefore, lower compared to their all-donor counterparts, further electrochemical evaluation of p(gPyDPP-MeOT2) highlighted alternative benefits incurred by resorting to a D-A conjugated polymer backbone, such as the avoidance of faradaic side reactions during OECT operation. In particular, it was found that compared to OECT materials with more electron-rich conjugated polymer backbones and hence lower IPs (e.g. PEDOT:PSS and p(g2T-TT)), the reduced IP value in p(gPyDPP-MeOT2) meant that this material was able to avoid the oxygen reduction reaction (ORR) from occurring during device operation. Preventing the ORR from occurring during device operation is particularly important in the context of biological interfacing of OECTs due to the tendency of organic materials to favor the formation of hydrogen peroxide as a product, which can lead to corrosive damage occurring to the device materials or the biological tissue under analysis.^295−297^ Following an extensive set of ORR studies, it was determined that designing OMIECs with IP values ≥ 4.9 eV is an effective strategy to avoid the ORR from occurring, as this results in a thermodynamic barrier for electron transfer to occur from the polymers’ HOMO in their undoped state, thus highlighting the advantages conferred by a D-A conjugated polymer backbone.

Recently, the performance of D-A conjugated polymers for OECT applications was significantly improved, with μC* values as high as 342 ± 35 F cm^–1^ V^–1^ s^–1^ having been reported through the synthesis of an alternative series of DPP-based conjugated polymers; see Figure 25.^298,299^ Compared to the previously developed pyridine flanked DPP-based polymers, the newly designed polymers made use of a thiophene flanked DPP core, a chemical motif which has frequently resulted in materials with excellent charge carrier transport abilities in alternative electronic devices.^237,300,301^ Variation in the polymers’ comonomer, either bithiophene, thieno[3,2-*b*]thiophene, or 3,3-dimethoxybithiophene, was subsequently shown to have a significant impact on the polymers’ OECT performance, with p(gDPP-TT), p(gDPP-T2), and p(gDPP-MeOT2) incurring a μC* of 125 ± 22, 342 ± 35, and 57 ± 5 F cm^–1^ V^–1^ s^–1^, respectively. While the volumetric capacitance of the polymers was not significantly affected by comonomer choice, with all polymers yielding C* values between 169 and 196 F cm^–3^, the use of bithiophene and thieno[3,2-*b*]thiophene, whose energy levels were more similar compared to the thiophene flanked DPP moiety, was found to benefit the polymers’ μ. For these systems, the hole polaron was determined to be spread to a greater extent and more evenly across the conjugated polymer backbone, thus leading to lower polaron binding energies and allowing for a larger surface area for charges to hop between adjacent polymer chains, in turn favoring both intra- and intermolecular charge carrier transport.^302^ In addition to a more delocalized hole polaron, p(gDPP-TT) and p(gDPP-T2) were also found to possess a higher degree of molecular ordering and increased π–π stacking coherence lengths, which may also contribute to their improved mobilities. The high performances of p(gDPP-TT) and p(gDPP-T2) are noteworthy when also considering that these polymers featured an IP > 4.9 eV, therefore suggesting that these materials should also thermodynamically disfavor any spontaneous ORR, which is beneficial in terms of device stability.

In addition to the three aforementioned polymers making use of the thiophene flanked DPP comonomer, several alternatives making use of different comonomers have also been developed, such as those illustrated in Figure 25. PTDPP-DT, originally developed for organic light-emitting electrochemical cells and transistors making use of ionic liquid or solid-state electrolytes,^303^ was subsequently also evaluated in aqueous-electrolyte-gated OECTs.^304^ When using an aqueous 0.1 M sodium chloride solution as the supporting electrolyte, a peak *g*_m_ value of 7.2 ± 2.9 mS and a maximum μC* of 149 ± 61 F cm^–1^ V^–1^ s^–1^ could be achieved, thus comparing favorably with alternative high-performance DPP-based D-A copolymers and all-donor polymers. Importantly, PTDPP-DT also showed good electrochemical cycling stability, retaining a large proportion of the initial drain current over a ∼30 min time span. Substituting the 0.1 M aqueous sodium chloride electrolyte solution for a 0.1 M aqueous solution based on the ionic liquid, 1-ethyl-3-methylimidazoliumtetrafluoroborate (EMIM BF_4_), was found to significantly improve the maximum *g*_m_ and μC* that could be attained, incurring values of 21.4 ± 4.8 mS and 559 ± 65 F cm^–1^ V^–1^ s^–1^, respectively. As previously outlined in alternative studies evaluating the effects of anion substitution for improving the OECT performance of p-type conjugated polymers,^305^ the authors suggested a more efficient doping process by the more hydrophobic and larger BF_4_^–^ anion compared to Cl^–^, thus leading to approximately 2-fold larger volumetric capacitance and OECT mobility values. While representing a suitable means to maximize device performance, one limitation of this approach is its limited applicability for neural and biological sensing applications, as chloride ions tend to constitute the largest proportion of the anions present within the biological tissue under analysis.^306,307^

##### 6.3.2.3 Isoindigo

PDPP[T]2{TEG}-EDOT and PDPP[T]2{TEG}-3-MEET have recently been synthesized, making use of a 3,4-ethylenedioxythiophene and 3-[2-(2-methoxyethoxy)ethoxy]thiophene comonomer, respectively.^308^ Performance evaluation in devices exhibited comparable *g*_m_ (1.4 and 1.9 mS for PDPP[T]2{TEG}-EDOT and PDPP[T]2{TEG}-3-MEET, respectively) and μC* (14 and 45 F cm^–1^ V^–1^ s^–1^ for PDPP[T]2{TEG}-EDOT and PDPP[T]2{TEG}^−3^-MEET, respectively), whereby these values are slightly lower compared to the ones reported for the aforementioned thiophene flanked DPP-based polymers. Nonetheless, both polymers exhibited good electrochemical cycling stabilities, while also showing excellent cell viability, thus rendering these materials particularly interesting for long-term cellular interfacing.

Isoindigo (IID) has also been explored as an alternative acceptor unit compared to DPP. Similarly to the DPP-based polymers, IID-based polymers have also been shown to incur good electronic charge carrier transport properties in alternative electronic devices,^309−311^ hence prompting for their exploration in OECT applications. Figure 25 displays a series of IID-based polymers synthesized for OECT devices, in which the nature of the pendant ethylene glycol-based side chains was altered from a linear all-ethylene-glycol-based side chain (PIBET-O) to a branched all-ethylene-glycol-based side chain (PIBET-BO) and to a linear mixed alkyl-ethylene glycol side chain (PIBET-AO).^312^ Analogously to the previously synthesized DPP polymers, PIBET-O, PIBET-BO, and PIBET-AO were also synthesized through Stille cross-coupling polymerization. Energy level analysis of the polymers revealed no significant difference in the recorded IP, with all polymers incurring an IP ∼ 4.9 eV, thus also suggesting the suitability of the polymers to be doped stably and reversibly within the electrochemical doping window of water. OECTs were fabricated on interdigitated gold microelectrodes on glass, featuring a channel width of 39000 μm, length of 20 μm, and comparable thicknesses between 48 and 66 nm. Average transconductance values of 10.6 ± 2.3, 5.1 ± 2.1, and 12.4 ± 1.7 mS were recorded for PIBET-O, PIBET-BO, and PIBET-AO, suggesting a PIBET-AO > PIBET-O > PIBET-BO performance order, which was confirmed when normalizing the recorded *g*_m_ values by the channel geometry. The superior performance of the linear side chain-containing polymers, PIBET-AO and PIBET-O, was hypothesized to be a result of an additional semicrystalline polymorph in their crystalline phases that featured a significantly narrower π–π stacking distance, ∼3.3 Å, compared to the main scattering feature, ∼3.9 Å, thereby boosting intermolecular charge carrier transport. One additional benefit conferred by the hybrid alkyl-ethylene glycol side chains in PIBET-AO was its significantly higher OECT electrochemical cycling stability, with no decrease in the original drain current noticeable over 6 h of continuous on–off switching. In comparison, PIBET-O and PIBET-BO were only able to retain 10% of their initial drain current after 40 and 6 min of operation, respectively. Given the polymers’ similar energy levels, the difference in device stability was ascribed due to the polymers’ varying hydrophilicity and, hence, tendency to undergo volumetric swelling upon aqueous electrolyte exposure and device operation, with the most hydrophobic polymer (PIBET-AO) yielding the highest stability and the most hydrophilic polymer (PIBET-BO) the lowest one.

Rather than employing EG side chains to promote mixed conduction behavior into CPs, a recent study investigated the use of hydrophilic hydroxylated alkyl side chains in a thiophene-based polymer, P3HHT,^313^ which had previously been demonstrated to be suited for conducting ions in aqueous media.^314^ The rationale for employing such side chains for OECT devices was their reduced tendency for water uptake compared to the commonly employed EG motifs, while still ensuring sufficient ion conductivity. This concept was confirmed through detailed eQCM-D measurements, in which P3HHT was found to only swell by 2.4% passively (in the absence of an applied bias) and 9% actively (in the presence of an applied doping bias) in a 0.1 M aqueous KCl solution. In comparison, the reference p(g2T-TT) polymer functionalized with the common triethylene glycol side chains was found to swell between 10 and 15% passively and 75 and 80% actively. In devices, P3HHT was found to incur a μC* of 35 F cm^–1^ V^–1^ s^–1^ and, thus, comparable to the performance typically incurred by PEDOT:PSS, therefore highlighting the suitability of this design strategy to afford relatively high-performance materials for OECT applications. One drawback of the reduced water uptake by P3HHT in OECTs was the necessity to employ rather slow scanning rates of 0.25 V s^–1^ to minimize hysteresis, thus highlighting the careful trade-off that has to be achieved in terms of water uptake.

#### 6.3.3 Electron-Poor Backbones

##### 6.3.3.1 NDI-gT2

**Figure 26:**
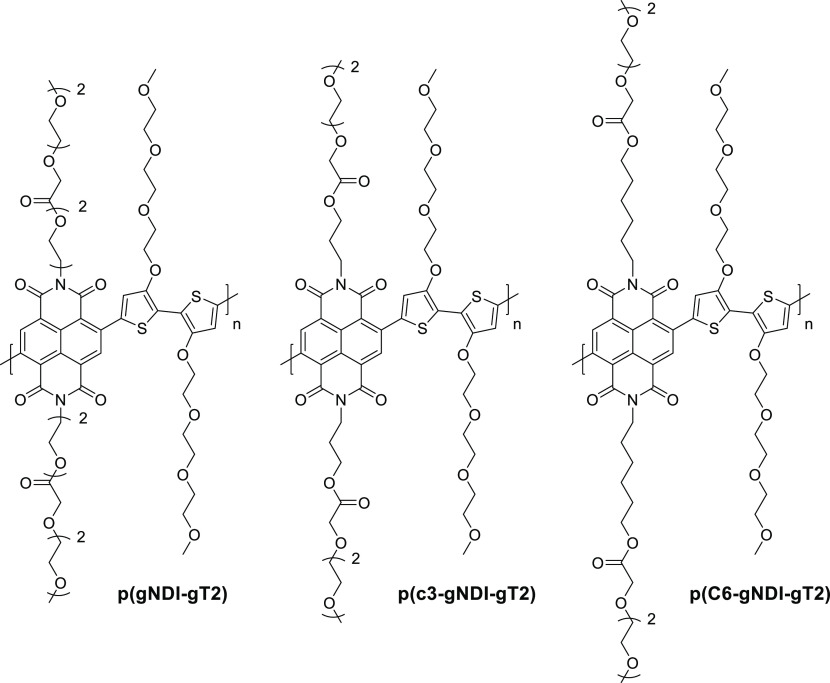
Chemical structures of p(gNDI-gT2), p(C3-gNDI-gT2), and p(C6-gNDI-gT2).

In addition to p-type semiconducting polymers, the development of high-performance and high-stability n-type materials is also crucial in the context of developing complementary circuitry and for specific biological applications of OECTs, such as enzymatic activity recording, which relies on electron rather hole transfer.^228,315,316^ The first example of an n-type OECT channel material was published in 2016,^317^ namely p(gNDI-gT2), whose conjugated polymer backbone employed alternating naphthalene diimide (NDI) and 3,3′-dimethoxybithiophene units; see Figure 26. In this study, the authors outlined that for the successful development of an n-type OECT material, the material itself must be able to undergo reversible electrochemical reduction within the electrochemical stability window of water, i.e. possess a high electron affinity (EA > 4.0 eV) and the capacity for facile ion penetration. Cyclic voltammetry highlighted p(gNDI-gT2) to fulfill both of these criteria (recorded EA = 4.12 eV), therefore demonstrating its suitability for n-type charge carrier transport. In parallel, the strongly electron donating nature of the gT2 comonomer also imparted the polymer with a rather shallow IP of 4.83 eV and, therefore, its ability to undergo stable p-type doping within the electrochemical stability window of water. p(gNDI-gT2)’s ambipolar character was further demonstrated during OECT operation, where balanced peak *g*_m_ values of 21.7 and 13.4 μS were reported for its n-type and p-type modes of operation, respectively. From EIS analysis a high volumetric capacitance of 397 F cm^–3^ was extracted, thus suggesting the relatively low OECT performance of p(gNDI-gT2) in comparison to its p-type counterparts to stem from low electron charge carrier mobility. This hypothesis was confirmed by OFET electron mobility measurements, which incurred an electron mobility of 1.0 × 10^–5^ cm^2^ V^–1^ s^–1^ for p(gNDI-gT2).

To improve the OECT performance of p(gNDI-gT2), a recent investigation evaluated the effects of side chain manipulation, in which the all-ethylene-glycol side chains in p(gNDI-gT2)’s NDI unit were replaced by hybrid alkyl-glycol side chains, bearing a propyl or hexyl spacer to afford p(C3-gNDI-gT2) and p(C6-gNDI-gT2), respectively; see Figure 26.^318^ As anticipated, introduction of the hydrophobic spacers into the polymers’ side chains did not significantly affect the polymers’ energy levels, with all polymers incurring a similar EA around 4.0 eV. Nonetheless, polymer swelling analysis through quartz crystal microbalance with dissipation monitoring (QCM-D) during and in the absence of an electrochemical doping bias highlighted that alkyl spacer inclusion is a successful strategy for minimizing the degree of swelling of the polymer films. In particular, the order of both passive and active swelling was p(gNDI-gT2) > p(C3-gNDI-gT2) > p(C6-gNDI-gT2). This trend was also used to explain the recorded OECT performance of the polymers, with p(gNDI-gT2), p(C3-gNDI-gT2), and p(C6-gNDI-gT2) incurring μC* values of 0.06, 0.13, and 0.16 F cm^–1^ V^–1^ s^–1^, respectively, thus confirming the benefits of alkyl spacer inclusion. p(C3-gNDI-gT2) and p(C6-gNDI-gT2)’s reduced propensity to undergo volume changes during electrochemical addressing also benefited the polymers’ electrochemical stabilities over the p(gNDI-gT2) reference, with devices based on the two polymers containing the aliphatic spacers retaining higher current retentions during long-term electrochemical cycling experiments. Similar concepts highlighting the need to carefully balance the proportion of ethylene glycol to alkyl side chain fractions have also been explored in related NDI-T2-based conjugated polymers for OECT applications.^43,319^

##### 6.3.3.2 PgNgN and PgNaN

**Figure 27:**
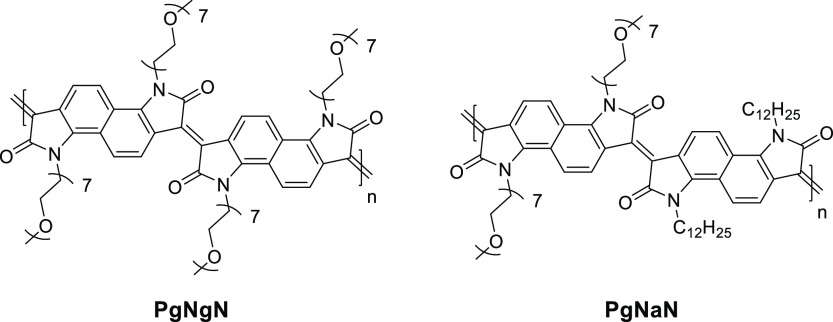
Chemical structures of PgNgN and PgNaN.

Several studies have highlighted the benefits of reducing the donor character in donor–acceptor conjugated polymers or alternatively using all-acceptor-based conjugated polymer backbones to improve the n-type performance of conjugated polymers in electronic devices.^320−324^ Two conjugated polymers making use of such all-acceptor backbones are PgNgN and PgNaN, shown in Figure 27, which were synthesized by a cheap and nontoxic transition metal-free acid catalyzed aldol polycondensation reaction.^325^ A direct consequence of this polymerization method is the formation of C=C double bonds rather than C–C single bonds between adjacent naphthalene bislactam rings, thereby giving rise to virtually torsion-free π–π backbones that benefit the delocalization of the LUMO and favor their electronic charge carrier transport properties.^322,326^ In fact, OECT charge carrier mobility evaluation of the two polymers revealed PgNaN to incur an electron mobility of 6.50 × 10^–3^ cm^2^ V^–1^ s^–1^, which is one of the highest reported for n-type OECT channel materials. In contrast, PgNgN, which solely makes use of ethylene glycol side chains, gave a lower value of 1.89 × 10^–4^ cm^2^ V^–1^ s^–1^. The superior electronic charge carrier mobility of PgNaN was ascribed due to a combination of its higher molecular weight, propensity to orient itself in a mixed edge- and face-on orientation onto substrates, and larger coherence length of its π-stacks. The presence of ∼50% ion impermeable alkyl solubilizing chains in PgNaN, however, led to a rough halving of its C* (100 F cm^–3^) compared to PgNgN (239 F cm^–3^). Ultimately, devices based on PgNaN and PgNgN resulted in μC* values of 0.652 and 0.046 F cm^–1^ V^–1^ s^–1^, respectively, thus making PgNaN one of the highest performing n-type OECT materials to date.

##### 6.3.3.3 Poly(benzimidazobenzophenanthroline)

**Figure 28:**
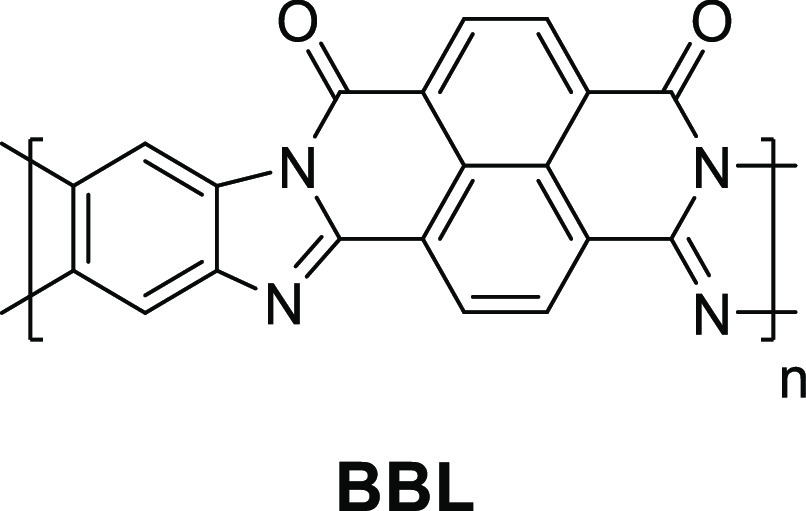
Chemical structure of BBL.

Poly(benzimidazobenzophenanthroline), BBL (Figure 28), is a “side chain-less” ladder-type polymer, which has shown considerable success as active material for organic field-effect transistors and thermoelectric applications.^323,327,328^ Given its good electronic charge carrier mobility and large EA, BBL was also envisaged to be suitable for OECT applications. In devices featuring a length of 20 μm, a width of 39 mm, and a thickness of 180 nm, spray-coated films of BBL incurred a large maximum transconductance of 9.7 mS.^228^ One particular property contributing to BBL’s high performance in devices was its excellent C* value of 930 F cm^–3^, which is speculated to be due to its lack of any electrically insulating side chains. In parallel, BBL-based OECTs also incurred stable operation during 1 h of successive gate voltage pulses and after three months of shelf storage in ambient atmosphere, further highlighting its suitability for its use in electronic devices. To illustrate the utility of BBL, a complementary inverter making use of a BBL-based OECT n-channel and a P3CPT-based OECT p-channel was fabricated, whereby relatively high gain values in excess of 11 were recorded. Despite representing a valuable contribution to advancing the performance and stability of n-type OECTs, BBL-based OECTs suffer from rather slow switching speeds ∼ 0.5 s and the necessity to be processed from highly acidic methanesulfonic acid solutions.

## 7 Summary

Various clinical drivers exist for the development of devices for electronic neural interfacing. Improvements in signal processing and conventional microelectronic back ends of neurophysiological equipment mean that the bottleneck is increasingly related to the quality of the tissue–electrode interface. This has led to the use of organic polymers as coatings for passive electrodes and as the material in active devices, such as electrochemical transistors. Broadly, this is due to their ease of processing, patterning, chemical modification, and property tuning and, finally, very favorable and unique properties that favor electrically desirable, low-impedance tissue–electrode interfaces, such as volumetric capacitance.

Currently, the literature on semiconducting polymers for neural applications is dominated by PEDOT and its derivatives, along with nitrogen-containing polymers, such as PPy. This has been to a large extent driven by the ease of deposition onto electrodes and accessibility of the materials. Currently, polymers of a variety of different backbones are explored for use in OECTs—this includes synthetically novel entities as well as polymers previously used for other types of devices, such as photovoltaics or LECs. This increase in variety has the potential to expand the options for researchers on the device-engineering side of neural electronics.

The broad range of frequencies and powers of interest in different neural interfacing setups, such as low frequency in EEG and high frequency in single-unit recording, provides a rich testing ground for novel polymers and many opportunities for application. Additionally, the complex character of neural interfacing often means that parameters other than electrical figures of merit of a polymer (such as mobility, charge carrier sign and density, and volumetric capacitance) can often be crucial to the application. Such crucial auxiliary properties are adhesion, biocompatibility, and mechanical compatibility.

While electronical characterization of semiconducting polymers has become increasingly standardized, with the adoption of μ and C* as figures of merit, the characterization of many of the aforementioned auxiliary properties has not kept up with the pace. We have made some suggestions for similar ways to benchmark biocompatibility (e.g., by using established ISO standards for biocompatibility) and have pointed the reader to sources on mechanical characterization. The large set of desirable properties, coupled with the large chemical space of synthetically accessible organic semiconducting polymers, maked this topic promising for synthetic, physicochemical, or applied engineering studies.
